# Perceptions and attitudes of Small Animal Internal Medicine specialists toward the publication requirement for board certification

**DOI:** 10.1111/jvim.15717

**Published:** 2020-02-07

**Authors:** Adam J. Birkenheuer, Kenneth D. Royal, Anthony Cerreta, Daniel Hemstreet, Katharine F. Lunn, Jody L. Gookin, Stephanie McGarvey

**Affiliations:** ^1^ Department of Clinical Sciences North Carolina State University, College of Veterinary Medicine Raleigh North Carolina

**Keywords:** board certification, credentials, education, residency training

## Abstract

**Background:**

The publication requirement for board certification in Small Animal Internal Medicine (SAIM) by the ACVIM is controversial.

**Objectives:**

Directly and indirectly evaluate the perceptions SAIM Diplomates have on the publication requirement. A secondary objective was to compare the frequency with which publications submitted for credentialing purposes (CredPubs) were cited compared to control articles.

**Subjects:**

One thousand two hundred forty‐one SAIM Diplomates were sent an electronic survey.

**Methods:**

A electronic survey was sent to all SAIM Diplomates. Practice websites were evaluated for reference to publication or research. An electronic database was searched to identify the number of times a subset of CredPubs were cited was compared to control articles.

**Results:**

Five hundred six individuals responded. The majority of respondents (n = 428, 85.25%) stated the requirement should be retained either with no changes (n = 186, 37.05%) or with clarifications or modifications (n = 242, 48.21%). A minority of respondents (n = 74, 14.7%) felt it should be eliminated. “Understanding the scientific process” was the most commonly selected reason (n = 467, 92.48%) for the publication requirement. All websites that mentioned research or publication did so using a positive sentiment. With regard to relative citation rates; 17% of CredPubs were in the lower quartile, 59.1% of CredPubs were in the interquartile range, and 23.5% were in the upper quartile compared to control articles.

**Conclusions and Clinical Importance:**

A majority of SAIM Diplomates favored the retention of the publication requirement in some form. CredPubs were cited at rates similar to control articles.

**Abbreviation**
ACVIM: American College of Veterinary Internal Medicine
CredPubs: publications submitted for credentials
SAIM: Small Animal Internal Medicine

## 1 INTRODUCTION

The American College of Veterinary Internal Medicine (ACVIM) was officially recognized by the American Veterinary Medical Association in 1973. The ACVIM was founded by clinician scientists who recognized the need for increased postgraduate training in the basic sciences.^1^ Although there have been some changes to the official mission statement of the ACVIM over the decades, the core components remain the same: *to enhance animal and human health by advancing veterinary internal medicine through training, education, and discovery*.^2^ As part of this mission, the ACVIM enacted a requirement that candidates must publish a manuscript in the peer‐reviewed literature as part of the process of achieving board certification. The ACVIM has evolved to include 5 specialties.^2^ The requirements for board‐certification among these specialties are not homogeneous, and 3 of the 5 specialties currently require a publication as part of the credentialing process. The stated purpose of this publication requirement has been poorly documented and recorded over the years, particularly for Small Animal Internal Medicine (SAIM). At the time of initiation of this study, neither the general information guide for SAIM candidates and mentors, nor the SAIM by‐laws, included a statement of the purpose of the publication requirement.^2^

Without a clearly stated purpose for the publication requirement, institutions and individuals have relied on institutional memory and conjecture about why the requirement is in place. More recently, there have been several online debates about the purpose(s) and merit(s), or lack thereof, of the publication requirement.^3^ It is unclear whether or not these debates represent the opinions of the all SAIM Diplomates. Thus, the purpose of this study was to investigate SAIM specialist's perceptions of, and experiences with, the publication requirement for board certification. Their perceptions were evaluated directly via an electronic survey and indirectly by evaluating practice websites. As a measure of their relative impact, a secondary objective was to compare the frequency with which publications submitted for credentialing purposes (CredPubs) were cited compared to other articles in the same journals during the same time period.

## 2 MATERIALS AND METHODS

A mixed methods design utilizing a combination of survey research, content review of practitioners' websites, and bibliometric/citation analysis was employed in this study.

### 2.1 Survey instrumentation and sample frame

The research team constructed a survey based on the desire to investigate factors such as perceived purpose, value, and importance of the publication requirement; perceived quality, benefits, and costs associated with the research product; and perspectives regarding whether the publication requirement should be retained, modified, or eliminated. The instrument consisted of 12 substantive items (11 quantitative and 1 open‐response) and 5 demographic items (Data S1). Questions were formulated using the best practices of survey construction.^4^

A list of e‐mail addresses for the population SAIM Diplomates (N = 1241) was provided by the ACVIM in August 2016. E‐mail addresses were uploaded to the Qualtrics survey software (Provo, Utah) by the research team, and the survey was administered electronically during a 3‐week window between August and September 2016. The survey consisted of 3 waves: 1 initial invitation and 2 reminders. Permission to conduct the study was granted by the Institutional Research Board of the North Carolina State University (Protocol #9123).

### 2.2 Survey data analysis

To obtain a sample of Diplomates within a 3.5% margin‐of‐error (95% confidence interval), a level of precision comparable to national public opinion polls, we sought to attain a minimum sample size of n = 400. Quantitative data analysis consisted primarily of calculating descriptive statistics. College‐wide (SAIM) demographic data for employment and sex were provided by the ACVIM. Our demographic survey data were compared to specialty‐wide data using a chi‐squared analysis. ACVIM reported that 931 Diplomates are female and 449 were male. Employment categories used by the ACVIM included academia (n = 316), general practice (n = 44), government (n = 12), industry (n = 71), mobile (n = 35), private practice (n = 822), other (n = 46), and unknown (n = 35). Since these categories did not exactly match the categories in the survey, the following categories from the ACVIM reported data were combined for comparisons. General practice, mobile and private practice were combined into a single category: private practice (n = 901). Government, other, and unknown were combined into a single category: other (n = 93). ACVIM reported data were compared to the results of Q16 (area of practice). From this question, the following categories used by the authors in the survey, private practice associate and private practice owner, were combined into a single category: Private practice (n = 270). We sought to determine how the underrepresentativeness/overrepresentativeness of private practitioners and academicians might affect how participants responded to [Q16]. With the procedure described in Royal, we calculated post‐stratification weights to statistically adjust the influence of underrepresentation of private practitioners and overrepresentation of academicians in our analyses. Qualitative data analysis consisted primarily of extracting themes using grounded theory, a methodological approach for analyzing text data.^5^, ^6^

### 2.3 Evaluation of practice websites for references to publication or research

SAIM practice websites were evaluated to determine how frequently SAIM Diplomates acknowledged or advertised research as part of their background and skills (Data S2). The ACVIM Find, a specialist function, was used to identify SAIM Diplomates and their associated websites. If an associated website was not listed, the Diplomate's name with or without additional terms (eg, DVM, ACVIM, SAIM, Internal Medicine, etc.) was entered into a search engine (http://google.com). Academic, government, and industry websites were excluded. Each private practice website was reviewed to determine how many SAIM Diplomates were employed at each practice and whether or not there was a mention of their specialists' publication record or involvement in research. Data were coded into 1 of the 2 categories: (1) websites that mention specialists' previous involvement in publication or research efforts and (2) websites that did not mention specialists' previous involvement in publication or research efforts. All website searches were conducted between July 1, 2016, and September 1, 2016. Two members of the team conducted a sentiment analysis judging whether the comments were presented in a positive or negative nature.^7^

### 2.4 Bibliometric and citation analysis

CredPub were compared to a control group of other publications that appeared in the same journal during the same publication year (Data S3). Article types that would not be acceptable for credentialing (eg, “what's your diagnosis?,” “ECG of the month,” Editorials, Commentaries, etc.) were excluded from the control group. Most Diplomate CredPubs (n = 294) were identified using an electronic database curated by the ACVIM. Additional CredPubs not found in the database were captured by following up with Diplomates individually to obtain the CredPub citation (n = 82). No attempts were made to link CredPubs with survey respondents. All searches for the control group were conducted between (July 17, 2017, and 4 November, 2017). Citations were identified using an electronic database (Web of Science). Each CredPub was compared to similar articles published in the same journal during the same publication year (control articles). Each CredPub was then assigned to a category based on the number of times it was cited compared to control articles published in the same journal during the same publication year: lower quartile (least cited), interquartile range (middle 50%), or upper quartile (most cited).

## 3 RESULTS

### 3.1 Demographics

Five hundred six individuals of the 1241 surveyed (40.8%) responded to at least 1 question. Not every individual responded to every question, so the total number of responses varied between questions. A demographic summary of survey respondents is presented in Table 1. The proportion of female and male Diplomates that responded to the survey was not significantly different from the overall proportion of female and male Diplomates in the specialty (*P* = .19). Five hundred one individuals responded to the question “area of practice,” whereas 455 individuals responded to the question “type of practice” so data from the “area of practice” question was used for comparisons to the employment data provided by the ACVIM. The proportion of survey respondents from private practice (private practice associates and private practice owners) (53.8%) was lower than the proportion of Diplomates in private practice as reported by the ACVIM (general practice, mobile and private practice) (65.2%) (*P* < .0001). The proportion of survey respondents from academia (30.9%) was higher than the proportion of Diplomates reported by the ACVIM (22.9%) (*P* = .0005). The proportion of survey respondents from industry (7.2%) was not different from the proportion of Diplomates reported by the ACVIM (5.1%) (*P* = .1), and the proportion of survey respondents from other categories (8.0%) was not different from the proportion of Diplomates reported by the ACVIM (government, other, and unknown) (6.7%) (*P* = .4).

**Table 1:** Breakdown of self‐identified demographic characteristics in response to survey

Variable	Count (cumulative percentage)
Sex	
Male	178 (35.74)
Female	320 (64.26)
Race/ethnicity	
White	465 (94.51)
Hispanic or Latino	11 (2.24)
Other	16 (3.25)
Type of practice	
Private practice	251 (55.16)
Academia	157 (34.51)
Industry	41 (9.01)
International	6 (1.32)
Area of practice	
Private practice associate	193 (38.52)
Academia	155 (30.94)
Private practice owner	77 (15.37)
Industry/corporate	36 (7.19)
Other	40 (7.98)
Type of residency	
3year residency program with no graduate degree	217 (43.23)
2‐year residency program with no graduate degree	105 (20.92)
Combined residency/PhD	33 (6.57)
Combined residency/MS	130 (25.90)
Other	17 (3.39)

#### 3.1.1 Perception of the purpose of the publication requirement

In response to the question, “What do you believe is the purpose of the SAIM ACVIM publication requirement? (check all that apply)”, the most common purposes selected included “Understand the scientific process” (n = 467, 92.48%), “Learn scientific writing skills” (n = 385, 76.24%), “Nurture a lifelong interest in continuing to contribute to the profession through research and publication” (n = 270, 53.47%), “Make a 1‐time contribution to the discovery mission of the ACVIM” (n = 220, 43.56%), and “Prepare candidates for their chosen career path” (n = 128, 25.38%).

When asked to rank these purposes in order of relative importance, the majority of respondents (n = 276, 58.97%) ranked “Understand the scientific process” as the most important reason for the publication requirement. “Learn scientific writing skills” was ranked second (n = 63, 13.02%), “Nurture a lifelong interest in continuing to contribute to the profession through research and publication” was ranked third (n = 55, 11.83%), “Make a 1‐time contribution to the discovery mission of the ACVIM” was ranked fourth (n = 51, 10.56%), and “Prepare candidates for their chosen career path” was ranked last (n = 36, 7.21%).

#### 3.1.2 Perception of scientific/clinical value

Generally speaking, Diplomates reported the CredPub requirement as valuable with 70 (13.83%) Diplomates rating the requirement “extremely valuable,” 171 (33.79%) rating the requirement “quite valuable,” and 235 (46.44%) rating the requirement “somewhat valuable.” A small minority of Diplomates (n = 30, 5.93%) indicated the requirement was “not valuable.” When asked how Diplomates felt about the scientific/clinical value of CredPubs compared to similar publications in the field of SAIM, 248 (49.11%) indicated they were “of equal value,” 105 (20.79%) indicated they were “of lesser value,” and 15 (2.97%) indicated they were “of greater value.” A number of Diplomates (n = 137, 27.13%) reported they were “unable to judge.” When asked specifically about the CredPub they submitted for their own credentials, the majority of respondents felt they were either “of great value” (n = 57, 11.42%) or “of some value” (n = 323, 64.7%) to the advancement of knowledge in the field. A minority of respondents felt their CredPub was “of little value” (n = 107, 21.4%) or “of no value” (n = 12, 2.40%) to the advancement of knowledge in the field.

#### 3.1.3 Perception of benefits and detriments

When asked how CredPubs benefited Diplomates, 85.71% (432) agreed/strongly agreed that it made them a better consumer of research information, 75.40% (n = 380) agreed/strongly agreed that it allowed them to make a meaningful contribution to the field, and 68.12% (n = 344) agreed/strongly agreed that it helped foster future habits of scholarship. Two hundred thirty‐seven (n = 46.84%) respondents agreed/strongly agreed that it helped improve their marketability for employment. When asked what benefit(s) the current CredPubs provides, most Diplomates (n = 428, 86.29%) indicated it helps “promote critical thinking skills,” “promote scientific and medical communication skills” (n = 427, 86.09%), “promote understanding of the current literature” (n = 426, 85.89%), and “contribute to the growth of knowledge in our specialty” (n = 361, 72. 78%). A minority of respondents (n = 159, 32.06%) felt that it “enhances marketability for employment.”

When asked how CredPubs might be detrimental, the majority of Diplomates (n = 213, 64.6%) indicated it could keep otherwise qualified candidates from becoming board‐certified. Other detriments noted by Diplomates included it could take time away from developing clinical skills (n = 134, 40.61%), and it could place a financial burden on training programs (n = 109, 33.03%).

#### 3.1.4 Delay in credentialing process

A minority of Diplomates (n = 112, 22.22%) noted that the publication requirement resulted in a delay in their credentialing process. Reasons for the delay included “Did not finish writing up the study until after finishing residency” (n = 55, 49.55%), “Manuscript was still under review at the time I finished my residency” (n = 45, 40.54%), “Experienced initial rejection(s) from targeted journal(s)” (n = 29, 26.13%), and “Did not complete the study until after finishing residency” (n = 17, 15.32%).

#### 3.1.5 Future of the publication requirement

Diplomates were asked what, if anything, should be done with the current publication requirement. The majority of Diplomates (n = 428, 85.25%) stated the requirement should be retained either with no changes (n = 186, 37.05%) or with clarifications or modifications (n = 242, 48.21%). A minority of respondents (n = 74, 14.7%) felt it should be eliminated. The proportion of responses for each category remains similar when post‐stratification weighting for employment type was applied (Table 2).^5^

**Table 2:** Employment type post‐stratification weighted responses from Diplomates for the question “Do you feel that the current publication requirement should…” (Q9)

Response	Actual count	Actual %	Weighted count	Weighted %
Be retained with no modifications	186	37.1	185	36.5
Be retained but subject to clarifications or modifications	242	48.2	245	48.4
Be eliminated	74	14.7	71	14.1

Diplomates were asked to elaborate on their response. The following themes emerged among Diplomates stating the requirement should remain unchanged: historical precedent with everyone held to the same requirements; specialty will not grow if only those currently in the field contribute; professional development hinges on Diplomates having the skills to appropriately consume research; if eliminated, many veterinarians will not truly understand how the field continues to move forward; it is a rite of passage; research requirement helps some individuals develop an interest/passion in research that can affect how they choose to spend the rest of their careers; without a publication requirement, residents will be tempted to take information at face value (poor consumers of information); and forces residents to become more well‐rounded.

The following themes emerged among Diplomates who stated the requirement should be modified: further clarification of guidelines is warranted; the list/range of acceptable journals should be significantly expanded (should include open‐access journals); various article types in the realm of research should be acceptable (eg, case reports, review articles, etc.); research requirement should be met before candidates are allowed to sit for the examination; Diplomates should have an option to be the second or third author on multiple research papers in lieu of the first author on 1 paper; mechanisms should be in place to ensure residents have appropriate access to mentors and others who can provide oversight of the research process (eg, potential match program involving residents looking for research projects and institutions that can provide data); Diplomates should have multiple pathways to fulfill the scientific requirement because each publishing experience is different with respect to reviewers, the nature of the topic studied, and so on (1‐size‐fits‐all is not fair and equitable to all); and provisional certification (2 years) should be provided for Diplomates who have completed all other requirements but need additional time for their paper to be accepted.

The following themes emerged among Diplomates who noted the publication requirement should be eliminated: Many deserving candidates do not obtain board certification because of a hurdle with arguably less importance/relevance; project should be required instead of a peer‐reviewed publication; most Diplomates work in private practice so the research requirement is less relevant than other criteria; it is an outdated requirement from a time in which most residents pursued a career in academia upon completion of residency; some candidates struggling to create a publishable paper might misrepresent data for personal gain and negatively impact the field; the skills assessed by the publication requirement (eg, critical thinking, scientific writing, data analysis and interpretation, etc.) can be assessed locally by mentors and advisory committees in training programs; other ACVIM boards do not require a publication (uniformity); there are other ways to accomplish the benefits of publishing an article; residents at academic institutions have a significant advantage over residents in private practice (fairness issues); publication requirement is unlikely to make one a better internist; unfair process for determining which articles are acceptable and which are not; residents with access to more resources and collaborators can have others do most of the work thereby mitigating any supposed benefits from the project; and completing a residency is not equivalent to completing a PhD or Master's program in which evidence of scholarship is required.

#### 3.1.6 Open‐ended responses

Open‐ended responses (with any potentially identifying information redacted) are included as Supporting Information Data S1.

#### 3.1.7 Bibliometric/citation analysis findings

The CredPubs for 376 SAIM Diplomates were definitively identified. Forty‐eight were excluded because comparative citation data from similar articles in the same journals during the same publication year could not be reliably obtained. This resulted in 328 CredPubs available for comparison. All CredPubs were published between 1980 and 2017 and published in 44 unique journals (See Data S4). The most common journals included *Journal of Veterinary Internal Medicine* (n = 74), *Journal of the American Animal Hospital Association* (n = 47), *Journal of the American Veterinary Medical Association* (n = 39), *American Journal of Veterinary Research* (n = 32), *Journal of Feline Medicine and Surgery* (n = 27), *Canadian Veterinary Journal* (n = 26), and *Journal of Veterinary Emergency and Critical Care* (n = 18). The remaining journals all had fewer than 10 articles each. Compared to control articles in the same journals during the same years (n = 29 904), 17.4% (n = 57) of these CredPubs were in the lower quartile with regard to the number of times they were cited; 59.1% (n = 194) of CredPubs were in the interquartile range (middle 50%) and 23.5% (n = 77) of CredPubs were in the upper quartile with respect to the number of times they were cited.

#### 3.1.8 Evaluation of practice websites

A total of 725 SAIM Diplomates were identified via 428 unique practice websites. Of these Diplomates, 328 (42.5%) worked at practices that mentioned their specialists' publication record or their involvement in research. Of the 428 practice websites, 167 (39%) mentioned their specialists' publication records or involvement in research. No website mentioned publications or research in a negative way.

## 4 DISCUSSION

This study was performed to investigate the perceptions of a broad representation of SAIM Diplomates regarding the requirement to publish a manuscript in a peer‐reviewed journal in order to become board‐certified by the ACVIM in the specialty of SAIM. Several important themes were identified from the participants' responses. These include (1) Diplomates' perceptions regarding why the publication requirement exists, (2) perception of the benefits and detriments of the requirement, (3) subjective and objective assessments of the clinical/scientific value, and (4) whether or not Diplomates felt the requirement should be maintained or discontinued.

There was broad agreement among respondents that the most important purpose of the requirement is helping candidates understand the scientific process. Other reasons selected that had majority support included learning scientific writing skills and nurturing a lifelong interest in continuing to contribute to the profession through research and publication. Slightly less than half of the respondents (43.56%) noted the importance of making a 1‐time contribution to the discovery mission of the ACVIM. Two of the reasons, understanding the scientific process and learning scientific writing skills, seem to more directly benefit the individual. Two reasons, nurturing a lifelong interest in research and publication and making a 1‐time contribution to the discovery mission, seem to be oriented more toward benefiting the profession. The specialty of SAIM has recently expanded and clarified the purpose of the publication requirement in the Certification Manual.^8^ The most current version (2019‐2020) states: *The purpose of the publication requirement in SAIM is to ensure that residents develop adequate skills in written scientific medical communication. In particular that residents display an ability to organize scientific data, communicate these data in writing accurately, and that they are capable of discussing scientific findings in a way that promotes the generation and dissemination of knowledge that advances animal and human health. This goal is frequently achieved through education, discovery and contributing to scientific medical literature*. “Helping candidate understand the scientific process” only appears to be addressed implicitly.

A large majority of respondents felt that the publication requirement had benefits including making them better consumers of research, fostering future habits of scholarship and allowing them to make a valuable contribution to the field. The biggest detriment selected (65%) was that it kept otherwise qualified candidates from becoming board‐certified. Although they were not selected by a majority of respondents, there were concerns that the publication requirement could take time away from clinical training or could place a financial burden on training programs.

When asked about the scientific/clinical value of the CredPubs, only a small minority of individuals (6%) stated they felt that in general the CredPubs were not valuable. Among the individuals that felt they were able to compare the value of the CredPubs compared to similar publications (n = 368), the majority felt they were of equal or greater value (71.5%: n = 263), but a substantial number felt they were of lesser value (28.5%: n = 105). This assessment generally mirrored individuals' perceptions regarding their own CredPub, where 23.8% of respondents felt their own was of little or no value to the advancement of knowledge. With the use of citation metrics as an objective measure of value, CredPubs were cited at a rate that was similar to articles published in the same journals during the same years. Not surprisingly, some CredPubs were cited more frequently than their peers, whereas some were cited less frequently. The majority of CredPubs were cited with a frequency similar to articles in the same journals during the same years. A survey of practice websites suggested that many practices that employed SAIM Diplomates (39%) mentioned their specialists' publications and involvement in research in a positive light, suggesting research/publication was considered potentially valuable to them or their clients.

A large majority of respondents (85.3%) felt that the publication requirement should be retained, either as is (37.1%) or with clarifications/modifications (48.2%), whereas a minority (14.7%) felt it should be eliminated. Of those that felt the requirement should remain unchanged, common themes for retention without change included uniformity/precedent and contribution/growth of information in the specialty. The largest number of respondents (48.2%) felt that there should be clarifications or modifications to the requirement. The responses from this group were more varied and included changes in process, content, and credentialing. These ranged from increasing the number of acceptable journals, different types of acceptable publications (case reports, review articles, etc.), accepting contributing authorship, relinking publication and the specialty examination, insuring access to qualified mentors with appropriate resources, and the bestowment of provisional certificates until the publication requirement is met. Of the minority that felt the publication requirement should be eliminated, the themes focused on the relevance to the career path of most Diplomates (private specialty practice versus academia), alternative process to achieve perceived goals, and fairness in the resources to perform (mentorship and facilities) research or fairness during the credentialing process.

The limitations of this study include participation bias and the inability to assess citation metrics for all CredPubs. Academic respondents were overrepresented compared to employment data from the ACVIM, and private practice Diplomates were underrepresented. Despite a participation bias, the overall conclusions regarding what Diplomates thought should be done with the publication requirement did not appear to be affected. This study was also only administered to Diplomates, which means all participants ultimately completed their publication requirement. Thus, it is possible the sample frame surveyed in this study (Diplomates) might offer perspectives that would systematically differ from persons who failed to become board‐certified. Only limited personal demographic data were surveyed (sex and race). Differences in responses between groups with varying personal demographic data were not investigated.

In summary, the majority of Diplomates that responded to this survey expressed that the publication requirement provided benefits to both them as individuals as well as the profession in general and should be retained either as is or with clarification/modification. There may be an underestimation of the scientific/clinical value of the CredPubs among Diplomates which may be related to their personal experience with their own publication. It is important to state that a plurality of respondents (48.2%) felt that the requirement should be retained but subject to additional clarifications or modifications. Therefore, changes to the requirement should be considered while maintaining alignment with the mission of the ACVIM. Importantly, any future changes to policy should maintain a thorough searchable record so that future stakeholders can attempt to understand the rationales (both pros and cons) for all eliminations and additions.

## CONFLICT OF INTEREST DECLARATION

A.J.B., K.L., J.G. are Diplomates of the ACVIM (SAIM) and are involved in resident training. A.J.B. is currently serving as a member of the SAIM credentials committee. A.J.B., K.L., J.G. provide continuing education for veterinarians and their talks may be sponsored by industry. A.J.B., K.L., J.G. have served on advisory panels/provided consulting for industry.

## OFF‐LABEL ANTIMICROBIAL DECLARATION

Authors declare no off‐label use of antimicrobials.

## INSTITUTIONAL ANIMAL CARE AND USE COMMITTEE (IACUC) OR OTHER APPROVAL DECLARATION

Authors declare no IACUC or other approval was needed.

## HUMAN ETHICS APPROVAL DECLARATION

North Carolina State University Protocol #9123.

## Supporting information


**Data S1**

**Data S2**

**Data S3**

**Data S4**
