# Supplementary material for: Perceptions and attitudes of Small Animal Internal Medicine specialists toward the publication requirement for board certification
Source: J Vet Intern Med. 2020 Feb 7;34(2):574–80. doi: 10.1111/jvim.15717 (PMC7096663; doi:10.1111/jvim.15717)
Supplement: Supplementary file 1 — Data S1 [file JVIM-34-574-s001.pdf]

ACVIM Diplomate Survey

September 21st 2016, 9:14 am EDT

Q1 - What do you believe is the purpose of the SAIM ACVIM publication requirement? (Check all that apply)

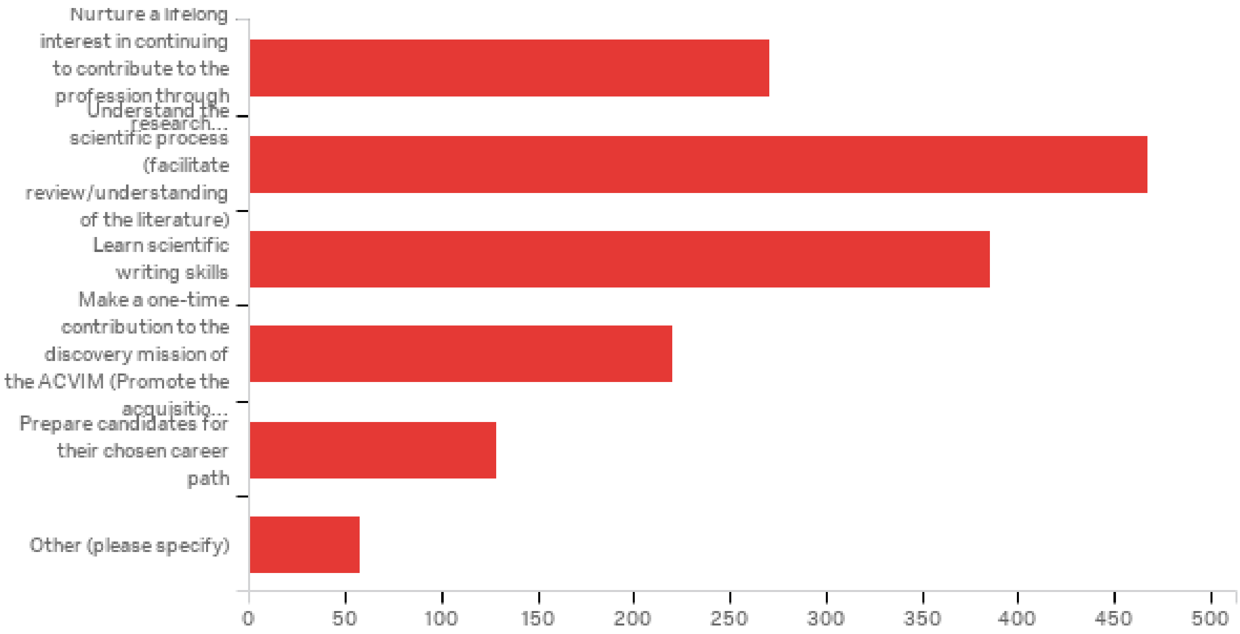

Answer	%	Count
Understand the scientific process (facilitate review/understanding of the literature)	92.48%	467
Prepare candidates for their chosen career path	25.35%	128
Other (please specify)	11.29%	57
Nurture a lifelong interest in continuing to contribute to the profession through research and publication	53.47%	270
Make a one-time contribution to the discovery mission of the ACVIM (Promote the acquisition of new information relevant to the specialties of the ACVIM for the benefit of improved animal health)	43.56%	220
Learn scientific writing skills	76.24%	385
Total	100%	505

Q1_6_TEXT - Other (please specify)

Other (please specify)
Provide residents with time and the means to explore resources outside of their current facility including access to clinicians or specialists that they may not have, labs, equipment ect.)
Act as a gateway/barrier to diplomate status (everyone else did it so we had to as well)
Improve overall communication skills.
Understand the publication process
Writing my paper really allowed me to understand what constitutes a good scientific study vs. a poorly designed one. For this reason alone, I think the requirement should stand.
The above first three choices are what I hope the requirement is supposed to help accomplish.
Allow candidates to be exposed to the process of research and scientific writing which may promote an interest in these aspects of being an ACVIM diplomate (vs clinical practice), thus providing the candidate with a possible alternate career path that may not have been considered. Also to allow the college to maintain a more vibrant interest in research among all diplomates by supporting this type of work among its potentially newest members.
To contribute to the veterinary profession and medical science field in general
The purpose would be all of the above however, I do not believe the requirement fulfills any of the above for numerous reasons
a hoop to jump through and an attempt to increase veterinary literature that often does nothing to augment the body of literature
Ensures that diplomates are able to understand the literature.
1. Become more critical readers of the scientific (veterinary) literature. 2. Ensures resident advisors are remaining active in the discovery mission of the ACVIM. Note: Career path preparation value does depend on the career chosen (academic/industry>private practice)
The publication process I believe is important as it causes the candidate to critically contemplate how information is acquired, presented, and then assessed through review. Giving the candidate a glimpse at the certainty that all information comes with a motive, can be purely objective or subject to bias, and that all information needs to be considered critically (even after publication) and not simply accepted as truth. I believe a failed publication attempt is as important a lesson as a successful attempt. I do not think the candidate should be excluded from certification because of the lack of publication success, lack of effort, yes...
Frankly, I do not understand how even the checked box positively impacts the candidate
Learn how to critically assess a publication to be able to appreciate the strengths and weaknesses of a study (especially if it is a case review or clinical trial).
It reinforces the quality and applicability of resident projects. It eliminates nonsensical projects that would have no hope of ever being published being done as a means to satisfy another requirement.
Teach residents how to research into a topic in depth more than what is generally needed for boards etc.
a hoop to jump through
Hazing! Just kidding.... It is an important hurdle that allows one to understand the literature process and more critically evaluate other's research. I found it incredibly helpful.
Develop of critical thinking and publication assessment skills- for themselves and to be able to provide this service for general practitioners.
I would say make 'at least' one contribution to acquisition of new information

The first is a nice to have for sure, but not a goal

I believe that the publication requirement is the final evidence of the successful completion of the research project; i.e. successfully passing the research project examination

Giving candidates the opportunity to be exposed to research, which may help them decide on their future path as an internal medicine specialist. (this is similar to preparing for career path but different in that some candidates may not know they love research until doing it, hence changing their 'chosen' career path).

Give candidates the opportunity to explore research. Some may find they enjoy it and want to pursue in the future.

I believe these are the purposes of the ACVIM but not the outcomes.

Create awareness of the difficulty of doing clinical research and appreciation of the limitations of findings of almost any clinical study

Right of passage

Learn how to develop a project/hypothesis, proper study with study subjects, and bench laboratory skills to appreciate what goes into n original study (more than collecting samples and submitting them).

Learn advanced literature search techniques and acquisition of publications, management of large numbers of articles in available programs for longterm search potential and for organized footnoted papers.

Our profession suffers from the tendency toward inability to discern science from nonsense. This seems to be true most obviously for the many 'band-wagon' treatments recommended by colleagues. There seems to be a trend toward 'junk science'. There seems to be fewer recommendations, particularly in non-refereed journals and in journals in which peer review is of substandard quality, that are backed by solid evidence. Whims get tossed around; data is shuffled so that what is 'trash' appears by screwed stats to be a 'significant trend'. Perhaps the large egress from university based residency programs has contributed to this dilemma. This seems to be particularly true for recommendations based on 'science' coming out of foreign countries. Has anyone been paying attention to the similar problem with predatory journals in human medicine as well as agricultural research? There are a lot of folks with an agenda to publish, and not necessarily for the good of society.

Hurdle in the credentialing process

Develop an appreciation for scientific research by understanding how it is conducted.

Historically I think the publication requirement was instituted- for many of the reasons above- to expose residents to the process of formulating and executing a study and writing it up for publication- Likely a similar thought process to those who espouse the benefits of a " broad liberal arts education"- a "broad scientific education fro a career in veterinary medicine/science"

Serve as an impetus to complete a small scientific study (not for all, but for some at least)

Enables faculty to complete projects via residents.

Prepare a manuscript that demonstrably shows standards of research/case management a specialist should know. Undergoing editing process is a great lesson on how to present a thorough, comprehensive case or manuscript (at least the ACVIM journal was).

Improve understanding of evidence-based medicine

Collaboration with colleagues.

I think it is essential to developing a well rounded specialist. I thought he same things about the case reports.

These are all worthy goals and absolutely necessary for the preparation of successful faculty members. The shortage of people prepared to be clinician-investigators is extreme, and unreasonable expectations probably contribute to the churn of young faculty who become frustrated and leave within a few years of taking faculty positions. However, I don;t think we are being successful by using the publication requirement to accomplish this.

Be a worker bee for their mentor's requirements to publish for tenure and promotion.

Improve written communication skills

By being authors (even once) candidates gain a better ability to critically read literature in the future - a skill they *all* must develop, regardless of career path.

It is part of their training as an Internal Medicine Resident. A SAIM Diplomate should be able to critically read the medical literature, and being taught how to write a scientific article is critical to that learning process.

With the loss of "case reports" we have no way other than a publication requirement to meet the minimum standard of technical writing proficiency that is a must for our college.

I am not sure how effectively the requirement meets any of these goals though! For some candidates it is just another hoop to jump through. For a minority it may them interested in research. Few residents gain enough experience through a program to become an effective PI. In order to do this most people need to do a PhD.

Help academic specialists to obtain notoriety and tenure

Hoop to jump through to achieve board certification

I think this stimulates some candidates to reconsider academics or industry.

Item 4 amended to say make a FIRST contribution to the discovery mission of the ACVIM. Continued contribution should be a goal for every internist. These publications should be well thought-out and hypothesis-driven. They should be more than something done to fulfill a requirement/check off a box. They should be part of a comprehensive training program that does more than teach residents how to do the day-to-day aspects of working up a patient and learning skills like endoscopy and ultrasound. Good internists have continually inquiring minds and want to push the borders of medical knowledge forward as they pursue the practice of medicine. This is not limited to those in academia or pure research positions. A good internist continually asks questions and seeks answers through research, and must know how to go about this. The publication requirement is a part of the process of teaching that and instilling a lifelong passion for furthering medical knowledge and providing the best possible evidence based care for patients.

As writing a paper does not teach candidates any of the above choices, one can only conclude that it has NO PURPOSE except to generate poor publications for academic progress.

Technically, I would say to "demonstrate" scientific writing skills (vs "learning" scientific writing skills)

Q2 - Please rank-order the importance for each item with '1' representing the most important and '5' representing the least important.

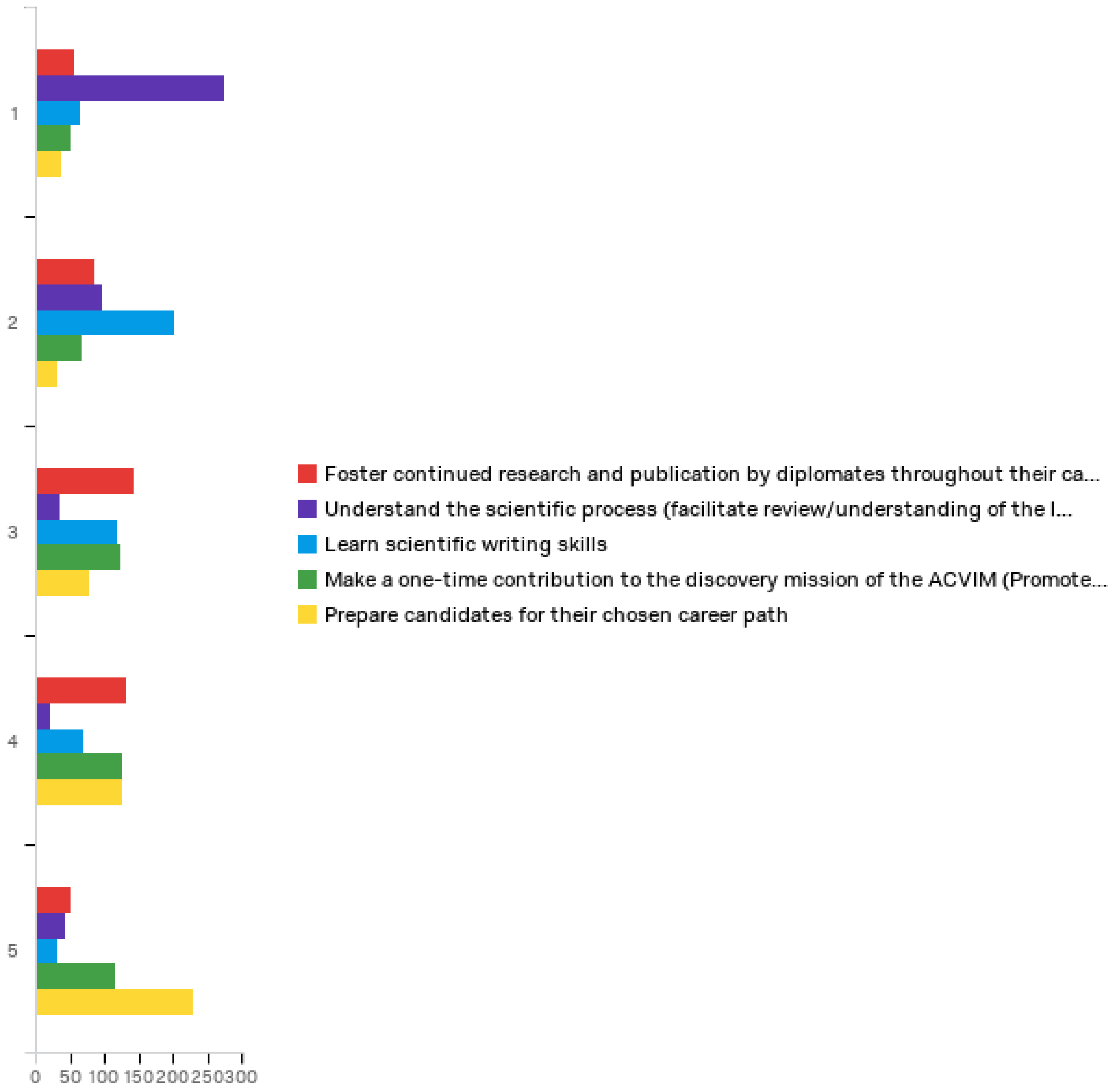

Question	1		2		3		4		5		Total
Foster continued research and publication by diplomates throughout their careers	11.83%	55	18.49%	86	30.54%	142	28.17%	131	10.97%	51	465
Understand the scientific process (facilitate review/understanding of the literature)	58.97%	276	20.30%	95	7.48%	35	4.06%	19	9.19%	43	468
Learn scientific writing skills	13.02%	63	41.53%	201	24.38%	118	14.46%	70	6.61%	32	484
Make a one-time contribution to the discovery mission of the ACVIM (Promote the acquisition of new information relevant to the specialties of the ACVIM for the benefit of improved animal health)	10.56%	51	13.66%	66	25.67%	124	26.09%	126	24.02%	116	483
Prepare candidates for their chosen career path	7.21%	36	6.21%	31	15.43%	77	25.05%	125	46.09%	230	499

Q3 - Regarding the following, completion of the ACVIM publication requirement allowed me to:

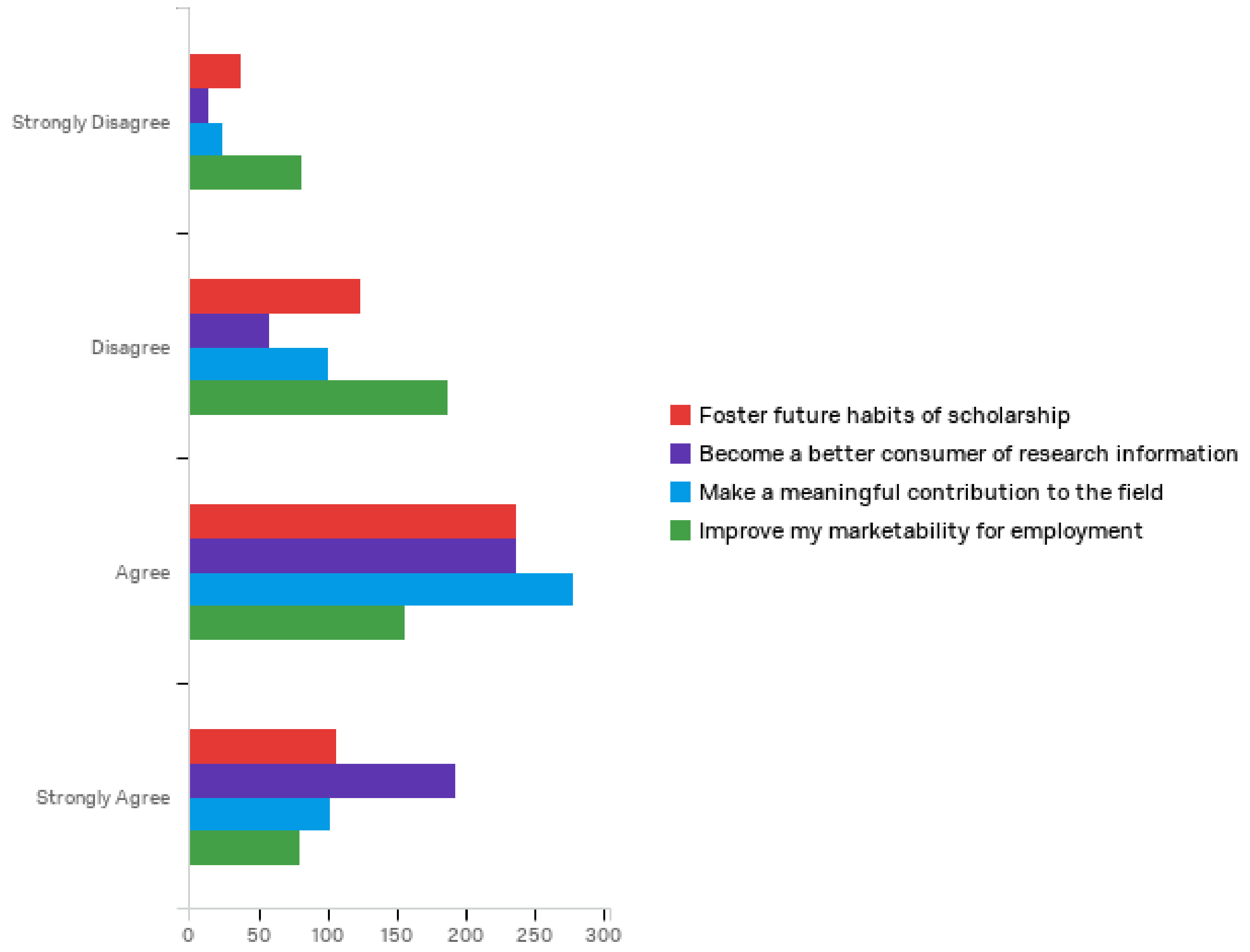

Question	Strongly Disagree		Disagree		Agree		Strongly Agree		Total
Foster future habits of scholarship	7.33%	37	24.55%	124	46.93%	237	21.19%	107	505
Become a better consumer of research information	2.78%	14	11.51%	58	47.22%	238	38.49%	194	504
Make a meaningful contribution to the field	4.76%	24	19.84%	100	55.16%	278	20.24%	102	504
Improve my marketability for employment	16.21%	82	36.96%	187	31.03%	157	15.81%	80	506

Q4 - Regarding their scientific/clinical value, I believe that resident publications submitted for credentialing purposes are:

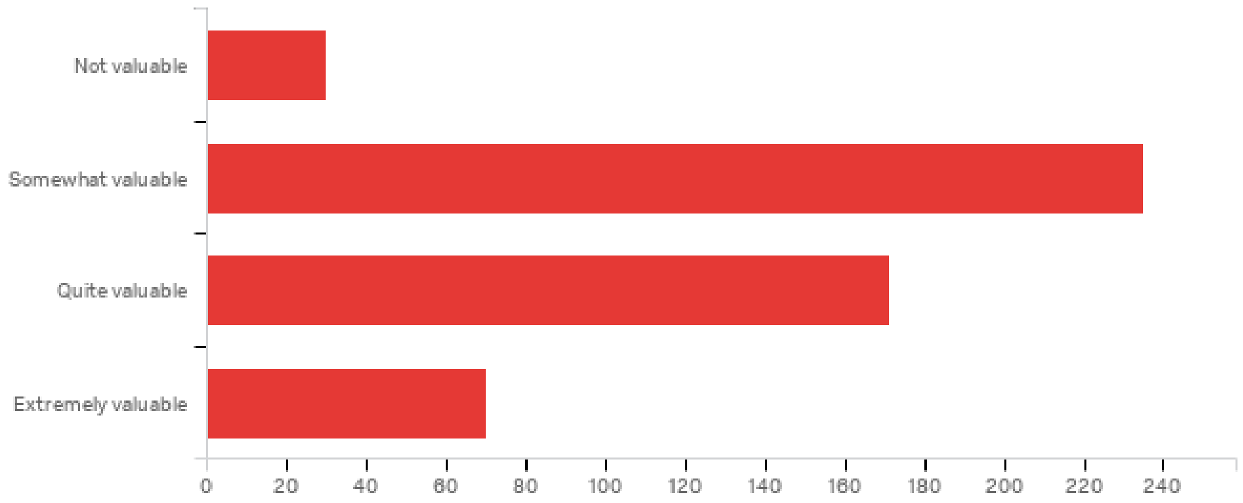

Answer	%	Count
Not valuable	5.93%	30
Somewhat valuable	46.44%	235
Quite valuable	33.79%	171
Extremely valuable	13.83%	70
Total	100%	506

Q5 - Compared to similar publications (e.g., case report vs. case report, prospective controlled trial vs. prospective controlled trial) in the field of small animal internal medicine that were NOT submitted for credentialing purposes, the scientific/clinical value of resident publications are:

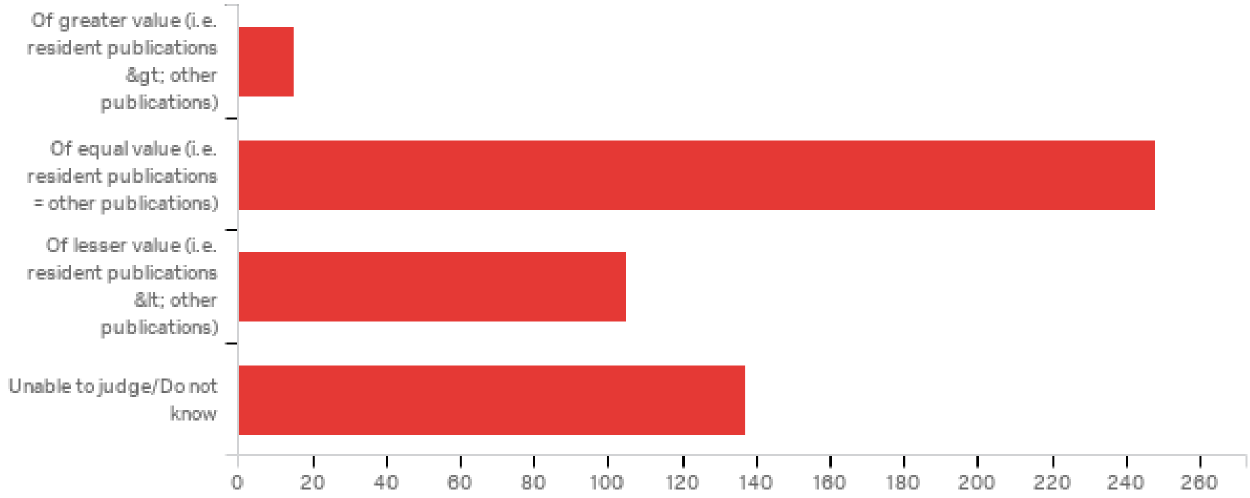

Answer	%	Count
Of greater value (i.e. resident publications > other publications)	2.97%	15
Of equal value (i.e. resident publications = other publications)	49.11%	248
Of lesser value (i.e. resident publications < other publications)	20.79%	105
Unable to judge/Do not know	27.13%	137
Total	100%	505

Q6 - Please elaborate on your response to the previous item, if desired.

Please elaborate on your response to the previous item, if desired.
Resident projects are frequently based on very good ideas but often small, preliminary or underpowered due to the associated time constraints, which is very frustrating as a reader/consumer when those projects could have been completed to a higher standard.
The positive aspect of a resident publication is (in general) that they have a wider range of resources if they are in academia. Benefits of other publications is experience.
this is not without exception, but generally speaking, I do not perceive a significant difference in resident-run vs. faculty-run publications, likely because faculty are so closely involved in resident projects.
It is important that the quality is maintained by ensuring only appropriate peer reviewed journals are acceptable and minimising case reports.
in GENERAL, resident publications are of greater value to the resident than to scientific/clinical value. i think the publication is still of GREAT value to the resident. there are certainly some resident publications that are of exceptional value to all.
Our resident projects were small, and I felt that 3 years didn't provide enough time to 1) brainstorm, 2) acquire funding, 3) collect data, 4) write, and then 5) publish a strong study that contributed significantly to the field of small animal internal medicine. I felt that the process of it was valuable, though.
Indicative of active research and investigation portion of the residency in the chosen specialty
I'm not sure that I fully understand the qualifiers, however if interpreted with the idea that a resident publication is less of that then a seasoned clinician, I dont believe that I read publications thinking oh this is from a resident so its less important. I assume that they have a seasoned clinician guiding them through the process. I wish that I had had more guidance during my residency when it came to literature review and publication.
I believe that the time constraints for resident research projects limit the quality of publications resulting from the credentialing process. I agree that there are rare exceptions to this rule, but residents who are not engaged in a graduate program have a limited amount of time to devote to discovery and research.
As with much of of our scientific literature, they are underpowered and resident projects are often short term, underpowered projects (due to time constraints of getting case numbers, etc) that may result in the general acceptance by the veterinary community and change in practice. Rather than carefully performed, reasonably powered larger, multi-institutional studies that allow for meaningful change in practice. The community including specialist often times will quote underpowered studies to further their viewpoint and change practice rather than to carefully review the previous literature and/or clinical experience. Additionally, reliance on clinical experience is fraught with the the added stigma of not being evidence based; is anything we do really evidence based? Even when we have data? Meta-analysis reviews of less well powered, non-randomized or pilot studies in people allow for a culmination of the data and a more comprehensive understanding of the "rationale" approach to the problem at hand. These allow for the identification of misinformation presented in smaller studies to be highlighted and not universally adapted by the community; leading to a potential reduction in patient morbidity, unnecessary cost and potentially mortality. Wouldn't it be better if our residents contributed to larger projects as part of a larger group (ideally multi-institutional) that could be adequately powered and over appropriate time periods (10 year studies, etc) contribute to changing/evolving practice. Is turning out a publication for a requirement really contributing to the profession?
Depends on the publication in question. In reality, it is not likely that in a 2-3 year window a resident will develop an idea for a study, get the funding, accomplish the study, evaluate the results, write it up, and get it accepted for publication. So, they usually end up with a case report, a retrospective or review paper, or piggyback on a generous colleague's work.

In general, I have not paid attention to whether a publication was written by an individual in their residency or not. In cases I can think of where I know a resident wrote a publication, the value of it depends mostly on the mentorship.

I don't believe the value of the information is dependent upon who is submitting the information.

The value of published prospective studies really depends on a candidates chosen career path. If the candidate intends to go into private practice that does not have a residency program their is little value to no value to their prospective employer or clients.

It is hard to answer the question as I do not always know when I read an article if it was a resident project or not.

As with all publications, some are fantastic and some are less so. Residents often provide a "new" perspective and can be quite creative in the study design process or with interpretation of findings/results. (Sometimes in a good way and sometimes in a not-so-good way.)

Having residents think through the process of writing and publishing information although difficult should remain a part of the process. I think the mentors really need to step back from having the resident publish a paper just to have their name on it and for their promotion if the project is too difficult for the resident to complete. Realizing that this is difficult for some, it would be beneficial if ACVIM could make some suggestions of projects and publications that have been better for residents to complete the process on time. I think that the case reports are best but some feel that this does not add much to the literature. It would be nice to go back and compare the type of publications vs time to complete the credentialing process and outcome.

I feel like publishing a paper for credentials is a right if passage. I have never appreciated any value of my publication but feel like it is a process that each future diplomats must learn to undertake. Basically, I hated the process but feel like it should be done.

When I read literature, I don't try to suss out if it was a "resident paper" vs a "non-resident paper." That being said, I do have a concern that resident publications may be of lesser value since they by necessity have to be more limited in scope.

I don't think I could say which publications were for credentials and which weren't. Residents publishing more than what is needed for credentials may also be a skewed population with a drive for research.

Widely dependent upon subject matter; each publication different

I am curious as to your agenda with this "research" survey.

I worry that papers are being submitted for publication to fulfill requirements and are not always submitted to the most ideal journals or they are not pushed to be of the best quality.

Learning how to analyse and report individual cases can be as enlightening to the individual and reader as a clinical trial

I think it can be stated that there are poorly written publications in both categories AND excellent written and designed publications in both categories. I do NOT think it is a forgone conclusion that all resident publications are bad and all non-resident publications are good.

Depends on mentorship; some will be less equal if not well mentored some may be equal to the caliber of the independent studies of the diplomates mentoring, some case reports are stronger than those of some GPs

That comparison will actually be a good research project.

I think it is too variable to comment and the research fields too diverse.

All new.knowledge is valuable

The value is variable, depending on the nature of the resident project and input from the research mentor. There are some excellent and valuable publications with residents as first author. However, some resident publications add little scientific/clinical value to the literature.

Some of the resident research is the focus of not only the resident but her/his supervisors or mentors. This fosters good research. It can lead to more thorough research in the future. Yes resident projects are limited by the

duration of the residency and the need to be published quickly, but I believe these endeavors start as pilot studies and lead to subsequent research.

The wording of the question, specifically the example given (e.g., case report vs. case report, prospective controlled trial vs. prospective controlled trial), is too confusing so I cannot interpret what is being asked. Generally, I think resident-authored papers are as valuable as other publications but I am not sure if this questions is getting at the fact that case reports are excluded from 'allowable' resident publications.

I personally do feel that there are resident publications that are acceptable and of good quality but I also feel a large number of resident publications lack in quality and are written merely for the requirement of publication.

Residents publish whatever they can because they have and they may or may not be a contribution to the profession whereas other publications are published because they are felt to be a contribution to the profession.

All literature has the potential to benefit other veterinarians and patients, especially when the pool of submission is as large as possible (i.e., mindset of the individuals is varied) rather than solely from a narrow pool.

In general, lesser. However, there are some great contributions so it's not universal.

In general, because we require residents to complete in essentially a 1 1/2-2 year period what takes the rest of us who do research normally 3-5 years, of course the quality of the work is going to suffer. Unless the senior has previous project already in process, in which case the resident misses out on the opportunity for study design, etc.

Depends on the resident and their training program.

Most residents likely do not put as much effort due to just "wanting to get it done", making prospective or well controlled studies less common.

The ability to successfully publish more reflects the organization and dedication of the resident's mentor than it reflects the residents innate organization skills or abilities. Under residency timeline considerations, aside from novel case reporting, a well constructed resident project may need to be in a planning or organization phase before the resident starts training. The successful publication requirement of all certification requirements, often relies on resources out of the residents control. The requirement for successful publication can have a political flavor and can result in the littering of the database with information of limited or poor quality, except as a "good example of a bad example..."I believe the most important aspect is that the resident complete the process regardless of the success of the effort.

Many of the publications probably are the result of the resident(s) contributing the vast majority of the work, from running any clinical trials to evaluating the data, to writing the paper. The experiences gained from these endeavors are valuable, but generally the quality of the paper is equal to what is produced by senior clinicians. I think what makes a paper less valuable, regardless of resident participation, is when the conclusions state "more research is needed...." While these papers are interesting, they are not very helpful from a clinical standpoint, and so I think if research is going to be done, a definite end point/conclusion should be reached, and in many cases, to do such research goes beyond the time frame of the resident training program.

I do not see an overall benefit to the candidate for the publication requirement. The vast majority of these papers (my opinion) provide rather meaningless and forgetful articles that are not scientific worthy in a critical sense – REDACTED opinion.

Prospective, and to a lesser extent, retrospective resident research is just as valuable as other publications. I do believe that case reports fail to contribute significantly to the body of literature.

Residents require mentorship in writing and go through the peer review process, making the quality similar.

As with much of the Residency training program, it depends on the program and, especially, on the mentors. In a program with active prospective projects in the pipeline a Resident Project and publication can be a valuable addition to the literature. In a program where Residents are just thrown in without much guidance the publication is likely to be less useful. Residents, as fresh minds, may offer a different perspective than their mentors on what elements within a subject are most 'worthy' of pursuing, and may take projects into new, unplanned directions (fortuitous accidents are a staple of research, benchtop and clinical)

All depends on the quality of supervision. Ultimately, that s the journal that his the guardian of the quality of publication in my opinion.

All Diplomates need to be able to understand the effort it takes to create a publication and how they should contribute in any small way to the progress of science.

Some resident papers seem to be written simply for the sake of meeting the requirement, rather than really filling a gap in the scientific literature.

I can only speak for my own Residents, whose work is of similar quality to other publications in the journal in which they publish, which is usually JVIM -- i.e. they are not pumping out garbage just for the purposes of credentialing..

I think it really depends on the paper. There have been some "resident " papers that are foundational in my opinion. Let's be honest, there are also "publish or die" papers that aren't very good either.

First, you keep referring to them as "resident" publications but credentialing papers may have be performed outside of the residency or completed after residency which, due to time commitments during residency, could be a big difference. Second, residence projects vary so greatly in their complexity and depth that there is not one answer to the question.

In general I think most residents are interested in clinical practice rather than research. For residents not interested in research, the publication requirement is something they just want to get through and many pick the easiest projects they can do.

Should be equal quality to other articles in the journals selected by the ACVIM

They could be equally valuable if a good topic was chosen and if the resident was supported well by a mentor/addt'l author; however, many papers were "crappy" and just barely squeaked by the resident author's own admission

Under current guidelines, the "value" of the publication is, to a very large degree, a reflection of the work represented by the publication. One would not argue (I think) with the value of a publication reporting results of an adequately powered randomized, placebo-controlled prospective study, and most would argue (I think) that such a paper is "more valuable" than a case report. Some might argue that a well-done case report has more "value" than publication of results of a poorly powered retrospective study from which useful conclusions can't truly be drawn. Lastly, value is a perception that is best assessed through the eyes of the beholder, which is why (I suspect) you might get responses across the spectrum of options above. How does one judge value for something like this? The question has me thinking that it would be interesting to see, over some period of time, how many resident publications are cited in another paper (would that be a reflection of value?).

I'm in the camp that believes that the residents don't have the time/funding to do a meaningful study and I worry that their time/energy is not best used in pursuing this publication requirement. It seems like a hoop to jump through. I'm not sure how many otherwise-qualified residents are stopped in their quest for board certification by this publication requirement, but why should ANYONE be stopped by the publication requirement, if they're otherwise qualified to be board-certified?

Choices are all opinion-based and lack any objectivity therefore unable to judge.

I do not see the point of this question. It may be clear to you but not to me. What is the difference between case report and case report or prospective controlled trial vs. prospective controlled trial?

Many resident-authored publications appear to have been planned as high-impact studies, designed to answer very relevant clinical questions. However, the publications which eventually make their way into print are oftentimes underpowered, either from failure to prospectively enroll the planned number of animals, or due to marked variability in the retrospective population identified for case series. This is most likely (in my opinion) due to the supervising diplomats being inappropriately optimistic in number of anticipated cases to be enrolled per year when applying for grants that ultimately fund the prospective studies, or because very few resident retrospective case series are multi-institutional collaborative studies.

Some resident clinical studies are better, soe equal, some worse compared to clinical studies from non-residents. In term of scientific (not clinical) value, they are commonly below average.

There is always a big name behind these publications (almost always)

I think that in some cases a resident publication is definitely equal to or better than other publications, but that for some residents it is just a motion to go through and thus the value is less than other publications in which someone is very passionate about the information provided.

Since they must be completed in a few years, and be relatively straight forward, resident research projects are rarely rocket science or ground breaking compared to other research. The days of publishable ACVIM-accepted case reports are almost gone, so it is hard to compare in this area.

It is a means to an end, but in people not predisposed to do research, it is just a hurdle to jump through to get to board certification & in most cases is not necessarily going to add significantly to the greater scientific knowledge base.

Not all projects/publications are quality

Most of what is published are low number studies with dubious scientific value. Case reports are just an exercise and rarely advance scientific literature. ACVIM needs to focus on clinical training. Advanced degrees are for fostering scientific writing skills.

Clearly, there have been a number of very valuable publications that were used for credentialing purposes.

I find it difficult to make a broad statement on the above, ie. some of these are of higher value and some aren't...this depends on a variety of factors. I do think these should be assessed the same way any other publication is, ie. peer reviewed vs. stating they are from a 'resident'.

Forward movement of the veterinary field is hampered by few dedicated researcher hours (e.g. faculty FTE, time or support for specialists to conduct studies and publish, etc) and little financial support. Although almost by definition, most candidate publications do not provide major leaps in knowledge, even well presented case series can add to the knowledge base and advance the field. If veterinary medicine relies on dedicated, fully trained researchers, we will make little progress indeed. The mission of the ACVIM is to enhance animal and human health. Candidate projects and publications do so, in and of themselves. Ideally, they stimulate the candidate to continue to contribute beyond their program.

How would I know? They are not labeled as such in publications

Dependent on the publication. Some are obvious 'resident projects' written for no other purpose than to fulfill a requirement and contribute little. Others are much more valuable.

Residents I foster work with ongoing projects I have in progress during their program. Our residency does not permit time for independent at the bench or otherwise original discovery- considering the varied talents of individuals in those areas. I orchestrate their projects with research materials I have on hand to get them involved with data evaluation and scientific writing. How many have gone on to use those skills - over 38 yrs- maybe 3. So in effect, their projects are my research endeavors- the material is published and they get an experience. It is a lot more work for me. I do not think this is a valuable experience for most destined for specialty practice. Also, when I look at the projects coming out of many REDACTED schools where the clinical programs are not full of cases in sophisticated facilities- I see more esoteric projects that could qualify for some part of a graduate degree. Not possible at our school. Our residents are in such debt from their educational costs that most cannot financially take on a graduate training program for PhD- plus the age factor compared to REDACTED others that fast track students in Vet Med. Finally, as Internists in Human Medicine do not need a publication to be qualified/certified- so I don't understand how we can continue to justify this publication requirement. I could let it go with a smile. If we had a venue for them to publish interesting cases, a one-of kind of case- that might be more appropriate.

Resident reports at the universities are largely driven by the faculty which is a good thing.

I believe it really depends on the project and the publication. Good projects which result in valuable contributions are very useful. Retrospective studies of small cohorts with equivocal results are discouraging for everyone. Perhaps the emphasis here should be placed on the project. This is a big challenge for the supervising team but perhaps getting the basics right will ease the trauma of getting that publication out.....

It obviously varies a lot - some of these publications are great papers and contribute significantly to our knowledge whereas others are unfortunately "alibi publications".

Some resident publications are merely written to fulfill the requirement and do not add much to the existing pool of veterinary literature, whereas others are excellent. It very much depends on the mentors/supervisors and set-up.

My own publication became a hallmark study, and I wouldn't have been involved if not a resident. While some resident publications may not be ground breaking, most of the publications in journals that I read seem to have a resident as first author.

Most projects are thrown together in order to complete in the time allowed during the residency diluting our literature with lesser quality information.

I'm really not sure what you're asking-am I supposed to be differentiating which ones were submitted for credentials and which ones were not? I don't know how I would know that about residents that were not my own …

I believe many people check the block and push out something that may not be very useful in order to meet the requirement. That person is unlikely to be passionate about that topic, whereas some one that is passionate in a particular area is much more likely to produce valuable/meaningful research that will further the profession as a whole.

If I am understanding the question correctly, you are essentially asking if a case report or controlled trial study published for credentialing has the same or different scientific value as ones published for other reasons? If so, I think they are of equal when compared apples to apples.

It really depends on the resident and their interest in research. A resident who really likes research/publications will likely publish a more valuable paper than a resident who lacks a desire to pursue research/publication in their future career and is just trying to get a paper done for credential purposes. I still think residents should have to write a paper (maybe there could be an option for internal review and acceptance as publishable quality vs actual publication) - everyone should go through the process at least once. On another note, publications in veterinary medicine should strive to streamline their information (such as in NEJM) as they contain a remarkable amount of excessive/redundant information.

There are major problems with residency / ACVIM candidate research projects and publications. They are, by nature of the candidates, short term and of limited numbers. The time spans are usually not sufficient to adequately follow-up on patient outcomes, and the numbers are not statistically relevant. Thus, we get to read many publications that are mostly useless and often misleading. This does not advance veterinary medicine. More useful to candidates, the majority of whom now enter private specialty practice rather than academia, is the ability to critically evaluate publications. This is what we, as continually updating specialists spend most of our reading and CE time doing. Understanding basic statistics (such as n=6 is not significant whatever the outcome, and so on). evaluating the choice of patients, sorting out prospective aims from retrospective analysis, etc. Not just reading the abstract or the poster. Understanding actual evidence in our or the medical profession is essential to the practice of good veterinary medicine and the advancement of ACVIM.

Residents are just doing what they can to get published to meet the requirement-these are not always good contributions to the scientific literature. Also ability to do a good paper/project strongly depends on residency program.

I don't understand the previous question

Widely variable value and that is true of all publications so equal value...

This is a loaded question. There is good research on both sides. There is also garbage coming out of both sides. Residents are eager and a lot of the quality of the product they produce is also dependent on the quality of their research mentors. There are many in academia that push out manuscripts just to keep their jobs - often garbage.

I really don't think there is a good way to answer this, and am not sure it is a valuable question. For the most part the journal reviewers should be determining if a project provides new and important information and whether it is ">" or "=" to other publications is not as important as that simple criteria unless we really believe that the "<" research is in some way preventing better research from being published. In most cases, I think resident projects are guided by diplomats anyway and would be done by interns, students, or even the clinicians if the resident did not need a project. In general I think resident case reports are equal to other case reports. I think as a whole,

resident prospective clinical trials are likely to be < non-resident clinical trials (mostly due to the time constraints of a residency), but there are definitely exceptions to this. This does not mean that they are not worthwhile contributions, however. Also, if residents weren't producing them, a lot of them might still be done by the current mentors so there would be a subset of trials that was "<" another subset of trials.

Many residency projects are not adequately set up and mentored by a faculty. Faculty often know too little of subject matter and research tools to pursue a study. frequently there is not sufficient funding and there is time pressure because of the time period of the residency

This is s confusing question based on wording. If research is good...all can benefit from results. If research is done poorly or stats are manipulated to make it appear as if a particular outcome was obtained, then there would be no benefit.

Resident projects are frequently underpowered or small in scope resulting in a less meaningful publication. If resources were pooled and residents worked together on larger scope projects while still meeting a requirement, this may be alleviated.

I think they are of equal value because they also been held to the same high standards of the peer review process

Historically I think more residents were headed towards a career path where they would contribute to tomorrows medicine and a publication requirement was viewed as in keeping with staying in an institutional/academic career path. However, time changes many thing and the vast majority of the residents I train are not vested in making tomorrows medicine or pursuing a career in academia- their sights are firmly set on specialty practice and the medicine of today. They have less innate curiosity and much less drive to design, execute and write up a study for publication to meet the publication requirement that they see as irrelevant to their future goals. The time and energy spent on projects to meet board requirements is not time well spent. Why not drop it as a requirement and those who are interested can pursue optional separate (before or after residency) training in research/scientifc methods- e.g fellowship, PhD etc

Depends on the publication/project, but these can be good projects.

I do not automatically know which publications are resident driven and which were not when I read articles.

When a candidate who does not have research interests is forced to write a research paper, inevitably the result often will be a single case report or review that does not contribute significantly to the scientific literature. Ideally, we should be fostering prospective studies regarding meaningful topics that have sufficient power to draw meaningful conclusions, instead... but, of course, these also are expensive and time-consuming, making them more difficult to complete in the context of a residency.

I don't really understand the question

I do not understand the wording of this question.

I would have to read and compare to answer this question for it to be meaningful. I don't always seek the credentials on authors on subjects beyond my area of interest.

depends; some of the resident projects are better than single case reports published by diplomats. some studies during residencies were great information compared to drug trials in practice. i think they have value.

huge variation in both groups

I am unsure what question is being asked here. Are you asking if case reports are less valuable than retrospectives/prospectives?

The overall value of the publications is quite variable. The quality of the research is affected by time constraints, mentorship, and available research funding. However, many times the studies performed and very simple and do not supply a large amount of information or they are too ambitious and therefore are incomplete or the design is not ideal.

I think it depends. In some cases, it seems obvious that the publication was done as a resident project and does not offer something as valuable as far as the subjective matter, research done (for example, in some retrospective studies, some case reports but not all, more basic prospective studies), but in other cases, the publication may be just as valuable.

The quality of publications in general is highly variable. So too are the resident publications, which in my opinion are not readily characterized as more or less valuable. It is easier to say that publications based on study designs intended to yield high-level evidence are inherently more valuable.

My assumption is that both would have gone through an external refereed review ==> makes them equal

The publications are always done with a faculty member, often as part of ongoing research they are doing. Often the resident is helping the faculty member to complete their projects.

Unfortunately, few of the projects attempt to answer important clinical questions and are often chosen for their likelihood of small incremental success

I don't entirely understand this question.

Submitting a publication that entails original research and consideration of the process entailed in furthering our knowledge in veterinary medicine should be important to all practicing clinicians. I believe knowledge from publication worthy clinical cases is equally important. However, learning the rigors of proper experimental design and how to ask an answerable research question is lost in the case report process. Given the time restraints and increased case load seen in most residency settings, I understand the difficulty of producing quality research in 2-3 years. However, I think the proper mentors are always able to guide their residents to success. There is no shortage of unanswered questions in the practice of veterinary medicine and at the very least, this requirement should prompt students and their mentors to make that vast hole a bit more shallow.

I do not understand how I can answer this as I do not know what was not submitted for credentialing for this comparison.

I think there is a wide range of quality in the resident papers, depending largely on the input / assistance of the supervising diplomates.

The publications that are submitted for credentialing are generally written with faculty or boarded internists. I'm not able to differentiate between them when I read the papers.

The question is oddly worded, but also implies that the reader is somehow aware which publications were used for credentialing and which weren't. There is no designation on a publication that it is a "resident" publication.

Too many of these are done just to give the resident something to work on and may detract from the mentor's research focus.

I think it is hard to generalize, and it is not obvious to me when reading a journal article if it was a resident project or not. I would predict that some provide great value, others, no value.

While many resident publications contribute significantly to our field, I believe many (if not the majority) are done with the intent to simply "check the box" to complete residency requirements. I think if the publication requirements were taken seriously by all involved (residents & mentors/Diplomates), the overall quality of publications would improve correspondingly.

Which is more important the scientific research or the publication. If the former, why not allow the research to be presented in abstract or poster format? Given the length of a residency program I don't believe the time allows a project producing a significant scientific contribution. to be planned funded completed and published.

I don't believe that they are all of lesser value, just that some of are because of either time constraints or limited desire of some candidates to publish due to clinical orientation.

The question regarding the scientific value of publications should have a response that would allow one to say their value is extremely VARIABLE. Some are very good and others do not add much value. Likewise, it is ridiculous to assume one can say the value of a set of publications when each one has its own merit that may be more or less than another one. These last 2 questions have no value.

I believe the quality of the resident papers is almost always a reflection of their mentors research activity. Residents fortunate enough to be working with a well funded, active mentor will be able to spin off a project that may become an important contribution to our literature. The poor sot working in a busy, for profit referral clinic will struggle mightily to develop a project and will have an equally difficult time finding editorial help on the road to a publication.

With the timeline it is hard to recruit sufficient cases for an impactful study in 1-2 years from a single center, and unless shared primary authorship is negotiated in advance, multicentre trials with multiple residents don't fulfil the requirement for primary authorship.

The previous question stem didn't quite make sense (case report vs. case report?). But I think I was able to get what you were asking anyway.

No real comment on my answer--just seems like the question is terribly worded

There is too much variability from individual to individual to make a general statement in my opinion - some residents publish remarkable, high value research, others simply complete a project and publish whatever they can to meet the requirement. Even for those residents that do not ever wish to work as a clinician / scientist or have an academic career - even as a clinical track person - I believe that process of writing a scientific paper and going through the review process is incredibly important to someone that is hanging out a shingle as a specialist.

Resident projects are often limited to feasibility within the time frame of a residency. Considering most residencies are 3 years long and many residents do not develop comfort within their residencies until the end of the first year or second year, there is little time to do the most meaningful projects such as prospective studies.

I may have a different perspective but while also advancing to become a diplomate in REDACTED I had to submit 3 case reports that were approved before being invited to sit for the exam. There is merit in the case reports as well - regarding a good understanding of the literature and critically evaluating articles BUT I feel that my ACVIM publication enabled me to dive deeper in to one specific topic that I now have a great deal of knowledge about. It's difficult for me to say that the resident publication was more/less/equal importance than other publications - it was just different. I also came from a large private practice residency where finding research material was much more difficult (due to lack of mentorship from the internists).

Sometimes it's clear that a paper is a resident project because it's something not really that helpful (like dexmedetomidine is a better sedative than placebo.) But other times a resident or even student project changes the way we do medicine (baseline cortisol >2.0)

I think a resident paper can be as well-written and meaningful as anyone else's paper, given appropriate mentorship and research report. If some folks have the opinion that resident papers are "make-work" and of poor quality, first, I disagree. I think the quality is highly variable (as is the quality of papers published by tenure-track faculty). And if some folks have the opinion that resident papers are "make work" of poor quality, then the mentors and the residents who author those papers (not the requirment itself) are to blame.

I don't even understand what the question is looking for.

I do not believe that the research and publications performed by residents should be undervalued in any way. You question is inappropriate because it leads the surveyed in assuming that information performed by a resident is inherently of poorer quality. A resident is a 'good' residency should be instructed by their mentors in this publication process, and not left to 'flail'.

Some publications submitted for credentials purposes will be extremely valuable and some will be less so...that said they are no better or worse than comparable pubs in the field.

It certainly depends upon the actual study and mentoring of resident with senior author. Some of these resident publications have been extremely important contributions and others represent almost a waste of time and poor mentorship. Get out of them what you put in,so to speak

Cannot make a generalization. There are certainly some resident publications that are weaker than non-resident publications, but vice-versa is true as well.

The residents are the future of our college. Although their initial attempts at writing and research are often flawed, their thoughts are often new, different and refreshing.

NOT questions are difficult. I think resident publications are not as helpful as other publications when I was reading literature for the exam I groaned when I could obviously tell it was a residency check the box paper. However, they could be useful if there were not other things residents are busy finishing up.

There is a lot a variation

These studies are by design too small. Relegating to case series at best. Mostly retrospective with a tremendous amount of flaws.

Too many variables to be able to determine.

It is variable depending on the type of publication and journal of publication that the resident uses - i.e. a case report versus a RCCT vs a hypothesis driven research project.

I think that this varies. Some of them are of lesser value, but this is not uniformly the case for all residents, & some of them are equal to or exceeding the value of non-resident publications.

Really depends on the study and how it was done, the investment of the mentors, etc. A well done resident study/publication is equal to the value of any other publication that is well done.

As they are performed over a short period of time, they are not well done and offer very little new data of worth.

I think there are very valuable resident publications as well as less valued publications from non-residents. As most medical understanding is generated one small building block at a time, the resident contributions are clearly a building block in the context of the overall internal medicine literature published on an annual basis.

Since I can't really know whether a publication was submitted for credentials or not, I can't really say, but there are a number of papers that seem to be written by a green author, who is 1st author, that are less useful or less rigorous than papers written by more senior authors on the team.

There is the impression sometimes that resident papers are of lesser quality but I think it is just recall bias. There are plenty of low quality studies out there and ultimately it is not the resident's responsibility. It is on the advisors and peer reviewers to make sure that the quality of the paper is acceptable.

Typically resident publications and well written and of good scientific method, however tend to lack clinical utility. Much of this is likely due to the time constraints that limit a more involved study in conjunction with clinical training.

I feel that there are far greater "trash" studies that are published because of the residency requirement. I would much rather have those who wish to publish to further the field do larger, more quality studies with more definitive results than to sift through the work that was done under a timeline for achieving the credentials and just to fulfill a requirement.

It does depend on the study design - some publications with residents as first author are very well executed and have high impact. I marked "lesser value" overall because if the mentor does not understand good study design, and is only publishing to help the resident, the project will be of lesser impact/value. I would say that resident-driven case reports are less likely to be of the same value as a Diplomate-driven case report.

Given that articles are peer-reviewed, I do not see how the value of one versus the other would change. I am not sure I understand the reasoning being the question.

The vast majority of ACVIM diplomats are never going to contribute meaningfully to the scientific literature. They will instead be managing cases. Perhaps the publication requirement should reflect the latter.

The quality of the study honestly depends on the mentor as much as or more than the trainee. Great trainees may be given crappy studies; poor trainees may not step up to the plate as much and the mentor may just push the publication through

The aims of the resident publication are, in my opinion, not to necessarily learn how to conduct research, but to understand study design, statistical analysis, control groups, and how to write and understand scientific literature.

I don't know typically which studies in JVIM are resident vs. other publications, so I would assume all is equal and valuable.

I am not entirely sure I understood the question...

I feel like the residency publication requirement is one more hoop to jump through to get boarded. That accounts for a lot of crappy papers, as most residencies are so clinically heavy that there is no time to do a "real" research project (come up with a relevant idea, write a grant, institute research, analyze date, etc). I came from a world of research prior to vet school and the "science" and thought that goes into most residency publications is poor at best and has little impact on the how and why of medicine practice.

Hard to answer. The value varies with the paper and the mentorship

My impression is that they are of lesser value but since it is not declared in the publication, it is not possible to accurately assess.

Each is different, some are awesome some are not, same for the rest of literature.

Sometimes it is apparent that a publication was done "just for credentialing purposes" based on the nature of the study/conclusions... so it is not that the studies are poor, it is rather that the studies represent a feasible publication within a 3yr time limit

This question is unclear to me, I am uncertain what you are really asking.

Q7 - What detriment(s) do you believe the publication requirement currently provides? (Check all that apply OR choose none of the above if you feel that there are no detriments of the publication requirement)

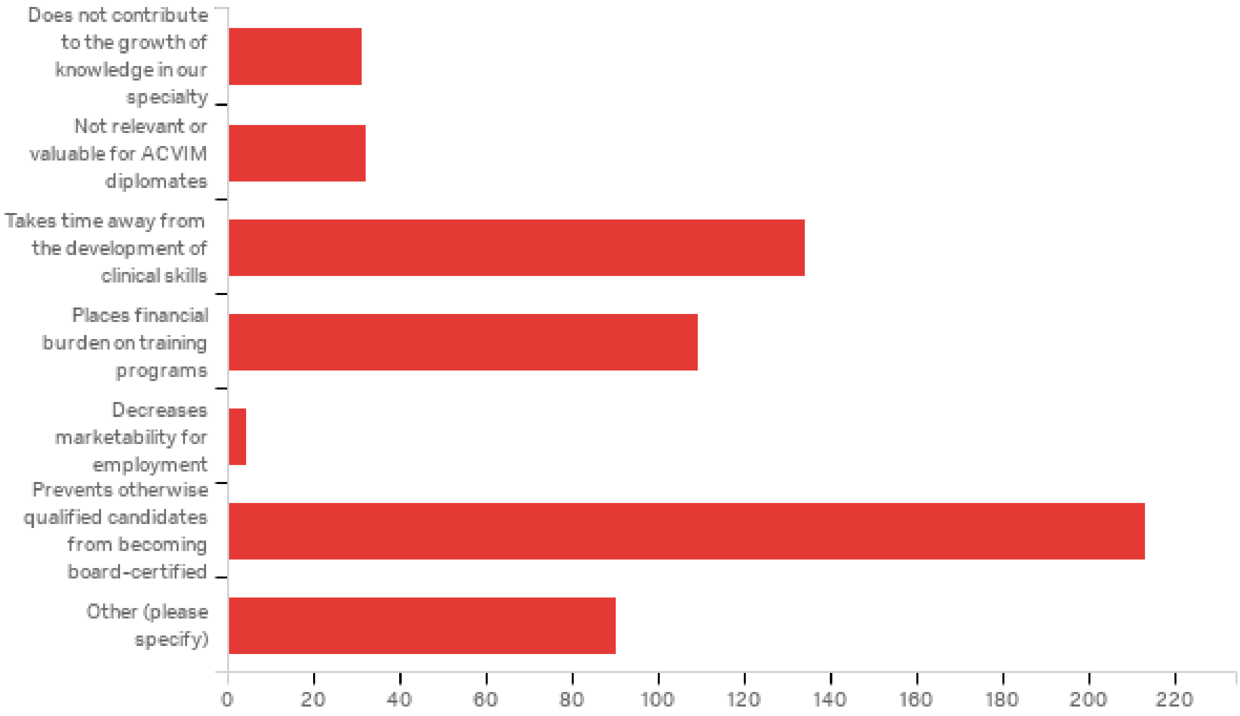

Answer	%	Count
Does not contribute to the growth of knowledge in our specialty	9.39%	31
Not relevant or valuable for ACVIM diplomates	9.70%	32
Takes time away from the development of clinical skills	40.61%	134
Places financial burden on training programs	33.03%	109
Decreases marketability for employment	1.21%	4
Prevents otherwise qualified candidates from becoming board-certified	64.55%	213
Other (please specify)	27.27%	90
Total	100%	330

Other (please specify)

Other (please specify)

Yes it can be time consuming and stressful but teaches extremely important skills. REDACTED and REDACTED colleges also have a much tougher publication requirement of multiple papers. Just as we plan to review the standard of the training programs this is an important aspect of producing quality specialists
Would somewhat agree with not relevant or valuable information - in that some of the publications in the form of a case report may not be providing a lot of valuable information for experienced diplomates. The time it takes I believe depends on the program and mentorship.
I think if a research project is not well designed and without guidance it could be a waste of time and delay the qualifications for certification. I felt that many of my senior clinicians werent research oriented and the guidance as far as statistical analysis was lacking and project formation was minimal.
Add a burden to otherwise already very busy residents
Takes longer to get certified.
In my residency, support (primarily time) for research was a limiting factor in pursuing these activities
Places time burden on already time poor mentors, which may then have financial burden implications
Stress on the candidate - however, I support the requirement.
Takes away valuable down time for residents to prepare for boards
Does not always produce quality or valuable research
Publication is a great concept, but very challenging in a private practice setting. Iacuc approval for prospective studies is virtually impossible outside of academia and many journals do not accept case reports. Retrospective studies are okay but provide some of the lowest quality of information to the field.
I don't feel there is a detriment to the requirement per se. I do feel that the programs should help the candidates to prioritize the publication and get completion of the publication early on. This helps to limit it being "the last hurdle" and therefore the thing that prevents certification.
Passing a publication does not impact clinical relevance outside of the university setting. In other words, regardless of publication content, an internist in practice is not judged by the publication - clients and employers care about clinical skills.
The timing was challenging during my residency (some time ago!) where you really were publishing a internship or case report/review for credentials because your research would not be completed.
The time constraint created by a 3 year residency encourages small prospective studies or case reports instead of larger prospective studies that may be more statistically meaningful and useful.
It is an important requirement that can be fulfilled with some planning.
Most of the research noted in these papers was undertaken by mentors. Much of these papers are written or at least heavily edited by mentors. Residents unfairly have to compete with proven writers and researchers to get their papers published in the the limited number of acceptable journals and for the limited topics / types of papers allowed.
I don't feel the publication requirement provides any detriment. I think every diplomate should at least be able to publish at minimum 1 case report or case series even if publication of their research project is not possible.
1. Delays board-certification by many otherwise qualified candidates. 2. Places additional stress (time stress and emotional stress on candidates, and perhaps advisors too). I did not check option 6 as the publication requirement does not really "prevent" board certification, but requires advising, planning, support, time and perseverance.
It is hard to say the publication is a "detriment". I personally feel open-access journals place an undue financial burden on residents and I also feel publication does not reflect clinical skill

Some of these could apply to any given individual or program but I am reluctant to check them individually.
Because submission to acceptance times are so long in many journals and most journals have moved away from accepting case reports or case series unless they are very unique, the publication requirement is a huge source of stress for our residents - yes, they submit their research papers but typically not until near the end of their residency so that this will almost certainly not be accepted in time for credentialing.As a result they are scrambling early in the residency to try to find a case to write up to meet the ACVIM requirement. There are less and less available journals to submit these to. Also, now that fewer and fewer ACVIM Diplomates are choosing academic careers, it is not entirely clear that ongoing contribution to the scientific literature is something that is expected of all Diplomates.
The publication process is important, not necessarily the result. If the resident's career trajectory is purely clinical, publication success makes no difference unless it prevents achievement of board certification.
Promotes poor quality studies from candidates in programs not set up for research (i.e. Private practice programs with mentors who do not routinely engage in research)
Lack of mentors in non-academic setting who know how to research-publish. Residents need a lot of guidance in the process and mentors have to make it a priority on par with their clinical work.
It is extremely difficult to complete a prospective study in 3 years from grant application through to publication. More assistance to the resident should be given in one way or another, or more time provided.
I would say that the experience is not relevant or valuable ENOUGH, but not completely lacking value or relevance
It "forces" a non-academic person to write a potentially bad one-time paper if there is not enough support from the resident's program
To clarify, given that the majority of trainees now go into private practice, where writing papers is not likely to be a high priority for most, I don't see a publication as relevant to the majority of residents. There will, of course, be exceptions.
Increases stress
Hard for residents in some institutions to obtain adequate mentoring or research funding
There are no detriments if we are to end up with highly qualified medical professionals. Learning how to write and how to organize data is a valuable skill for clinicians.
Nor relevant or valuable for ALL ACVIM diplomates
Even to though there is value to the publication, it does not outweigh the cost to either the candidate or the program
Places non academic programs at a disadvantage because of less resources for research.
I don't think there is a detriment to this requirement.
I think it places a huge demand on the supervising diplomate to generate a study with limited funding that will result in a publication.
The detriment I see is that with shifting demographics of the ACVIM, a situation is created whereby a diplomate that published a single, uncomplicated study can "take on" mentoring a candidate in a project. If the publication requirement is retained (which is my preference), perhaps a "specialist" should be expected to be involved with that aspect of training the same way we require time with a neurologist or clinical pathologist. The definition of a qualified mentor would need to be developed. In order to avoid over-burdening academic diplomates, the study material could be expected to be developed at the place the residents are training, with an on-site mentor, but the "writing specialist" could be overseeing the study plan and manuscripts.
Not enough time +/- funding to design and complete randomized, blinded, adequately powered studies so candidates are often limited to case reports and underpowered reports.
It may be that the time delay in publication is problematic, but otherwise all ACVIM members should be able to produce a publication quality paper at least once in their career.

University residents have a strong advantage over private practice residents who may not have the opportunity for research or adequate mentorship in becoming published.

As most of my residents go to large specialty practices, I cannot see these publications as relevant- unless they really do increase their reading skills and understanding of statistical evaluations for clinical disorder comparisons.

As long as a resident has the appropriate mentors, I think having a publication is very valuable. It is not the requirement that is the problem. It is the mentors at some programs. If you cannot get a small project or case report published after working with a prominent well published internist in a program which gives you the time to make your requirements (ie: pass qualifiying boards, write a paper) then the program must be reevaluated. I hated writing my paper but I learned a great deal from it and always saw the value in it. It was like eating brussle sprouts.

Sometimes stressful for mentors and or candidates.

It is very difficult to complete a truly useful study in the time available.

The time factor of publications is the limiting factor

It's very hard to do well in private practice. Some private practices that do a great job training for real world practice are horrendous for research, even retrospective (i.e. Paper based practice with no ability to search history of similar cases, like mine)

Takes time away from studying/reading, particularly in programs where there is not much of a support system in place for setting up residents early in their residency with appropriate AND FUNDED projects. REDACTED

See above comments. Too many useless publications that are either not significant findings, or "further research is required."

"Not relevant or valuable for diplomates" is hard to interpret. I don't not think it has much relevance AFTER completing the residency (eg. Doesn't strongly affect competitiveness in the job pool) but it is relevant during residency in understanding research processes better. Other: Can be difficult for private practice residents to repeatedly generate good projects (eg. Ones that can be done with available resources in 3 years). There is no funding for projects to they must be free and thus more likely to be retrospective or small case series. Other: Good projects often need funding and three years isn't long enough for someone to pursue a grant and a subsequent research while completing a clinical training program.

A significant burden if the residency program does not have mentors with research interests. Large private practice programs and university programs probably have a large advantage. Not realistic to expect a resident to independently drive the publication process, need appropriate faculty support and a big case load.

In human medicine they add fellowships to assure the resident get into a lab and research before back into clinics

I think the publication requirement is important.

I believe I have elaborated a sufficient amount for this survey. I have heard the argument for and against publications numerous times over 30 years. I have heard the arguments against publication based on comparison to human medical programs. While understanding that argument, I would also state that many human medical programs take much longer than our training programs. Might that aldo provide more exposure to 'critical thinking' in the workplace?

I do not think there is a detriment. I think it is not a burdensome requirement to ask candidates to publish something, if case reports and retrospectives are also allowed, it means they do not have to do benchtop research

The quality of resident projects is extremely variable- many want a case report or a minimal publishable unit- whereas some manage to complete some sort of retrospective or occasionally prospective study. REDACTED

provides relatively limited contribution to growth of knowledge.

Proposed idea to eliminate review articles will create undue burden for those in private practice residencies.

There is no way to know who actually wrote the paper and how much influence the mentor had on the process, so I worry about the integrity of the publication requirement. I also think it does not teach much and is an undue burden on candidates intending to work in practice.

The residency research requirement contributes to lower quality/class studies, ie. more case reports and prospective studies, as opposed to higher quality prospective work.

The financial burden MAY be real but my house officers REDACTED were able to have 3 separate mini-projects funded and published while I was in practice.

No detriments. It can only help the resident.

May contribute poor quality publications to the literature from residents who are either minimally interested/just getting their requirements met and/or are short on funding needed to get the work done.

However, those candidate typically procrastinate and then are in a mad rush to do the paper. Mentors should make it a priority and provide the proper guidance to get it done. Then it will cease to be an issue

REDACTED, it breaks my heart to say this. However, I think our specialty has moved on to mostly train private practice specialists. The cost and pain of forcing these disinterested individuals to perform research isn't worth it and doesn't achieve the goals of training them to be inquisitive and skeptical readers of literature.

There is variable support for research depending on the program. Individual needs to be extremely proactive and motivated without proper support.

no guarantee of funds for a project/study to complete for publication REDACTED

Depending on residency programs, certain residents can have difficulty completeing this requirement if they are not at an academic institution or do not have mentors that focus on continued research outside of their residencies.

I don't think that there is a detriment. REDACTED Had the publication requirement not been there, I feel pretty strongly that I would have missed out on these opportunities.

I believe ACVIM is still struggling with what it wants to be when it grows up. Initially, Diplomate status was sold as equivalent to a PhD at the universities. So research/publication was an obvious requirement even though it wasn't enforced for many years.

places financial burden on resident mentor, particularly for those studies that aren't retrospective

I certainly do not wish to disparage the work of anyone who attempts to get published, however, the type of resident projects that can get completed in a short timeframe often do not make substantial contributions to the literature.

Not all candidates have the same strengths, but all candidates have strengths. I wish there were some mechanism by which candidates could complete "2 out of three" or 3 out of 4 critical elements. Yes, overachievers could still do all 4 if they really want to. Then those with interest in pursuing careers necessitating publication as a critical element could be allowed to use the publication towards their board cert process. Others whose strength lie in memorizing the extraordinary minutia required to pass the multiple guess certifying exam could skip the pub require. And visa versa.

Lengthy time-to-publication for some journals may cause a delay between submission and acceptance that causes candidate to miss a deadline (don't qualify because their publication submitted but not accepted). I've seen that happen.

Ability to put together a quality publication depends so much on the mentorship and funding of the faculty at that institution. A well funded faculty member with a lab set up is going to have many project ideas and options for residents while faculty that don't do research are going to leave residents struggling for resources and mentorship. It is detrimental to all of us to publish something just for the sake of publishing.

Creates controversy because people who are not interested in mentoring research and choose to do it poorly or inattentively then create "evidence" for their claim that resident research is poor. If mentors held themselves to high standards of discovery, residents' standards would be similarly high.

While I do not see tremendous value in the publication requirement, I also didn't see value in my cardiology rotation. But it taught me something, exposed to me information I normally don't deal with. So I appreciate the

value it afforded me. I also think it's a great "hoop" to jump through and it pleases me that it keeps some people from being boarded.

The publication requirement as been a part of the SAIM Residency Training programs for decades. The candidates in excellent programs succeed, because they are in excellent programs. The inability to reach Diplomate status strictly from a publication deficit is actually a deficit of the PROGRAM.

In theory, the publication requirement should foster the desire for research and publications in the future, but the process is so cumbersome and the reviewers for the journals can be so derogatory, it does the opposite. I have no desire to publish after the comments I had to read about my work. This is especially true when one reads some of the ridiculous papers that get published with a poor study design or no discernible point, etc, and I wonder how these were able to slip through the system, but good papers and reports are rejected.

Clogs the literature with small, underpowered studies with little relevance.

I do not see a detriment.

But I believe that if a three-year program can/does not provide ample time for clinical skills development even with time taken out for research/study/writing, then the merit/rigor of that program should be reassessed.

I believe the benefits of writing and publishing at least 1 paper far outweigh any potential detriments

Although I believe that doing research is the best way to be a knowledgable consumer of research, so I would include research skills as part of being a fully qualified diplomate

Why should the veterinary literature be cluttered with poor-quality studies produced by disinterested residents?

I believe there are NO negatives to the publication requirement. Those opposed are simply trying to accommodate the ever growing movement to make everything easier and dilute the quality of our profession.

I think it has value. I think strong mentorship is important to make sure that people who are not graduate students per se and don't receive much in the way of research training or time allotment do not get derailed from their goal of becoming a veterinary internist. I'm not sure how to improve that oversight, but I think having projects that are ready to go S well as allowing the super motivated person to design their own might prevent people from falling through the cracks.

Since most diplomates are destined for private practice, the relevancy of fostering a research interest or sientific writing skills are questionable. There are other ways in which critical thinking could be fostered - I do not believe that cardilogy specialty required publication any more.

Does not foster true curiosity---rather a mad dash to finish a project, write it, and submit before residency is over

If proper assistance from the mentor and training program is not available this becomes very difficult. I think the accepted publications are too narrow and should be expanded.

Q8 - What benefit(s) do you believe the publication requirement currently provide(s) (Check all that apply OR choose none of the above if you feel that there are no benefits of the publication requirement:

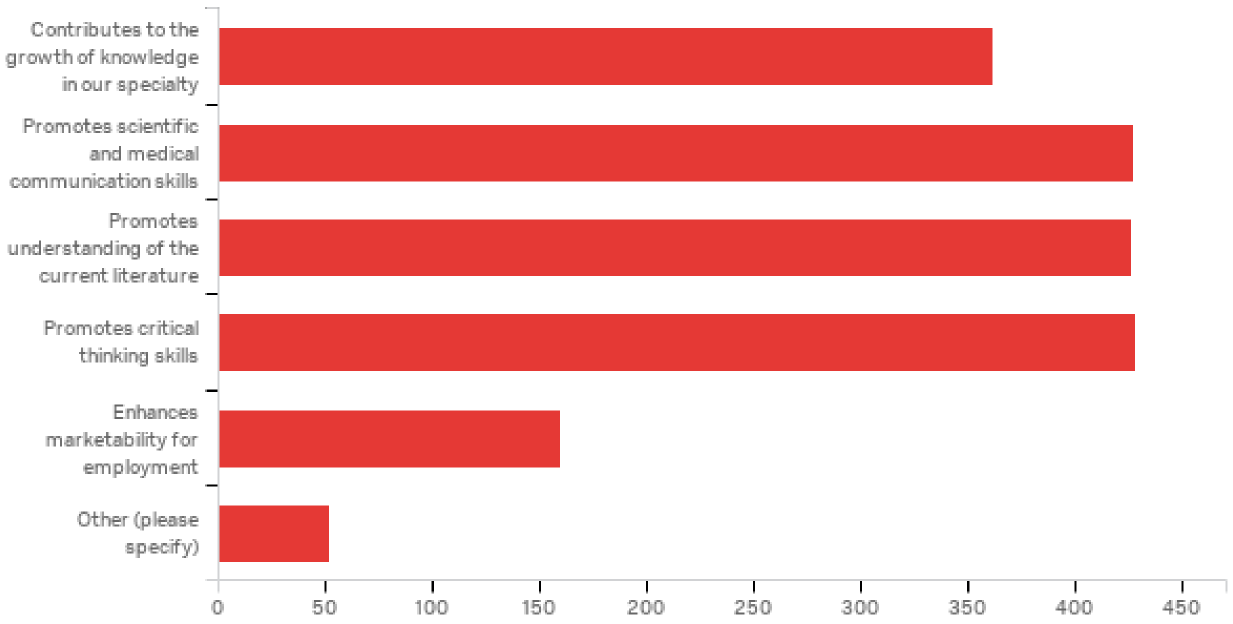

Answer	%	Count
Contributes to the growth of knowledge in our specialty	72.78%	361
Promotes scientific and medical communication skills	86.09%	427
Promotes understanding of the current literature	85.89%	426
Promotes critical thinking skills	86.29%	428
Enhances marketability for employment	32.06%	159
Other (please specify)	10.28%	51
Total	100%	496

Q9 - Do you feel that the current publication requirement should:

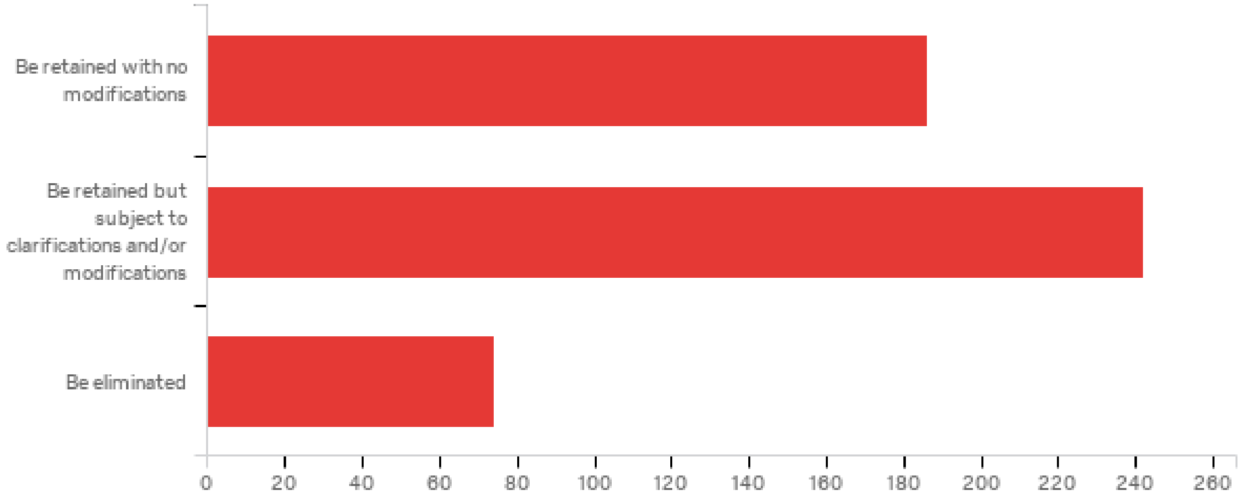

Answer	%	Count
Be retained with no modifications	37.05%	186
Be retained but subject to clarifications and/or modifications	48.21%	242
Be eliminated	14.74%	74
Total	100%	502

Q10 - Please elaborate on your response to the previous item, if desired.

Please elaborate on your response to the previous item, if desired.
REDACTED, I feel that the case report requirement provided a much better method for evaluating a candidates clinical skills and ability to communicate than does the publication requirement
I don't believe that an original research project should be required, as review articles can give as much benefit to the writer as a new publication. I believe that being 2nd author on a bigger and more complete publication is likely to be more valuable than being first author on a poor publication, so wonder if it might be possible for candidates to be 2nd author on 2 or 3 publications, rather than being 1st author on one publication.
The requirement should be broadened to include case reports and review papers. These may not be at the same level as a research paper, but these are very helpful to the family veterinarian in general practice.
I think we should keep the requirement for a resident scientific study but not necessarily that a candidate needs to be "published" in order to become board certified.
It would be desirable to have a wider range of accredited journals to which to submit. Given the ease of accessing data nowadays, particularly with more and more journals following an open format, to me it's less important which journal to submit to and more important that the information is out there in the public arena.
Please don't make it less stringent, it is already very achievable
i do not believe lack of publication should prevent a perfectly qualified ACVIM resident from becoming boarded. i DO think the candidate should have to go thru the entire process in attempt to get a maniscript published. perhaps there is a way for ACVIM credentials committee to judge what rejected paper is still worthy of a candidate becomin boarded. i don't have easy answer for how to do that at this time and i realize this would be very difficult to do/defend. i only suggest this however to prevent submission of pure junk to a journal for the purposes of becoming boarded. perhaps looking at in what way a publication is rejected (ie flat out rejected vs. accepted with provisions.
I think if the publication requirement is continued, then there should be more defined guidelines, for example I understand why the case reports were eliminated, its often very costly for clients to perform every diagnostic test to get to a final answer. Structured guidelines for statistical guidelines and project development.
I am in favor of retaining the requirement with 2 conditions: (1) accept all types of publications (case reports, series, retrospective, bench, prospective studies and reviews (2) significantly expand the list of journals that are acceptable to include open access journals etc.
Literature is better than NO literature. Therefore, I believe that publication should be a requirement: Additionally, itshows commitment to the profession and brings some standards to the residency programs. Without it, everyone will have a residency and the quality of residents will decline. I do not believe that the quality of publications are strong in general for veterinary medicine overall. I also don't believe that the publication requirement itself makes candidates marketable or any real authority on a topic per sey. It certainly does not make them a better clinician. They do contribute to the understanding of the system, scientific writing and overall knowledge. Additionally, it will make them more critical of literature (certainly a valuable asset) and for these reasons I agree that publication should in the current model be a requirement.
The review process was miserable and made me never want to write a paper ever again. I wish that had not been the case since I have thought of interesting research topic but am not going to pursue them given how unpleasant the review process was.
I honestly feel two ways about it. The process did make me a much better consumer of published studies. That was the most valuable result. Does that make it necessary? I do not know.
I believe that it is important that candidates understand the research and publication process but also understand the difficulty faced from smaller residency programs in coming up with prujects/subject matter. As REDACTED, I also worry that this requirement has resulted in some subjects that are really (for lack of a better term) stupid and do not contribute one bit to the advancement of veterinary knowledge.

I feel that the publication requirement should be dropped for board certification as it is not necessary for the great majority who become private practice specialists. However, residency training programs may still require completion of research as part of their program. Alternatively, a residency program may allow residents to track as academic or prvt practice. Individuals that declare as academic would complete research and publications and would have greater resources available (faculty time, funding) and would become diplomates with a distinction.

I do not believe that single case reports or review articles should suffice for the requirement.

One can be board certified and able to work as a boarded clinician if the publication is accepted within 2 years of certification. Most residency requirements are so demanding and waiting for acceptance of a paper or editing is time consuming. Boarded, publishing pending could be a term for such circumstances.

The biggest problem is finding an apporpriate journal or source in which publication is relelvant. The journals are currently overwhelmed with case reports and many topics of interest to a resident are not subjects that are in high demand for publications.

Case studies and case series must continue to be allowed, as should retrospective studies. Publications submitted prior to the start of residencies should also be permitted, if they meet topic and quality requirements.

I feel that over time the requirements for publication have become more strict, to a burdensome degree. For example it used to be that any peer-reviewed journal was acceptable. Now it is a peer-reviewed journal that the credentials committee likes (the committee may say that it is because so-and-so journal has an impact factor of X, but this seems arbitrary and isn't in the GIG as far as I know). I bet that my article that was accepted for credentials REDACTED years ago (a review article published in REDACTED) would never be accepted now.

Perhaps submission of a well thought out project idea instead of a paper or paper acceptance? Becoming more relaxed on which journals count for the requirement would help as well. A peer reviewed journal in a topic that is "not internal medicine enough" will currently not allow the candidate to pass the requirent. I'm torn between desire to contribute more to the profession and the understanding that this is just not possible often in private practice residencies.

I'm not sure what modifications would eliminate the issues. However previously there was a link with the publication being a requirement to sit the certifying exam. I think (but maybe wrong) that this would mean fewer people would have passed the test but not be boarded.

Can delay great clinicians from sitting their exams/completing certification when publications in non-university settings are irrelevant to job performance as an acting clinician.

Publication eliminated but a project required.

There is more to being a specialist than running a scope of probe; performing a spinal tap of giving chemo....

I worry that the publication requirement may put pressure on candidates to "tinker" with results of their research/studies to make them publishable and contribute to misleading information. We need to consider an avenue of appeal in this process for work done that gives results that do not give information that is worthy of an article or if there are problems getting submitted articles accepted for publication.

For many journals only a small percent of papers submitted get published and the time from submission to publication can take months.

case reports promote clinical reasoning and could be used in replace of retrospective or prospective research

Eliminate case reports and non-peer-reviewed review articles as options.

I think that critical assessment of literature can be taught via epidemiology and statistics and in journal reviews with mentors. I think that scientific writing can be demonstrated by the resident at a level "thought equivalent to publication quality" by their advisory committee/mentors. I think that it contributes to unnecessary or weak publications and huge pressure to publish for people who just want to be good clinical internists. I think that weight of publication value no longer deals with getting hired into a faculty/academic position (which it does for some) as many go into private practice. Make it a choice, encourage it, demand demonstration of writing skills, but do not require publication.

I am not really familiar with the current discussion so I must admit that I do not know the current proposals for modifications or clarifications

Because most of our new diplomates are destined for careers in private practice, I think that continuing to train everyone for a possible academic career is not the best use of resident or faculty time. Individual academic programs could choose to advertise one or more of their SAIM residency positions as including a research and publication requirement, or develop residency/master's degree or residency/PhD programs to train people specifically interested in academic careers.

I believe is a requirement that improves our profession thru literature enrichment. If retained, does not makes sense to say "with no modifications." The medical field is dynamic and everything changes with it, even the way is written and investigated. If eliminated, it will be a significant loss. It should be modified and standarized, if possible, to improve the quality of work performed, research, and information obtained, which will translate to, hopefully, better medicine (diagnostics and treatment outcomes.)

I believe the requirement should be eliminated. At the very least the requirement for journal acceptance should be replaced by the acceptance of an ACVIM article review committee. Finally, someone needs to explain to me why the ACVIM allows certification of neurologists and cardiologists without a journal requirement. That's not fair and these subspecialties obviously don't feel there is a need for publication.

I think that review articles should not be permitted and you should not be allowed to take the speciality examination until a publication has been accepted.

If the membership thinks it is a burden, without benefit, then amend it.

While there are some potential benefits to the publication requirement, with the majority of residents going into private specialty practice, most will never publish again - so, scientific writing skills are not essential for most. It is becoming increasingly difficult to find the financial resources for residents to perform prospective or lab-based studies, even in the university setting, resulting in many residents performing retrospective studies (some of which can be valuable) or writing up case reports to meet the publication requirement. Publication fees (e.g., REDACTED) now add to the cost. It would be ideal to encourage residents to undertake a research project and publish findings (if there is something meaningful found that would add to the literature) - but, if the research/publication does not work out for some reason or is not feasible in a particular residency program, the candidate's board-certification should not be held up for years while trying to publish something.

A standardized publication requirement could be made available by more collaborative research with people in academia and private practice working together. Even collaboration between institutions (universities or corporations). Lets say a resident at REDACTED could do a study and enroll patients at multiple centers (academic, and private practice) by collaborating and coauthorship. Similarly, a resident in private practice could do a similar study and get to access academics and other private practices. What if ACVIM had a committee that universities and corporations agreed to collaborate with so that a mentorship team could help a resident design a study and then that study could be conducted in multiple centers? This might even raise the "bar" on professional relationsips in and between universities (REDACTED), corporations (REDACTED) and different practices to contribute to veterinary medical research in a meaningful way that advances our profession for patients, clients and veterinarians.

1. Greater clarification of the type of publications that will be judged acceptable is required. A candidate should be able to submit the publication at an earlier stage than final acceptance for "pre-clearance". (I know this happens to some extent but most residents and advisors seem unclear on it). 2. The ACVIM should provide more support for resident projects and publication costs. 3. Greater responsibility on the resident advisor to assist the resident with the entire publication process.

In my opinion, I do not feel residents should be required to publish. I do think that those individuals who wish to obtain a master's or PhD (very much like MD/PhD student) should be required to publish. In no way do I feel a resident's publication will have any impact on the clinical acumen or skill set. As such I am opposed to the current requirement as is. I do recognize the need to continue to foster academic publication but I fear "forcing" it upon individuals who have no interest in research will only serve to lessen the quality of the research overall. I personally would be in favor of bringing case reports in some capacity back as I feel these would better elaborate

on an individuals ability to formulate a differential list and problem solve. If a publication is required it should absolutely be linked to an individual's ability to sit for the certifying examination.

I feel that there are other ways of accomplishing the benefits of publications.

ACVIM, in my opionion, should not be supporting/promoting a stripped down assembly line-like process to create board cerified internists. Just because a process is challenging, is not reason to through it out. Any licensed veterinarian can parctice how they see fit, under the scutiny of the public, the state board, and possibly their local peers. A residency trained veterinarian is able to practice with more experience and knowledge, similar in sorts to a season practioner who has learned more by lifes experiences than under the influence of some overseeing doctors. Each could therefore be more successful than a less experienced veterinarian, and could publicize or promote based on their experience. But why dilute the standards of our higher ACVIM group so that some individuals can become board certified? So they can then make more money with letters behind their names? ACVIM or the lack of board certification does not prevent them from practicing, doing endoscopy, public speaking, etc. But becoming an internist should not be a simple task. Whats next, simply a single general test because the certifier is too hard? I do remember case reports... Is our future requirement going to be simply completing a residency, no doubt for which some have be create for simple financial gain of the practice as the primary reason and less so to train individuals to carry our torch. Once an internist, i do feel better trained to read scientific literature, review literature, and write/participate in writing literature having gone through the process before of writing a publication. Am I a good writer? No, but that's not the point. I do think I am a better internist for having had the experience and education derived from the literature requirement. I do have to read and understand articles all the time and feel the background I have gained from being part of the process has aided in my ability to do such and taught me respect for the process. Maybe this should have gone in the section below but I'm working on a REDACTED, if you couldn't tell by my typing..

I think/feel/believe that the intended benefit does NOT inure to any key stakeholder (patient, candidate, specialty, training programs, etc.). The mass-production and time-limited articles do not provide significant literature quality (e.g. statistical power; even the hypotheses are often weak; do we really need another mechanisms-of-disease rehash?). The training programs are generally neither well resourced, nor interested, in promoting this aspect of training.

I feel strongly that the individual programs should require that residents submit one or two manuscripts for publication during their program. I actually think that THAT should be the requirement - that the residency requires them to submit a manuscript, not that it necessarily get accepted in time for credentialing. That would provide the experience in scientific writing and review that is required without the pressure of wondering whether it will be "in time" and "good enough for ACVIM". This is especially true since so many of the journals now do not accept case reports. I think that case reports and case series both meet the objectives of OUR requirement - that is that residents get experience in scientific writing and the review process, with hopes that it will serve as an entry point to ongoing contributions to the scientific literature in the future. We have a graduate degree with a requirement for a research publication as well - this is usually submitted near the end of the residency so is rarely suitable for ACVIM credentialing.

Allow/do not discourage case reports.

I had not had the chance to write a scientific paper before my residency (other than single case reports) and found it extremely beneficial in the fact that I discovered how rigorous I needed to me (thanks to my mentor!), I believe I would never have learned the process on my own, the fact that my mentor was there to help was extremely beneficial. Even if it was extremely time consuming, I do not regret it !

I believe the resident should make an ernest effort to participate in the scientific process, including submission and defense (through documented completion of the review process) of their publication. I do not believe a resident should be disqualified from achievement of board certification because of their failure to successfully publish (this is more their mentor/program responsibility). I believe the residents qualification should be satisfied by documentation of publication submission, review and revision. The submission should meet minimum scientific method publication requirements (these would need to be consensus decided but should be purely method, focusing on the structure and organization of the submitted research, not necessarily the relevance of the content). Most that go through the process will want to be published and will make an effort to provide

publishable information. A failed but legitimate publication effort is as much a lesson to the candidate as a successful one. If the candidate wishes to pursue academia, at some point they will have to "sink or swim" relative to publication. Publications success should not have a "sink or swim" impact on a purely clinical professional trajectory.

Do not know what the current "rules and regulations" are, i.e. are there only certain publications a resident can submit a research paper to, are case reports not allowed, retrospectives or only prospective studies?

See all of my above

I think all residents should be required to perform prospective research during their residency. It would be nice to see some standardization of the requirement to hold all residents equal.

I support prospective or retrospective studies, case series, review-type articles for specialists and non specialists, and unique case reports for this purpose - they all teach critical thinking and writing skills for future dips. Do not need a "one size fits all" plan.

With the increase in non-academic Residencies, I would be interested in knowing if the rate of delayed achievement of obtaining Boards due to missing the publication requirement is more common in University or Private practice Residencies. It should be easier to create a project in larger institutions, but I suspect it [like so much of Residency Training] is more related to the involvement and energy of the Mentors and Residency Advisors.

I think that clear guidelines for journals allowed as well as the strength of the publication should be established and upheld. While case reports occasionally add to the growth of knowledge, they are less likely to and do not give the author the opportunity to work with statistics and the actual significance of findings. A case series or retrospective study are more worthwhile publications.

We should allow publications that are not directly related to our specialty. If someone has worked hard on a study and publication that is in another field, for example during a PhD or other graduate degree, that should be accepted, regardless of subject matter.

I don t feel that a single case report to qualify is enough. I like the REDACTED requirements better.

Although there are obvious benefits to the publication requirement, I think it puts additional, unnecessary stress on the residents - who are already overloaded with work. Some residency programmes include a master, and it is not uncommon for residents to go on to do a PhD after (or before) their clinical training - these are in my opinion better ways to encourage research and publications (during and after (or before) the residency, respectively) - without having to get it published (and possibly rushing to do so) just to get the certification. As long as it is at least possible to sit the board exams before having the publication approved REDACTED I do however think that the current requirement is an OK alternative - just not my preferred one.

I think residency programs should be more actively supporting the publication requirement and steering the resident from day 1 onto working on their project. Many institutions allow the resident to linger without a project for years.

The publication requirement is not fair across the board. It is much easier for someone at an academic institution to publish vs. someone in private practice. I also found that there are a large amount of residents trying to publish with not a lot of journals to be submitting to (i.e. I felt that the current journals were being overwhelmed and not publishing some articles that they may have otherwise been interested). REDACTED I worked SO hard to get my paper published without much guidance on how to do it. I would actually be quite livid if this requirement was pushed aside since I worked so hard to get my diplomate status. I know plenty of other residency-trained doctors who are not even trying to get their publication. If they get their diplomate status handed to them without a paper, I would not be happy at all. With that being said, publishing a paper in private practice is more daunting than someone in academia. Maybe we could focus on having tools in place for mentoring even in the private practice atmosphere.

I am aware that Residents in some programs submit dubious publications for credentialing. So long as the publication is subject to minimum reasonable QC, I vote for its retention.

There should be responsibility placed on the residency facility to provide opportunity, support/ nurturing, guidance, as well as specific time-sensitive "goals" set for resident to help this be successful for actively-engaged candidate.

Just because it's a pain doesn't mean it isn't part of the package. And I went straight into private practice.

See above. Firmly believe it is essential

It is SO dependent on the program and its support. If there could be an arm within ACVIM (possibly outside of the resident's program?) to seek support and guidance for publications then possibly it would be less painful for some. Other residents will continue to have no problems either because of their own motivation/interests and a strong support system

I believe that publication requirement does not make one a better clinical internist. Those that enjoy research and publications will always continue to do so, those who do not will continue to struggle to get a single mediocre publication out to satisfy the requirement and never publish again.

If I look at some of the potential justifications or benefits to having a publication requirement as suggested by this survery, it is challenging for me to find support for a publication requirement short of the one-time contribution to knowledge. While the original motivation for the publication (which I suspect was to have residents contribute to the growth of knowledge in internal medicine) is laudable, the current reality, as alluded to above, is that most residents seek/find careers in private practice, where having a publication is, I believe, unlikely to be, or won't be, of benefit to them. The publication requirement then, I'd argue, does not prepare the majority of trainees for their chosen career path, does not enhance their marketability (in any market- does one publication really make me a better prospect for a given job than someone with no publication??), does not (as a one-shot requirement) promote medical communication or writing skills (isn’t a skill something that is developed by doing over and over again?), does not promote understanding of current literature or critical thinking skills above or beyond what should happen with a journal club, or other elements of the resident's training, and does not foster/nurture a life-long commitment to research and publication. I'd worry, actually, that many residents (and some of their mentors?) see the publication as a burden, a hurdle to jump through, and that perception/attitude would stifle longer-term interest and commitment to research and writing. I suspect many resident research papers will reflect some offshoot of a mentor's research program, and I further suspect that few residents actually get the opportunity to think of an idea, develop it into something testable, do the testing and results analysis, and write, which would be the full gamut of the "scientific experience." If my assumption is correct (doing a mentor-related project) the resident then misses out on a substantial part of the scientific experience, and suffers if the project has flaws that prohibit publication; that resident pays the price if that work was the only/best opportunity for a publication and the candidate then can't obtain certification- for lack of a publication- because of circumstances that were out of the resident's control. I know there will be exceptions, driven by good mentors that will force a resident to take that offshoot and develop into a research project, but even then, the vagaries of having experiments work, getting study subjects enrolled, getting reviewers that are receptive to the paper, etc are also factors beyond the resident’s control, but the resident pays the price (no certification) if such things prevent a project from being completed or a paper from being accepted. I am not suggesting that experiencing just a part of the scientific experience has no merit, but for someone planning to go into private practice, it is hard for me to see how that snapshot benefits most of them on a regular basis (I respect that there will always be exceptions or special circumstance that would counter this argument). For someone with an interest in research, being exposed to even a part of the process is likely to be beneficial in preparing them for their chosen career, but harder to see that for someone going into private practice.

I wouldn't be adverse to modifying the requirement.

I just didn't think it was horribly difficult to fulfill this requirement, and I got no financial or mentoring support (other than someone reading final drafts). If a candidate isn't interested enough in exploring a medical question/mystery, or doesn't have the willpower to write a scientific paper, I think something is wrong. I question that person's ability to stay in the field and contribute to the field long term.

I think we should retain the publication requirement, but there is nothing wrong with a case report. Also, I feel there is nothing wrong with a project that has to deal with oncology, neurology, surgery, dermatology, anesthesiology, etc. It all is beneficial to help the resident learn, and all of it is of some benefit to the literature.

Simple case reports should not be allowed

I would favor eliminating case reports and literature reviews even though they are now discouraged. I would insist on primary literature only much as Oncology does.

prospective studies should be encouraged, but not required

I strongly feel that the publication requirement should be part of an internal medicine residency. I understand that most residents go into private practice and are not academia bound and therefore becoming a strong clinician is a priority. That being said, what personally helped me become a strong clinician was to also learn to became a scientist, think critically, and understand how research informs our clinical decisions. One can argue that you can learn that by simply taking time to read and evaluate the literature, but I would beg to differ. Actually doing the research forces you to be at the front line of the process, which is invaluable and cannot be replaced by simply reading papers in a journal club. I find it interesting that there is such a push to get rid of the publication requirement. In other professions, such as clinical psychology, programs place much emphasis and value on training students in a "scientist-practitioner" model. Perhaps I am just old fashioned, or getting old, but the trend to make the residency easier is concerning. Bottom line, doing research is one of the hard parts of a residency for most people, but certainly not impossible or unachievable. I think proving that one can go above and beyond in your training is what sets internists apart from general veterinarians. The skills I learned from doing research still support and enhance the way I practice today, even though I am not in an academic setting.

Though publications benefit some candidates (those geared to an academic career), in many cases the publications do not contribute to the candidate's future career nor do they contribute significantly to the field of veterinary research. Two broad benefits of the publication requirement are the improved understanding of scientific research as well as improved writing skills. For candidates without an interest in academia, these benefits could be obtained by other means (graduate courses reviewing scientific paper, writing classes) that would not delay certification.

I think it should be an optional requirement for each individual program. We will continue to require it, but we are an academic program REDACTED, so it is of value to us, and an understood requirement for residents that chose to come here. But, even here, we want the ability to sign off on, for example, on a resident whose research spontaneously combusted late, in an irreparable way, or that has a great paper that has spent 2-3 years sitting in pre-publishing at, say, REDACTED (two recent offenders). The requirement currently forces us to chose the "fastest" journal, rather than the "best" journal, for publishing our research. I think it should not be a requirement for, say, all practice residencies.

Being in private practice, I do not feel that the publication requirement made any difference in my ability to be a good internist, but it did help confirm to me what I did not want to do, which is to be an academician who spends a lot of time writing papers! Also, it did help teach about critical thinking and scientific analysis, at least to some degree. But, I also did a REDACTED degree and a resident project which taught me more about the scientific method & research than a single publication did.

There are inconsistencies between the current GIG and the SAIM credentials instructions, eg. with respect to suitability of review articles (GIG states acceptable if in accepted journal; credentials instructions states that they are unlikely to be accepted). The acceptability of case reports is also unclear. I think that both review articles and case reports should be considered acceptable.

I think that if the candidate has published in a currently accepted journal on a topic relevant to IM, it should be considered adequate for credentials.

Unfortunately there are many varieties of training programs, and many varieties of mentors and their level of investment in a resident. Guidance and feedback is SO essential to this process...but not all residents are given this- and of course not all residents are 'easy' to invest in. A discussion of goals, outcomes and expectations WRT publication should occur at the beginning of every residency program- along with all else. It is up to both mentor and resident to ensure deadlines are met, research/publication is proceeding and be pro-active in order to complete (be engaged) and allow for a positive outcome/success.

I think it is acceptable for residents to have to undertake a prospective study but not have a publication requirement. This would allow residents to participate in more meaningful long term research being performed by their mentors.

See prior comment about requiring a mentor for the research project/publication that has some qualification to be in that role beyond having a single publication of their own. Again, no different that expecting time with a specialist in another discipline.

I have no suggestions for modifications but would be willing to listen to options. I feel it was a highly valuable experience for me (years ago) and as stated above, I do feel that every ACVIM member should be able to produce a peer-reviewed publication at least once in their career for all the reasons I have indicated.

Less restrictive on type of publication due to lack of research opportunities for some residents.

See responses above

The mentors must be given guidelines and the residents must be allowed to have a certain amount of time off clinics to complete projects, write etc. This must be mandatory for all residency programs if it is not already. In talking with other internists, my impression is that some of the private practice residencies beat the heck out of the interns and residents for profit and really don't care what happens to them after they leave. I was fortunate to complete my residency REDACTED which made getting your boards a very high priority. I cannot think of one past resident from my program that did not get boarded within a reasonable amount of time. (And I'm pretty old now) Again, the mentorship rules must be more standardized.

In my opinion, removing the publication requirement from the credentials would mean change our goal of training "clinical scientists" towards training "pure clinicians". Obviously it is not always easy to not go overboard and train "pure scientists" that lose their clinical value... and it is not easy to combine everything in this short time. Options could be to change the publication requirement to a requirement to perform a clinical study (to be defined exactly), that does not necessarily need to be published (could argue that this would be a waste of time for the advisor?), but maybe would reflect the true work of the resident and not the combined effort of the resident and advisor (both are under pressure). Could possibly be defined as a smaller, more clearly defined study that could be achieved both in practice of in academia...

I do not think that case reports or reviews should satisfy the requirements for board certification. I think that residents should have to publish a research paper in order satisfy the requirements.

Case reports should not be permitted as publications. Promote the clinician scientist model by requiring a research based publication.

I am not sure of the current requirements so I would be open to modification if that was helpful to those that are trying to get this requirement eliminated.

I would increase the publication requirement to two publications, and require a true scientific study for one of them (i.e. no case reports or short case series).

I would suggest that rather than a primary author paper, a co-authored paper would be sufficient.

The benefits outweigh the detriments. I personally have no interest in doing research but I depend on those that do to further our understanding of biological processes.

Information shouldn't be pushed into publication just because it is a requirement to credential; however, all residents should be required to go through the publication process. Ability to go through the complete steps for publication should still be critically evaluated by unassociated ACVIM Diplomates. Papers should be streamlined. Why do we publish a 20 page paper when 5 would do to pass on the NEW relevant information.

Given the inconsistency/variability of the many institutions to provide ready access to sound advice and funding for projects early in the program, I think making it a requirement is a mistake. Do we know what percentage of diplomates publish that one paper and never again? Those interested in academia or research from a private practice setting would not be prevented from publishing during their residencies and continuing to do so after board certification.

See above comments.

I think the publication requirement is an important part of trying to keep the bar "high" for overall excellence in our specialty. The bar is getting pretty low these days.

Novel case reports should be acceptable... Maybe they are already and I just don't know?

I don't think it should be eliminated and would be open to modifications though I'm just unsure what those might be. I would like exposure to the scientific approach to research but not have it seem like diplomates need to be extremely proficient in research - we're making clinicians not PhDs.

Not all diplomates are interested in pursuing research oriented positions. I would suggest that an alternative option regarding scientific literature review be acceptable or that research count toward "weeks" but not be required

I do not understand why we keep trying to dumb down the requirements to obtain ACVIM credentials. I am not an older diplomate by any means but simply cannot understand why these issues keep coming back. First less case reports then no case reports then no need to have all requirements in place before sitting for boards. Why don't we just put our ACVIM certificate in a cracker jack box. We need to select for the best and the best should be able to rise to the occasion. It is meant to be hard, that is what separates the specialist from the non-specialist.

I would also be fine with "retained, but subject to clarifications..." depending on the clarifications.

Simply training residents for clinical skills should not be the only goal. The residency has already been expanded from 2 to 3 years to permit completion of studies and exams. Publication requirements are already easier than for REDACTED

Case reports should be allowed

I honestly have not kept track of the current requirement. If it is only 1 article, not consisting of a single case report, then it should be retained.

It could be an option, amongst other options. For example, residents could fulfill a certain number of requirements out of different options. Options could include a publication, case reports, CE lectures, book chapters.

The goal of the publications is to act as a hurdle for obtaining boards. The requirement is outdated and is just another way of trying to prevent candidates from obtaining their board certification. The ACVIM exam is detached from internal medicine specialty practice reality and so is the publication requirement.

As above

I believe that the detrimental aspect of the publication requirement to the candidate is greater than any benefit they will receive.

I think the residency program and requirements need restructuring. 1. Core SAIM- can be completed in as little 2 yrs -- like it used to be before all the time off and expectations espoused by some for a prospective research project..... 2. Elective fellowships after core to pursue subspecialty clinical training- this could include Ultrasound/advanced imaging etc rather than trying to cram it in to SAIM core to extent most residents desire for general practice. 3. Streamline core training for residents who want to pursue research training- they are almost ready to restore is they doe 4 yr pre vet+ 4 yr vet+ 1 yr internship + 3 yrs residency.... prior to PhD

I know lots of people want to see reviews dis-allowed, but I think well done peer reviewed overviews of a subject area are useful and worthwhile.

I feel the publication requirement should be retained. I would find it acceptable for case reports and small retrospective studies (too small to apply valid statistical analysis) to not meet the publication requirement but have no real objection if most diplomates feel they should be acceptable.

Hard to answer if there should be clarifications and modifications when you do not know what these might be.

My suggestion is to require completion of one of several options, ONE of which is an accepted scientific publication. This would allow more flexibility to pursue an option relevant to the candidate's career goals. Options still could be designed to foster appreciation for the scientific process (proposal, design, publication, and critical review), encourage participation in CE/conferences or academics, business-related endeavors, etc.

Options to accomplish this might include: - publication of original material or research in a scientific journal - designing an experiment and writing a mock (or real) grant proposal that is reviewed by either the granting agency or the specialty board. (This could be a one-time submission, or a multi-step process resulting in acceptance.) - reviewing/critiquing a sample grant proposal and/or publication (as an editor) - successful public presentation (and defense) of an abstract or literature review (at the ACVIM Forum?) +/- a written follow-up. - primary teaching responsibilities (design of a lecture or wet lab) with submission/approval of content and review of performance. - TA responsibility for an undergraduate or veterinary class - design and present an educational event for the public - successful design/submission of a business plan (e.g. to open a practice or establish a non-profit) - open/custom (design to be submitted to the board for approval)

Please retain the option for a review article. If I had not been able to complete REDACTED, then I would not have been able to attain board certification status. Not achieving board certification would have been a great disappointment after completing a fairly rotten residency. Mentoring was poor in my residency, and the REDACTED that I completed was done after the residency and was done on my own. REDACTED

I think the idea of trying to build a clinician scientist during a residency is unrealistic... we need to focus on clinical skills and drop the idea that one pub makes someone understand the scientific process! Also, I am worried by the idea that a mentor could write the paper and we would never know. I personally am aware of some circumstances in which this may well have occurred. Please DROP the publication requirement! BTW - I am on faculty at REDACTED and STILL feel this way :-)

I also feel that the case reports should return

I do feel that there is an onus on training programs to prove that they support the writing and development of studies. I would hate for residents to be held from their diplomate status if their manuscript is written and sitting on the desk of their mentor for review...

Time is not provided for this requirement by many residencies and new employers. Further, writing can be challenging for some and, in some case, made a nightmare by overaggressive mentors. I fear that "board eligible" is becoming an acceptable term for some specialists that have everything except the manuscript requirement. The client wants a capable specialist, not a "noted author."

I think the publication has value. I also thought case reports had value--just had issues with the variable judgement of them. It is not much to ask for 1 publication in 3 years. and then testing. it should teach the candidates to budget time, stay on task. it is doable if you start early enough and are proactive with your mentor.

understanding scientific process is what sets a specialist clinician apart from a "middle tier" or advanced practitioner level individual. Although the middle tier is less well developed in REDACTED it is coming and we need to ensure specialists offer something more (higher academic level) than good clinical skills

While it can be argued that publishing a research paper has intrinsic benefits; the question of true value to the candidate is in question. If the paper produced is high quality then it adds to the growth of knowledge, but not all of them are. If the candidate wants to go into an academic program then the paper has value, but most candidates do not. While it does promote scientific communication and understanding of literature, so do many other aspects of the residency thus leading one to wonder how much it truly adds. The biggest concern is that it often hinders board certification for otherwise qualified candidates and many times the process is out of their hands. The candidates must rely on their mentors and other authors to turn the paper around in a reasonable time. They are also at the whim of journal time lines which with some journals can take over a year from submission to final acceptance. Outside factors should not dictate an individuals ability to become accredited.

There currently seems to be a problem with candidates finishing their residencies, often times passing their exams but taking several years to meet the publication requirement. It seems to me that there is a breakdown somewhere that is allowing this to happen, so while I am not sure what modifications we need to make, we likely need to make some changes so that there is less lag time between residency completion and completion of the publication requirement. I DO NOT think that the publication should be required prior to sitting boards as it is in some specialties, and that the timing should still be kept as it is.

ACVIM was founded by individuals who were at the forefront of the evolution of clinical practice, performing the basic scientific and clinical studies necessary to solve their own clinical problems. Regardless of what this survey concludes about peoples' perceptions of the purpose of the requirement, the publication requirement is based on

the idea that every Diplomate that came after the founders should make a meaningful contribution to the evolution of the practice of veterinary internal medicine as the founders did. By that viewpoint, trainees unwilling or unable to make such a contribution should not be certified. If, in the modern era, our group as a whole feels that scientific contribution is not a core requirement, or if this group of incredibly smart people cannot figure out a way to support otherwise qualified trainees sufficiently so they are able to complete the requirement and benefit from the process, then it should be eliminated but I believe it has value.

I dont understand the complaints. It should be continued. It does require time but more importantly it requires discipline to finish.

You generally don't find publication requirements in human clinical specialties mainly because a clinical training program should focus on the clinical training. We are trying to accomplish in relatively short clinical residency training periods what are often left to "superspecialty" fellowship training in himan medicine

I'm actually not sure how I feel about this. For those intending to do academic practice there is no question that having a publication requirement in place provides some necessary motivation for both the candidates and their supervisors to prioritize the publication requirement. For those who truly have no interest in research, are in private practice residencies, and intend to practice in private practice, the publication requirement has more questionable value (and is likely much more difficult to attain without the same resources that are available in academia). But I am not sure if there should be an exemption for the latter group, because that may cause new problems.

I actually think the REDACTED standard of 2 publications would be better for the ACVIM to adopt

My first inclination was to recommend that this process be retained without modifications. However, clearly this issue is hotly debated because there are issues to resolve. So, I changed my answer accordingly. I believe we can come to a consensus that still allows residents and their mentors to contribute to scholarly work while satisfying everybody's needs.

Eliminating the requirement does not necessarily eliminate the option.to publish for more meaningful reasons.

My answer to the above depends on what modifications are being proposed.

Allowing case reports to be used as the publication requirement

Research and publications should be encouraged and supported for those individuals interested in academia or for those programs who require participation, but for the practicing internist/clinician "jumping through the hoop" for the sake of credentials is a waste of everyone's time (and journal space).

I honestly don't know exactly how the publication requirement is worded, so if someone wants to modify it, I think that's fine. I personally learned a lot from having to publish and would not be able to be doing the job that I'm doing now if it hadn't been for the requirement and I never planned to do anything other than private practice. I think that most people in private practice would probably say that the publication requirement doesn't enhance marketability, but it my case, it did when I decided to change my career path.

Having come through the process of specialization when 2 publications AND case reports were required, I have some hesitancy in stating that the publication requirement should be eliminated, but I'm not certain that it has the same value in preparing the resident to be a specialist. As the vast majority of residents will not pursue an academic career and would rather spend their residency honing clinical skills, I can't see a true benefit except for those that want to pursue research or publications as part of their career plan. For those residents in a non-MS or non-PhD program, I don't think there is value in publishing. I believe residents develop an appreciation for research in Journal Club, Board review, and other activities at their training site and from attending the ACVIM Forum. Requiring a publication becomes a burden for some, especially if their research does not bear fruit during the residency.

I wonder if a resident could learn the scientific writing process, and also learn how to critically evaluate the scientific literature, by taking a course instead of submitting a publication. Each university may have courses that would be appropriate. I found an example: REDACTED

In order to provide opportunity for greater magnitude of study, residents should be allowed to show meaningful participation and requirement for professional writing to include interpretation of statistical data within a larger study in order to meet the publication requirement.

Potentially addressing the types of publications accepted for meeting credentials or the publication process itself may be beneficial (discussed further below).

Internists that desire a more research oriented career path will surely will be pursuing an advanced degree affording ample time and opportunity to publish.

Although I do believe that there is value to the publication requirement for all prospective diplomats (including those going into clinical careers), I don't feel that the value is great enough to counteract the detractors, especially with so many going into clinical fields.

Case reports, case series and retrospective studies should absolutely be allowed. Review articles that are peer reviewed should also be allowed.

I certainly feel members of the College should contribute to the literature and constantly seek to improve our understanding of the disorders we treat. Why we require our residents to do so before they join has always been unclear to me. As one of the "old heads" in the College, I only see us adding requirements, e.g., ultrasound, other advanced imaging interpretation, etc. while the body of literature continues to expand. Publications are always a product of the institution and while I'm sure the resident learns as their drafts get torn apart by experienced scientific writers and reviewers it is silly to say that the resident is contributing much. Alternatively, I think we could further develop programs to teach and test residents ability to critically review the work of others. A skill far more important for their future than suffering through the process of getting one paper accepted.

Improving the quality/calibre of the veterinary literature (e.g. sufficient power, complexity) often means that small/simple projects that educate residents in critical and scientific thinking/writing skills are difficult to be accepted for publication. e.g. the project was accepted for ACVIM abstract but not deemed worthy of publication - now what does the resident do? Conversely - the publication requirements promulgates small studies (of potentially minimal significance) continuing to occupy our journals. Could conference abstracts be considered a credential-worthy publication?

I think the publication requirement is essential. It gives readers an understanding of research and the rigors of writing a scientific paper. Elimination of the requirement seems to be a part of the "dumbing down" of ACVIM's requirements. Becoming a diplomats expressed should be rigorous.

I believe that it was very helpful for understanding the publication process and learning how to be better at reading through literature. My concern is knowing residents that have gone through the process and for various reasons not get published and are not boarded.... can we have a review of their submission to see if it is adequate for becoming boarded? Maybe consider a review paper of a topic submitted to ACVIM that would be available for diplomats?

I dislike the recent attempts to "rate" the worthiness of pub depending on what journal it did or did not get into. Anything that gets through a peer review process should count. The world still needs case reports and assay validation studies as well as scientific breakthroughs and new disease discovery. The writing process is a learning curve and a single pub requirement does not seem any more onerous than the breath and width of current information that candidates are seemingly on the hook to know. But it is a unique strength that not all people, clinicians or otherwise, can master. Thus, options may need to be considered. For example, could middle authorship also count? For example, perhaps the ACVIM could promote specific multi-facility/ vet school /clinic projects where part of the pub requirement is met by contributing to the collection of cases and data for ultimate publication? Perhaps there could a case summary section of the certifying exam wherein the candidate needs to succinctly prepare a summary of a series of cases "on the fly". Perhaps a section of the certification process could be to pick a sham publication and the candidate could assess or grade it for strengths and weaknesses (re: stats, study design, group size adequacy, consistency with the current state of knowledge or published results for the topic, etc). There is more than one way to accomplish the learning curve part of the communication process collectively know as "publishing". It's the learning curve part I would like to retain.

Case reports should not be frowned upon if they involve a rare or novel presentation, and they level the playing field for candidates not in an academic residency program

The process of doing a research project is the most valuable aspect of the requirement. Having the paper actually published is less important.

I am inclined to think that maybe a publication should at least be in process or in review before the certifying exam can be taken. But I do go back and forth on this stance.

It would be wonderful if there was a way to keep the publication requirement but expand on the mentorship so that people are not left with only the faculty they have in their facility. If there was a way to have an idea bank from other schools and to collaborate with other schools that would be great. Also there should be some standardized course that everyone takes on how to write a paper or stats or something, and nice to have mentorship with that. For example- all residents take an online course on writing papers. Professor X at the Xavier school for gifted youngsters has 5 projects he has in his mind. Resident Y at Redacted has no real options for research with her own faculty. Professor X posts his projects to a board and Resident Y agrees to do one. Over 3 years Professor X and Resident Y email or talk on the phone until project is completed. Also could establish a program with major labs like REDACTED as a source not only of funding but data.

If there is a concern that resident publications are of poor quality, then the rigor with which resident papers are scrutinized for acceptance should be increased. Again, it is not my opinion that resident papers are generally poor - I think they are as good as the mentors require them to be. But if some folks are holding a low bar resulting in a sometimes-poor product, I don't think the solution is to eliminate the requirement. The solution is to raise the bar.

Keep it. Limited value to most people but so is dermatology.

Publication requirement should be re-linked to the examination. That is should be completed prior tot taking the certifying exam.

We need to define the purpose of the requirement! once that is clear, we can then assess the whether or not the requirement is meeting the purpose. I personally believe that the "best" purpose would be a service requirement that fulfills the discovery mission of the college.

I don't think the requirement should be eradicated entirely. This would decrease the value of existing diplomates and the effort they put forth to get their publications. If, say, certain journals accepted residency publications with a different set of requirements, or a certain subset of reviewers, it may be a more feasible goal. These requirements should somehow take into account the circumstances of a private practice residency. These are valuable programs, as they actually prepare a candidate for the "real" world of private practice internal medicine, but they are generally weak on people who are able to help with publication requirements. Publications seem very much dependent on "who you know" or what names are co-authors on your paper. Case reports may be a more acceptable publication in these cases, for example.

This has been a stumbling block for a number of candidates. Hopefully with new guidelines in place and with early recognition of a study and a measureable timeline for completion this will markedly improve success and remove this stumbling block.

In my opinion, the primary goal of the publication requirement should be the improvement in scientific writing skills. So I believe modifications could be made to this requirement to help with these skills without the rigidity of publication (e.g. allow review articles, case series, etc)

I think a more extensive list of peer reviewed publications should be acceptable. As long as they are truly "peer reviewed" which means two or more experts in the field as reviewers.

I think residents need adequate mentorship to truly do research and often they are not getting this mentorship. I have been on all sides of this equation REDACTED If the publication requirement were a review article or case report this would be better as the resident could learn to write, contribute to knowledge base but not have to perform research which is not helpful without mentorship.

It can be a challenge for residents in REDACTED to get published even with a lot of mentorship and support. For many private practice residents I think it is probably the biggest obstacle to getting boarded and for many of them it is a pointless exercise. If residents actively want to do research they should be encouraged, supported, and probably receive further training i.e. PhD or MSc training.

I think the residency programs need to SUPPORT the publication process - the goals of the publication process are not always met if there is a lack of support, guidance, and mentorship in growing the above listed skills. Simply asking the resident to do a project and publish it is not enough.

The publication requirement should be retained with strict guidelines of what is acceptable so that it may add more to the scientific community. For example, prospective studies only are acceptable or case reports and retrospective studies meeting certain criteria.

Waste of time. I think our trainees should spend much more time with hands on clinical skills and I believe critical thinking/ evaluation of publication come through journal club/m&m rounds.

I think that there should be better structure created for the ACVIM programs to ensure that the project is appropriate In size and structure. Having projects ready to go would help

I believe there should be a requirement but we should re-evaluate types of publications rather than a publication for sake of a publication.

I would love to see more flexibility where residents who are less research focused can complete the requirement by writing a critical review on a subject for any peer-reviewed publication, even if it is a periodical.

I think case reports should not be accepted.

The requirement should be met prior to allowing the candidate to take the specialty examination. There should be greater clarity regarding what constitutes an acceptable publication, and greater transparency in the process of reviewing whether a publication is suitable. The publication should be reviewed by a panel vs. by a single member of the credentials committee.

I think the College has become too rigid in the interpretation of what Journals are considered acceptable for resident publications. If the Journal is peer reviewed and indexed, I think the resident publication of new knowledge should count towards certification.

On one hand, many of the papers are marginal contributions and the residents were forced, kicking and screaming, to write the paper, and seemed to learn little in the process except how much they hate it REDACTED. It did not change their future contributions to the field, as they never ever will write another paper. On the other hand, I had to do it, and they should too. I can't come up with a reason to keep it, except a vague sense that it builds character. Perhaps it is like learning how to do laparoscopy. Scary, stressful, may not be relevant in many practices, but maybe the resident will find out they like it, and then they have a new useful skill.

As long as case reports are still considered acceptable I do not feel that anything needs changed.

The current guidelines regarding exactly what publications are/are not deemed worthy to be counted toward credentials remain vague, I believe.

I see no reason why a resident candidate would be unable to create a clinically relevant paper worthy of publication.

Too many candidates are not as fortune as I was to have a mentor to help with the process

It would be a disgrace to aim to please the lowest common denominator and eliminate the publication requirement. It is vital that veterinary specialist understand the scientific process more than any other medical professional. We are required to sift through copious amounts of poor quality literature. The publication process is fundamental in imparting the necessary skills to interpret this information correctly.

The research and publication process during my residency was one of the most helpful parts of my training, considering I needed to learn how to implement the scientific method and statistics. This was essential for me understanding medical literature which should be read and consumed throughout one's career to stay up to date with standards of practice. I would be highly disappointed if this requirement were no longer implemented during residency training. We already see many difficulties in misinterpreting statistical outcomes in publications, and I am not sure any trainee can understand these issues better than by producing a study.

I suggest that review papers be eliminated from the allowable publication formats. I believe that review papers should be written by experts working in the field, and that asking residents to read the literature and prepare a review does a disservice to the quality of the reviews in our publications. Further, since I believe that residents

should be in involved in a research project with the goal of learning the scientific process and learning how to be a critical reader of studies published by others, reviewing the literature does not help support these goals.

I would need to reread the actual wording to give the best response, but feel we could do better in terms of giving residents broader opportunities for publishing.

Case reports should be eliminated as acceptable publications.

It's possible that one may learn more from a case report vs sitting in the basement of the hospital looking through microfiche records for a retrospective study. Maybe have the option to do a case report vs publication?

We keep talking about dropping case reports, increasing the pass rate of the exams, dropping publication requirements. Pretty soon it will become just a training program you pay to get through. I'm not sure we want to go there. REDACTED

I would like to see a system similar to cardiology college with multiple routes to satisfy scientific requirement. While there are benefits, the publication is a hurdle that is of no relevance to most internists. Moreover, it is subject to the whims and opinions of reviewers and editors - a candidate may have excellent critical thinking, writing skills and learn a lot about the process of research; but if the reviewers of a journal that we arbitrarily decide is acceptable do not deem it to be novel or relevant then they are going to be rejected. This does not seem to be a fair part of the credentialing process.

I believe publication should be accepted PRIOR to acceptance of candidates as eligible to take certification examination

I think more types of publications should be accepted and the list of acceptable journals should be expanded.

Q11 - Do you have any other comments regarding the publication requirement?

Do you have any other comments regarding the publication requirement?
Redacted, I feel that the case report requirement provided a much better method for evaluating a candidates clinical skills and ability to communicate than does the publication requirement
Depending on the type of residency (Academic, as part of PHD program, Private Practice), the type of submission may vary. As the private practice sector continues to grow, clinical papers, even an interesting single case report is valuable to veterinary medicine and gives the resident experience with writing, submitting a paper, etc. Let's face it... there is a significant need for private practice specialists (see job board). Training for this type of internist is certainly different than training for an academician. Allowing single case reports, review papers are certainly doable in the busiest residency program.
I think that the publication is an important part of credentials, both for the learning experience of it, as well as standing out as a specialist. Since it seems to be holding back so many from becoming board-certified, I would think that there should be more focus on fixing the residency programs that do not foster this type of learning as opposed to making credentialing "easier."
I really don't think the publication is a detriment. We signed up for a residency knowing this was a requirement - the complaints about the publication requirement are absurd. This is one part of many requirements to becoming board certified. And, I believe it's important to play a role in the scientific process - most projects are supported by faculty (either intellectually or financially or both) and many of these projects would never happen without residents trying to achieve board certification. Thus, I think resident projects can also help drive the discipline of veterinary medicine forward.
In my experience, if you do not have a good mentor or are not inherently good at writing research papers, this is a place where many candidates fail.
I think clinical cases are just as important as either retrospective or prospective studies.
My opinion is it cannot stay as it is: we should either make it easier in accepting most types of publications and most if not all journals no questions asked (preferred solution) OR completely do away with it
I believe that the amending the whole system would be better for the profession than the issue of publication vs. no publication or what constitutes a publication. Wouldn't it be better if the residents had to contribute the body of knowledge by identifying cases and enrolling cases in larger, better funded, better powered, longer duration studies that ACVIM select to study For example: ONGOING ACVIM studies: 1. The effects of mycophenolate on Lyme nephropathy Inclusion criteria: rule out other disease processes (infectious, imaging, etc), positive C6 antibody testing, biopsy confirmation, agreed upon dosing scheme and follow up data for duration of residency 2. The effects of phosphorus restriction on PLN Inclusion criteria: rule out of other causes of proteinuria. Biopsy performed. Data for duration of residency, etc, etc. Just 2 examples but these cases could be identified and tracked over the duration of the residency but also EXTENDED for the duration of the life of the animal. Maybe ACVIM could identify these patients and send questionnaires to clients. I believe that our clients would enroll in these types of studies to further the profession. Maybe each resident could be responsible for identifying and enrolling 5 quality cases, marked for these studies INSTEAD of a publication. Maybe ACVIM could have hundreds of ongoing studies that are approved through the foundation and maybe partially funded. They could have a department dedicated to tracking these cases for the better good well beyond the residency and someone in the institution could be responsible for keeping records. This would be the price institutions pay for having a residency program. Wouldn't it be nice if we started to look beyond whether doing a small, double blinded placebo controlled study that may still be underpowered and contribute to dissemination of mis-information to the community performed by a resident is a right of passage and started to look at how the residents could alternatively add to the wealth of the knowledge in a meaningful way. Food for thought.
I was very grateful that two of my graduate committee had studies I could participate in to accomplish my requirement. We had to have two back then...

If the publication requirement is eliminated then other specific competencies/milestones/entrustable professional activity thresholds that require minimal competency in evaluating evidence in the literature should be implemented.

Despite all of its problems, I believe that the accomplishment of publishing a paper is an important step for candidates. Perhaps a solution would be to consider the option for candidates to go back to case report writing, if they cannot publish something. As important as I believe the process to be, I would also hate for an inability to publish to be the only reason a person who has fulfilled all other requirements to not be able to achieve board certification.

Of all the various themes given for the value of the publication, I feel that the contribution to the body of academic knowledge and learning of critical thinking skills are most important. If a residency program just becomes an exercise in learning particular clinical skills and familiarity with an already extant, and thus circumscribed, body of knowledge, then nothing about being a diplomate truly distinguishes us from an experienced and competent generalist. Whether the current system, wherein retrospectives and publications that are not hypothesis-driven are considered acceptable, is the best way to address this need to train residents beyond simple currency and clinical skills is obviously open to debate. Personally I do not feel that it is. Thus my answer that the program/requirements need clarification.

A candidate's ability to complete research and publish a paper depends largely on their mentor. This can vary greatly from one program to another and likely reflects the faculty's publishing requirements. I have seen many board qualified specialists that make the personal and financial sacrifices to complete a residency but end up with a paper that is not accepted for publication. The candidate has little to no power to change this and there are no consequences for the residency program (and no transparency for future candidates to evaluate that program). Many of these folks are great clinicians, but become alienated from the ACVIM

I think that the publication requirement should stand as is. I also am a fan of the previous required case-reports. Both aspects encouraged me to become more critical in my writing process.

I feel very torn. There is a terrible need for information in our profession and I do believe our residents need to learn the skills to write scientifically as well as critically evaluate the literature. However, the ability of the training programs to be able to provide the support (financial, intellectual, moral) needed for every resident to produce the appropriate publication to meet the requirements is questionable. This does not mean that those that cannot are not capable of producing excellent internists. I see two things really wrong with the publication requirement: 1. Pushing out publications, sometimes from excessively small clinical trials or poorly put together retrospective studies, just to meet requirements can result in poor information being relayed to the profession. 2. The journals can make it extremely difficult for the resident to get an acceptable publication - both in the length of time to go through the process and in the bias that is out there for considering something to be acceptable. I don't know the solution - I am probably most in favor of keeping the requirement but really assessing what it is we want a resident to demonstrate AND marrying that with what would be useful information to have in the literature --> remembering that informational update/topic reviews can be very useful for general practitioners and will be contributing to the literature in an educational way, even if it is not educating other internists.

I think I voiced it above. I think that as a resident advisor, I would require my resident REDACTED to demonstrate ability to write an article of publishable quality, to have had it reviewed critically by appropriate people within the program or as exchange of documents between resident committees at different locations. For example, I would ask friends at University to review at publication level, work of my resident, written as part of their requirement for the internal program. This would be designed as a completed or not completed check box. Publication would be encouraged but not required.

I actually felt more strongly about retaining the case reports but that ship has sailed. I feel strongly that our profession should foster clear and concise scientific writing skills and critical reflective skills.

Becoming a diplomate is a challenge and holds great value. For all the benefits of the publication requirement, I believe it is worth the burden.

Getting rid of the publication requirement means that the ACVIM accreditation is less valuable. If more people do not get boarded because they can't publish then perhaps they should not get boarded!

I commonly see the argument against the publication requirement that so many "eyes" look at the paper before it is published that it is no longer solely the resident's own work. I think this misses the point of the benefit to the

resident, the advisor, the ACVIM, and the profession that ensues from the publication requirement. Also, no one publishes a manuscript "solo", so it wold be unrealistic to expect residents to do so. I think they obtain a realistic idea of what is involved in getting a paper published, and are more informed and critical readers of the literature as a result, even if they never publish another paper.

See above

It didn't pan out. Time to drop it. The concepts should be tested in the general exam (statistics, types of studies, what is EBM, etc.). We do (as a group) need to ensure our members can effectively review the literature, but writing an article ourselves is not the path.

It seems to me that ACVIM has over-stepped its boundaries by bringing in their own set of criteria for whether articles are acceptable or not. If they are submitted to a journal and accepted after scientific review that should be adequate. The resident would have gained the experience of submitting a manuscript and going through the review process. To then say that ACVIM doe not like that journal and is going to review the manuscript to make sure it is "up to THEIR standards" is inappropriate.

As above. Definitely improves critical thinking skills and communication skills. I feel that the publication should be accepted by the end of the last year of the residency program (not later on). There is no reason why candidates should not be able to achieve this within the 3 year residency program. At the risk of sounding a bit harsh, only residents with poor time management skills would struggle with achieving the publication requirement during their program. If it is a goal from the very start, I can't see why it cannot be achieved.

It had it's place at one time when the College was smaller and the need to place academic clinicians was very high - in these instances, I felt that meaningful contributions to the literature were made vs superfluous case reports. It is my impression that the primary focus of many publications from residents today is to get it published "somewhere"; with little regard as to the scientific quality and potential impact in the current scientific literature.

Publishing a paper helped me to better understand and interpret the medical literature.

As good clinicians we have to be good consumers of research. Other have proposed alternative paths to a publication, such as attending training on study design and methodologies. I am of the belief that the only way to truly develop a deep learning of the scientific method is to actively be engaged in the process. If the publication requirement is eliminated, I do hope that it is replaced with a series of training courses that are equally arduous to a prospective study.

The publication requirement is one of the few evaluations of 'critical thinking' remaining in Residency Training evaluation. It IS a time and [increasingly] a financial burden on training programs, and a test of how invested programs are to their Residents (since overseeing and steering a Resident through a project can be challenging). Seeing marginal case studies being referenced at gospel on the list serve, and conclusions based on small numbers of individual pets being cited as proof of efficacy shows that even Boarded ACVIM Internists may need more exposure to critical thinking and how to evaluated the strengths and weaknesses of the published literature.

If surgery interns in the private practice setting can churn out publications (and they do), then so can a medicine resident. Having worked in academia and private practice, the time is there if the powers that be will provide it to the resident. The resident advisor should make this part of the program one of the priorities- something to discuss at regular meetings to check on progress and to provide ideas.

I think it is so incredibly important to have residents be involved in the scientific process. People who are uninvolved in the scientific process, in my experience, tend to take the literature at face value and blindly believe what the authors write. However, exposure to designing, developing, and interpreting a paper help to develop residents' critical thinking skills and help them to also see the inherent difficulties, limitations, and grey zones in paper writing. I strongly recommend keeping the publication requirement.

For me personally, I was able to submit REDACTED as well as REDACTED. I happen to use these for my credentialing process. However, I did complete REDACTED and found it very helpful in the lifelong appreciation of the scientific method and my ability to critically assess new information over the course of my career.

I am very concerned about the conversation that seems to be occurring that suggests we need to make it easier and easier for all candidates to become diplomates.

Yes, it is dependent upon mentorship but if you take residents on & have a residency program, this is part of your job!!! Residents also need to take an active role - be motivated to get it started early & done.

My own process was very painful for me and I hated it. I had no foundation in scientific writing, my support system was weak and unimaginative and I floundered for a long time. REDACTED

I honestly have mixed feelings, but I think the time has come to eliminate a publication requirement as a part of the GIG. Individual programs can make publications a requirement (an approach I'd favor), but hard today for me to see the justification for an across-the-board requirement. It is more difficult for me to see/appreciate how a single paper prepares the "typical" resident for a career in private practice, or provides the resident with the skills needed to be successful as an internist- in private practice. I imagine (I could be wrong) that mentors in private practice programs will object to publications because "research" is not built into the typical day/week/month of most, and their residents don't have the structure to support scholarly activity comparable to that found in academic programs. Many nuances, and I've already spent too much time doing this 5-minute survey!!

Even though I have been boarded for many years, I take pride in my publications. At the time I wasn't so keen on writing the papers, but now I am glad for it. I have yet to publish anything else. However, sometimes I see something think I should write up and I am not so intimidated. I think, well, I published something before. I could do it again. So, perhaps for some people, it is a way to get the ball rolling on publications, because if you don't start during your residency, the whole process might seem way to intimidating later.

My personal experience: REDACTED Even though my situation was not ideal, my opinion is that the process of research, writing the paper, publication experience etc. is very important for the experience needed for board certification.

It is very valuable to the development of well rounded and well educated residents who represent ACVIVM and all of us.

At the time, I did not appreciate its significance to my career REDACTED. I have always been excited with research and publication--and even more so now. Writing skills have significantly improved.

Keep it!

If the requirement stays, loosen up the criteria, and please loosen up the retentiveness of the Credentials committee. We should not be asking residents to "contribute to the profession" if we don't require it of diplomates. So the main reason for keeping the requirement, if there is one, is to learn critical thinking and scientific writing. So, if they have a first author publication as a grad student, student or intern, before a residency, ACCEPT it. Also, if they've published bat migration patterns in Brazil or, heaven forbid, surgery, ACCEPT it. They've still demonstrated critical thinking and scientific writing.

In my opinion, the publication requirement for the purpose of the residency training/ diplomate acknowledgement is absolutely unnecessary and even counterproductive in some instances. A residency training is a clinical training with the purpose of training experts in their respective field in the clinic. A research project and the publication thereof, is a scientific training preparing people for a career in research and we have tools like the masters degree or the PhD to train people the this field. Due to a lack of time and experience, resident projects are oftentimes poorly designed and/or executed and therefore often rejected. In addition, as the body of scientific knowledge in veterinary medicine grows and more and more people actually choose a career in science, residents who are already busy in the clinic, start to compete with full time researchers. This leads to enormous pressure on the residents. Therefore, many residents start to write reviews or publish in journals that also are acknowledged by ACVIM are barely relevant to the field. A good internist does not need to be a good researcher and vice versa and both rarely go together.

This comment is not politically correct (I'm sorry). I think answers will depend on whether the participant is in academia or PP. If the participant continues to write, publish, and successfully mentor resident research projects, their answers will be different from the answers given by someone who resentfully wrote a resident paper many years ago, and now views the requirement as getting in their way of hiring residents and brining residency training of others, to completion. The survey, as it is currently designed, would be similar to a survey conducted in a high school (say about homework), in which kids and teachers are asked to answer all of the same questions, without identifying themselves as one or the other, and with everyone's vote counting equally. The teachers voice would be muted.

I

As for the question below: REDACTED

Change it to a single case report venue and allow selected publication in JVIM

I always planned to be a just a clinician. If I wasn't forced to write that paper I never would have done it. I learned so much while I wrote my published paper REDACTED but I learned even more when I tried to write the paper about my research which didn't go as planned. REDACTED I hated that study but I can say I learned a lot and I have always looked at journal articles differently since that study. It made me a much more critical reader. My eyes were opened to the "tricks" of statitistics and how difficult it is to get prospective data etc. It was truly invaluable to my education even though it seemed like a "frustrating flop" at the time. If you cannot publish a small paper or case report then there is a problem with the program. (Residents are not trying to get published in The New England Journal of Medicine.) Rather than eliminate the publication requirement, the programs must be critically reviewed.

I believe that the source of at least some of the problem is the sort of project given to/chosen by the candidate. If we work on that aspect, the rest of the journey will be more secure.

see above

Leave as is. I do not think modifications should be made.

Becoming a boarded internist is not supposed to be a walk in the park. Candidates should have to work hard, and be able to come up with a reasonable study - there are so many studies that still need to be done and can be done retrospectively (even if that is not the best study design, retrospective studies are still useful) without any financial burden. It is just time and motivation. If someone isn't motivated enough to do that, or a faculty member (or clinician at a private practice) isn't interested in helping then they should reconsider having a resident. I think this part of our training is very important. I am in private practice but would not have traded any aspect of my residency for doing less. I think it made me a much better, well rounded practitioner.

This is not a hurdle for a resident that is motivated to complete it. We need to stop diluting our requirements, and therefore stop diluting our candidates/Diplomates.

I am torn on this issue. I did a research project and published but I was not and remain uninterested in clinical research. That being said I am better for having gone through it.

Perhaps list of acceptable publications can be expanded; Mentors should begin early in the residency process In initiating this requirement.

It's just another "hoop" for a candidate to jump through, and many of the "studies" are poorly designed and not of much value. And since that is the case, they get rejected, further slowing a candidate's career.

Should probably be given increased importance and weight in future.

I think although the publication requirement is a pain, and may prolong credentialing, I think it was an important process to have to go through and thus should be retained as part of the process.

In the discussions on the list serve, the scientific publication requirement was derided as an academic exercise by some participants. Whether or not the specialist pursues a career in research & publication, going through the process of participating in a research project and writing it up for dissemination provides an invaluable exercise in further understanding the research that specialists should be reading on a regular basis. In my opinion, critical evaluation of the literature can be taught more easily to someone who has a working familiarity of this process.

Keep it for goodness sakes. Perhaps do away with the ability to publish review articles.

*In terms of marketability for employment, I do not think that having a publication increases or decreases marketability for those candidates seeking private practice jobs, but more/better publications does provide some advantage for those that might choose academia (whether that was the original goal or not). *Some training programs REDACTED will almost certainly continue to require research and or publications to complete the residency (which will be required to be boarded) even if the ACVIM does not.

I think residents have plenty of time for clinical training. Retaining the publication requirement broadens their mandatory training by their program and also the individual's potential for future research. The requirement also helps make sure diplomates are furthering the discipline through continued research.

It is difficult but an important rite of passage and separates the best of the best which is what diplomate status should be.

Although I was not excited about this requirement for my certification, I did learn many things that have been positive. I'm not sure that I would be thrilled about going through the process again, especially the review process (with some reviewers clearly not up to date on the science and some inappropriate and rude comments), but I have had some cases that I would consider writing up now that I have this knowledge.

It has value in going through the process of completing a project, writing a paper and contributing to the knowledge base. However, when residents have little or no guidance in how to do a project and paper, it can be an uphill battle. Especially with a busy clinic load. Also, when residents get handed an already-completed project then it has no value whatsoever.

The implications in terms of career is somewhat related to the resident's interest in pursuing academia vs private practice; however, I believe one should not be placed in disadvantage for a possible change in career paths later on based on his publication (or lack of). Going through the process of writing, submission and review is important to help clinicians become familiarized with the different bias that affect the scientific community. However, I believe that case reports should still be accepted as publications since in most situations, these cases do not occur frequently enough for larger studies and should still be reported/made aware for the remaining scientific community.

I do not think we should down-grade the requirements to become a specialist. Specialist means you should not just be good at only clinical skills and taking tests, but also being able to understand, appreciate ,and contribute to the science behind our specialty.

see above. IT was good idea when the College was founded. It has now outlived its usefulness and time taken could be better spent.

I want to see it retained.

Being able to critically read the scientific literature and to write clearly and logically are skills that we should all possess. I find this requirement of substantially more value than the examination which I would be happy if we dispensed with entirely.

If you eliminate the publication requirement most residents will never truly understand how veterinary medicine moves forward.."if not them who".

Only that, while I appreciate that it is an educational process, it would be nice to offer other alternatives that better fit non-academic/non-research career goals. I don't think the entire concept should be eliminated, however.

See above. REDACTED

Any resident should be able to find, research and write a case report, perform a retrospective study, or complete a hypothesis testing or observational study, or even a review article. Even a case report requires finding a suitable case, researching the literature, and communicating effectively in writing. I do not think we should have specialists who do not have this basic skill.

I think it should be reduced to accept co-author status. Much of the stated goals above can be achieved by residents that participate in a study its publication.

it should stay. i personally believe with all the listserve bantering--we are graduating too many internists (a) but (b) we are lessening our standards (ie in my perspective making it easier)--it is a privilege to be a specialist--not a right. Lowering expectations is following along with this new generation of entitlement and not working hard for the goal. A residency diploma should not be a "participation" medal--which some residencies are; we need the standards of publication and examination to keep the ACVIM moniker to those deserving of it.

The question "Was your credentialing delayed as a result of the publication requirement?" is biased.

REDACTED

I think we should enforce standards rather than keep making it easier. I personally think a candidate should not be able to sit for the exam until the paper is accepted for publication and feel we made a mistake getting rid of case reports and diluting down the exam so that the pass rate was more politically acceptable. I feel it you set the bar high those that are worthy will do everything they can to reach it and those that are not simply will . This strict adherence to standards makes achievement of ACVIM diplomate status really mean something rather than an natural exception for going thought the motions of a residency.

Do ALL resident publications contribute to the growth of knowledge in our specialty? No but that may be more the fault of the college and its diplomates. I realize that our specialty is now made up of more specialists in private practice than in academia and it seems that many of those in private practice feel that writing for publication provides no benefit to the candidate. (I don't really know if a disproportionate number of private practitioners vs academicians feel this way but that seems to be the way the debate falls when it comes up on the listserv) If we just want to train technically competent clinicians then writing for publication probably doesn't do much to achieve this goal. But, since "the mission of the ACVIM is to enhance animal and human health by advancing veterinary internal medicine through training, education, and discovery" I think that we should be doing more than just training people to be technically competent. I think the college and its diplomates need to do more to make research accessible to all; not just financially but with mentorship, statistical assistance, etc. I think that the private practice specialists are sitting on a veritable gold mine of possible research projects if we can find a way to tap into that.

I have come around to thinking that the most important skill we can impart to residents is the ability and desire to critically evaluate the literature. I think we can do that better via other means, such as rigorous journal clubs. We could also replace the manuscript requirement with specific training (a required workshop?) and an exercise for credentials that requires critical evaluation of the literature. That activity can also be tested in some way during the examination. Alternative scientific research and publication training opportunities (combined PhD programs, Masters programs, fellowships, workshops, etc) can be developed for academic-bound residents, which are becoming a minority.

It is essential that there is a lasting commitment to the publication requirement and the individual residents that are trying to fulfill this requirement. As REDACTED I/we have had to advocate for REDACTED that find their prior mentors unavailable or generally unhelpful in assisting the prior resident in completing their publication requirement even though these projects were started during residency. I feel that this should be part of a lasting exit interview and monitoring process and if residency programs are not supporting their prior residents through all aspects of the boarding process, even if these requirements go beyond the time of the residency proper, they should be help accountable and have their credentials revoked or be put on probation. I believe that the publication requirement is important on many levels and if a program is not willing to support that then they should not be allowed to train residents.

It would be nice that residents that do not have mentors that actively help them have another avenue in which to help gather ideas and support from. Ie when you apply to a residency it is difficult to tell whether help will be easily available to you. If there was a group of diplomates willing to help out if needed on ideas/help out side of the residency it would likely make a great deal of difference for some residents.

The greatest resistance will come from tenure track PhDs who use residents to do the footwork in generating publications.

I feel that both the publication and case reports both contribute to the ability to form logical thoughts and how to communicate them

I was very fortunate to have the opportunity to complete REDACTED. If I had relied on the research, it would not have been completed in time for credentials. REDACTED There was no funding through my department, and I was told that this was my responsibility. This created great stress during the residency that was not productive. Thankfully I received REDACTED but then needed to complete all work REDACTED during the last 12 months. This was incredibly difficult and not productive toward my clinical abilities. Although my REDACTED were of basic science use, it was my other publications that I think were of greater use in the clinical field.

I started my project and submitted it during my residency. With the review process with REDACTED, it was ultimately accepted after I finished, so it did delay my credentialing by a few months. I don't feel like that hurt me with respect to getting a job.

I have had one resident REDACTED who was just incapable of meeting the publication requirement. This individual passed the qualifier in year 2 and certifier in year 3 and was an excellent clinician and mentor for Interns. Eventually this person met the publication requirement REDACTED, but I don't know what value there was in denying this individual their Diplomate status. The publication that was finally accepted did not add significantly to the literature. For so many reasons, I would like to see many parts of residency programs improved, but I don't think continuing the publication requirement is necessary. In regards to the last question, one of my publications was delayed because the REDACTED on the study just would not complete his portion of the study. Despite repeated phone calls and emails for more than a year, it required me to enlist the help of a higher authority to force him to complete his portion. Even then I had to ask the REDACTED for additional information as his first effort was of low quality and insufficient for publication. This dependency on factors outside of the control of the Resident is another major reason the publication requirement should be eliminated.

Outdated requirement from a time when most residents were are universities and planned for academic career.

As a SAIM diplomate, I do think that it is critical that we have excellent skills in reading and critiquing the scientific literature. I think that we can be successful in our role in the veterinary community without publishing ourselves.

As an REDACTED, it is sometimes stressful to push a resident to obtain funding, complete a project, prepare/submit a manuscript, and address revisions for publication prior to the resident turning their focus solely to studying for boards. Additionally, if the manuscript is rejected this sets off another unique firestorm which often contributes to the resident not meeting their requirements. Some journals have discussed offering a pre-approval process for resident manuscripts to ensure the work to be done would be accepted for publication in an effort to minimize resident project rejections. It would likely be helpful if JVIM could have a "resident research" section or online supplement to the journal that facilitates resident publications or acceptable case reports. I think we all accept that some resident projects are not going to be ground-breaking information, but we can't require them to do something without also doing everything possible as a College to ensure they have every opportunity to meet the requirement. I also sympathize with colleagues REDACTED overseeing residents. While it is part of my job REDACTED to put up with navigating the standard manuscript review process, overseeing the publication process for every resident in private practice was a bit more of a strain.

As the "Maintenance of Credentials" mandated by the ABVS rolls out the College can use MOC as a stick/carrot to push publication. Of course all those on an academic track will have tenure or a true desire to publish pushing them along. If we thought the ultimate goal of ACVIM was extraordinary care for animals, we would not clutter up the clinical training years with research/publication requirements. Those efforts should be left to those who wish to pursue PhDs or other appropriate credentials.

I strongly recommend kerpibg the requirement.

I think I said enough. Good luck with this.

I would rather it were replaced with a required course in clinical epidemiology/evidence-based medicine, but that would be impossible to implement across the board, so the publication requirement should remain as a (poor) proxy. Thinking that the publication requirement is about preparing residents for their careers is disingenuous (what percentage have research-based appointments?) except in the context of a clinician understanding the scientific process.

I have a question instead of a comment: Why are you trying to dumb down the requirements to become an ACVIM diplomate? The problem is not the publication requirement. The problem is with the mentors not wanting to do the work to properly prepare candidates. It also related to flaws in the design of their program. REDACTED. Residents need to be given time for research and publication instead of just working in the clinics and producing income for the practice. We continue to dumb down the requirements because it is less work for the mentors to just pass people along and keep them working long hours in the clinics. If that is what you all want to do, fine. However, this is leading to poor quality diplomates. If someone wants to have a residency program, it is incumbent upon them to do the work necessary to insure high quality diplomates. This includes making sure the candidates are given time and support to fulfill their publication/research requirements and on time. An understanding of the scientific method, development of writing and communication skills and contribution to the veterinary literature are very important steps in the development of ACVIM specialists. REDACTED

Over the past 2 decades, the SAIM College has shown a clear path to making the credentially process easier and without impediments. The elimination of Case Reports, the elimination of time constraints, the disjoining of examinations (you can't take the Certifier until you pass the General... this has been removed), the disjoining of examinations and Credential Packet submission, etc. have all been performed under the auspices of 'making things fair' for the candidate. However, in the process, we have been consistently making is easier for every and any candidate to become a Diplomate. Now, there is a push to remove the publication requirement and to increase the 'pass rate' on the examinations. We, as a college, need to stop 'lowering the bar' to meet the poor performing residents. Since residency programs can not seem to police themselves, then we must use the Credentials Process to maintain standards in our College.

I truly think this is an important part of credentialing and those who do not complete the publication either do not have mentors who support them successfully or are not truly motivated to get the requirements done.

It is a big mistake to take it away. The requirements continue to be watered down year after year. Credentials are supposed to be a challenge.

I feel this is a top down issue. If you change paper requirements to review article or case reports it would give residents a taste for writing without needed the research mentorship. Do you need to research to be a good internist, absolutely not! But if you know how to read journals and look up resources it helps you be a good internist. As we have both tenure and clinical track faculty as well as private practice residencies we need to keep in mind unless residents need little guidance they will need help to do research (i.e. foster ideas and lab space). I sure as heck would have loved to do primary research but just did not have the realistic help that I personally would have neeeded. I know other residents are more self motivated but as motivated as I was I needed more direction and possibly consequences for not getting things done.

There is a tremendous gap between academic training program and private practice training. Without going in to much detail here, strong private practice program exist with tremendous case loads and almost no plausible downtime. Over the last decade, we have seen more and more ways to "accept" publications of quality, some of which remains unpublished 3-4 years after acceptance. I think it is a requirement that is archaic and prevent great candidates from getting boarded or delaying their abilities to become board-certified with tremendous financial consequences

For the place that I completed my residency it was required by the institution that we have REDACTED in order for us to successfully complete the residency and get our certificate . So in comparison to that the ACVIM requirement was very mild and not a big deal to me. I had my publication requirement for ACVIM met before submission of my credentials. I do not feel that the publication requirements of my residency institution took away from my clinical training or board preparation/study in any way. I actually thought it was extremely helpful for training me to critically evaluating the literature which allowed me to apply that information better to my clinical cases as well as study for boards.

Often the publication requirement helps the mentor if they are in an academic practice.

It was, unfortunately, the aspect of board certification that did slow/prevent me from becoming certified immediately upon passing the specialty exam, & not solely due to my own fault/failing. REDACTED Despite that, I do think there is value to the publication requirement; I just wish that I had known there would be the potential for such delay that was out of my hands, & perhaps I would have gotten started sooner on writing it. REDACTED I do think that those diplomate mentoring resident research projects need to be more hands-on in that respect.

Strongly believe that it is valuable and should be retained, and made a priority for resident training; as much a priority as learning how to use an endoscope. It is equally, if not more, important.

I strongly believe it should be retained. If a residency cannot meet the research training requirement, then I do not believe that it is providing a fully rounded training program to generate critical thinkers.

I think it would be a HUGE mistake to get rid of the publication requirement. Our specialty will not grow without fostering the kinds of skills developed as a result of this requirement. Even specialists in private practice have the potential to contribute if well trained.

It should remain intact at all costs. A good residency program can provide oversight for this requirement to be completed easily during the residency.

Performing research and writing manuscripts is under the control of the program and the candidate. However, and this is journal dependent, there can be quite a delay to get an accepted publication. As a reviewer, I have seen some manuscripts with merit rejected easily, with no ability to revise, in journals in which the submission rate is high. Unless a resident submits a paper in the second year, they are unlikely to get their manuscript published prior to boards if there are revisions/resubmissions, because of their limited off clinic time. Would a poster or abstract presentation at ACVIM or similar conference serve a similar purpose? I hate to see residents wait a year or more to get boarded, especially because there time is even more limited once they leave their residency programs.

In my experience REDACTED, it is my observation that good to excellent residents do not struggle with publication requirements. The publication requirement often seems to be most difficult for residents who are adequate to poor.

work should be done DURING the residency

I think its a good experience. It is hard to evaluate the literature until you actually understand what it involves and its limitations.

Q12 - The publication that I submitted for credentialing purposes was based on studies performed:

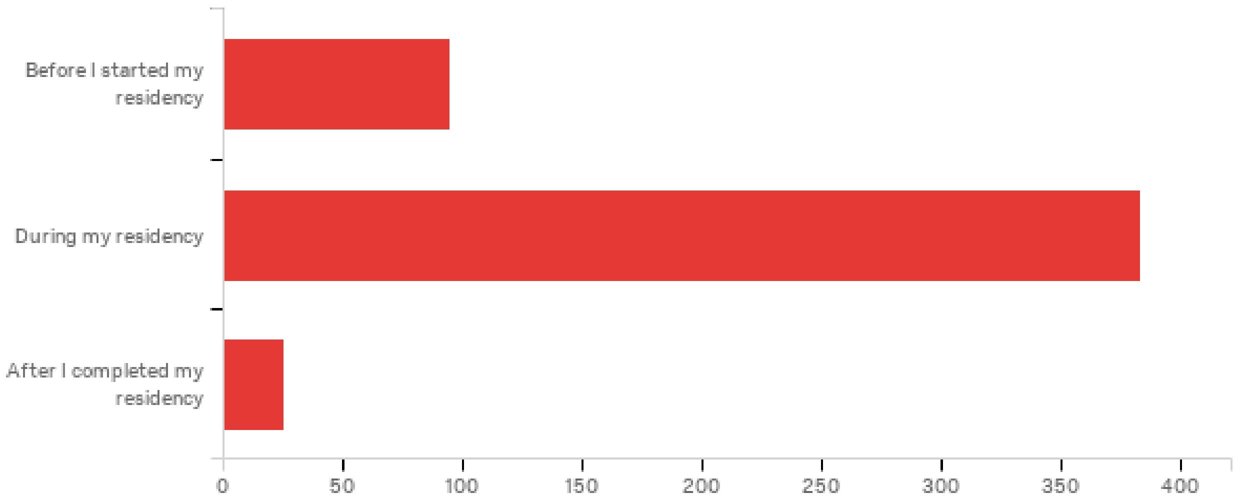

Answer	%	Count
Before I started my residency	18.73%	94
During my residency	76.29%	383
After I completed my residency	4.98%	25
Total	100%	502

Q13 - Was your credentialing delayed as a result of the publication requirement?

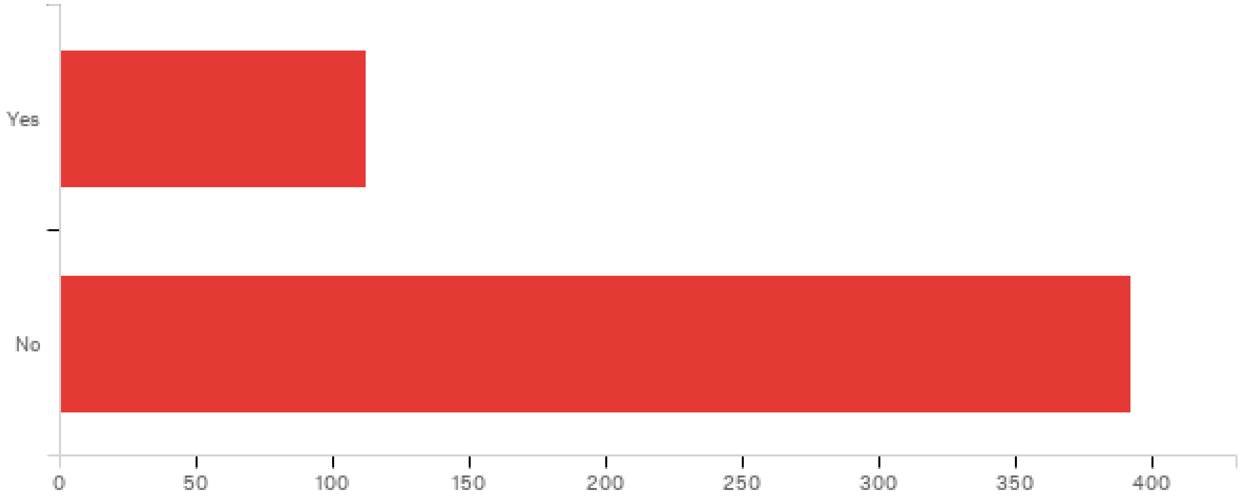

Answer	%	Count
Yes	22.22%	112
No	77.78%	392
Total	100%	504

Q14 - What was the reason for the delay? (Select all that apply)

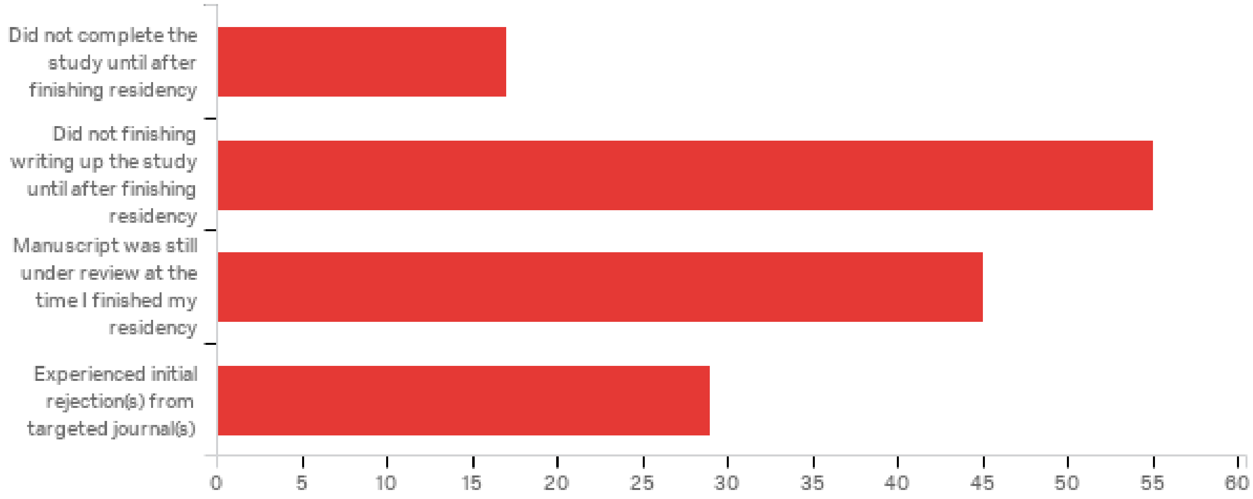

Answer	%	Count
Did not complete the study until after finishing residency	15.32%	17
Did not finishing writing up the study until after finishing residency	49.55%	55
Manuscript was still under review at the time I finished my residency	40.54%	45
Experienced initial rejection(s) from targeted journal(s)	26.13%	29
Total	100%	111

Q15 - I believe the publication I submitted for ACVIM credentialing purposes was:

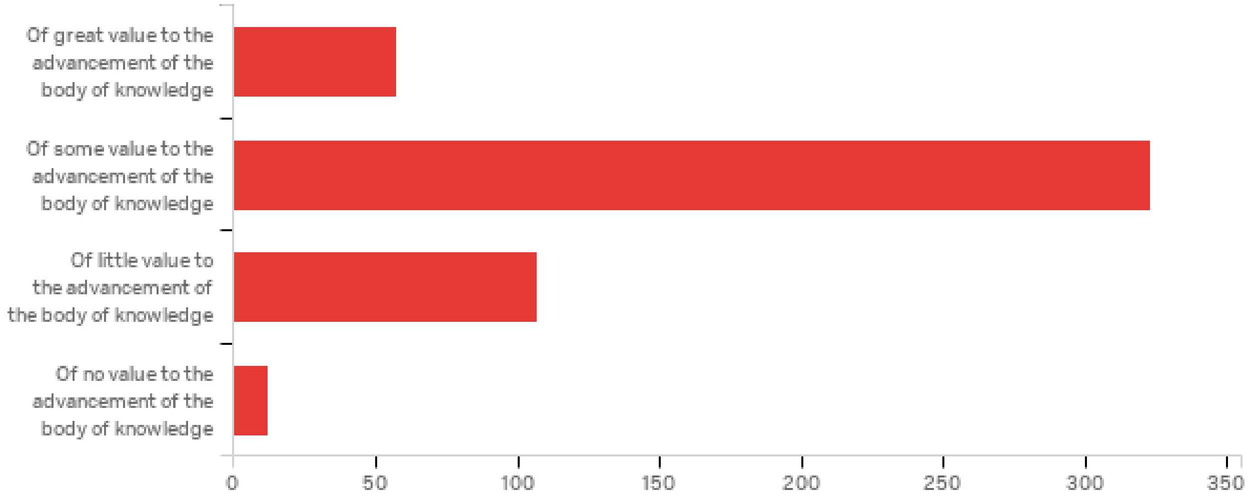

Answer	%	Count
Of great value to the advancement of the body of knowledge	11.42%	57
Of some value to the advancement of the body of knowledge	64.73%	323
Of little value to the advancement of the body of knowledge	21.44%	107
Of no value to the advancement of the body of knowledge	2.40%	12
Total	100%	499

Q16 - Area of practice:

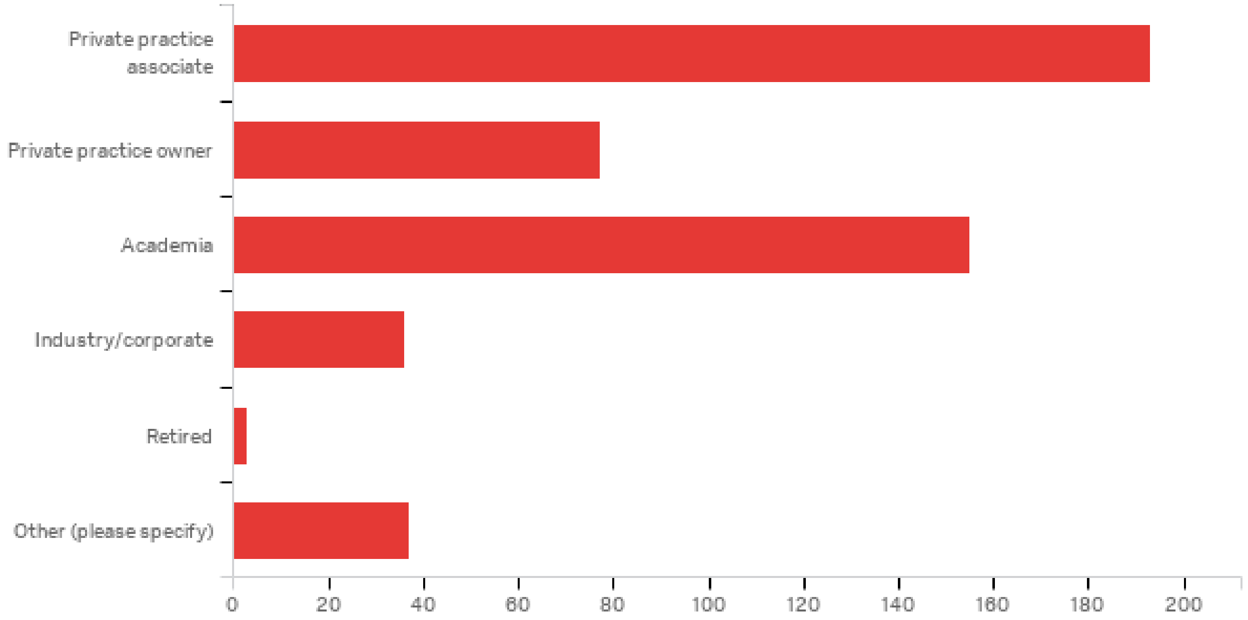

Answer	%	Count
Private practice associate	38.52%	193
Private practice owner	15.37%	77
Academia	30.94%	155
Industry/corporate	7.19%	36
Retired	0.60%	3
Other (please specify)	7.39%	37
Total	100%	501

Other (please specify)

Other (please specify)
ALL REDACTED

Q17 - Type of residency:

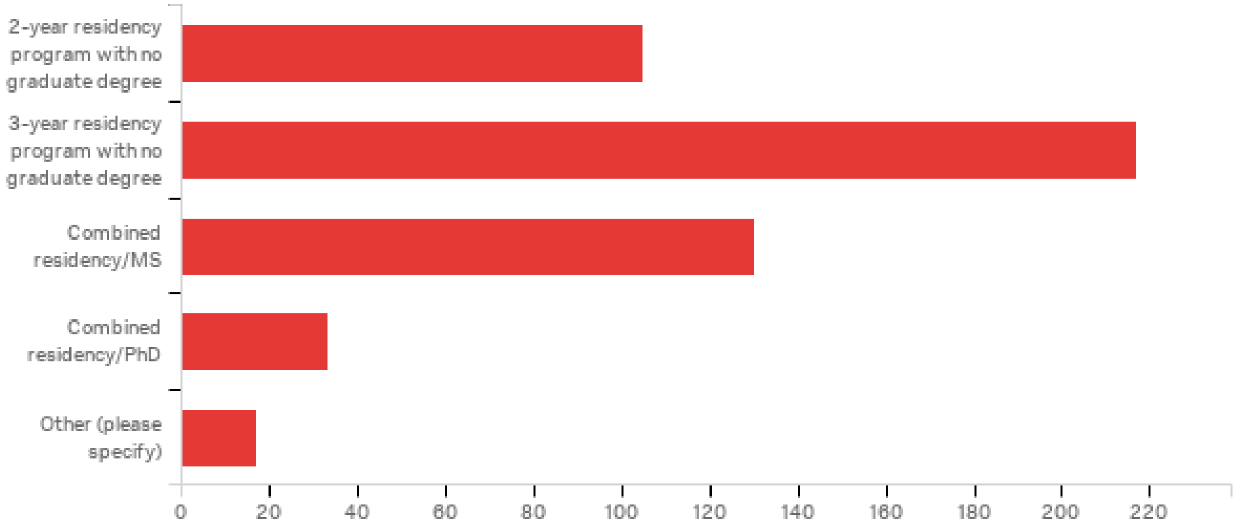

Answer	%	Count
2-year residency program with no graduate degree	20.92%	105
3-year residency program with no graduate degree	43.23%	217
Combined residency/MS	25.90%	130
Combined residency/PhD	6.57%	33
Other (please specify)	3.39%	17
Total	100%	502

Other (please specify)

Other (please specify)
ALL REDACTED

Q18 - Sex

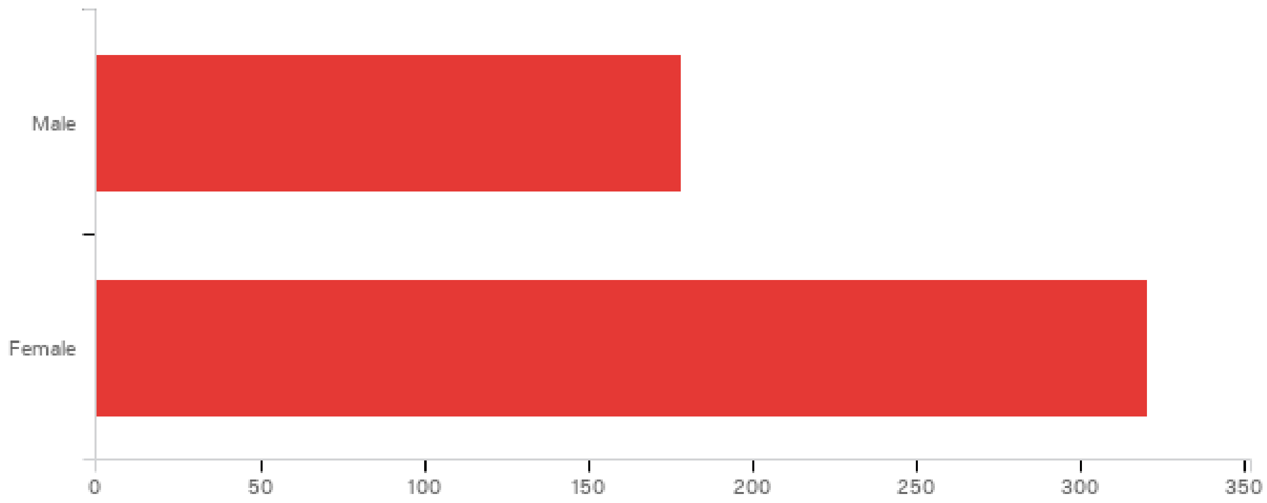

Answer	%	Count
Male	35.74%	178
Female	64.26%	320
Total	100%	498

Q19 - What race/ethnicity do you best identify with?

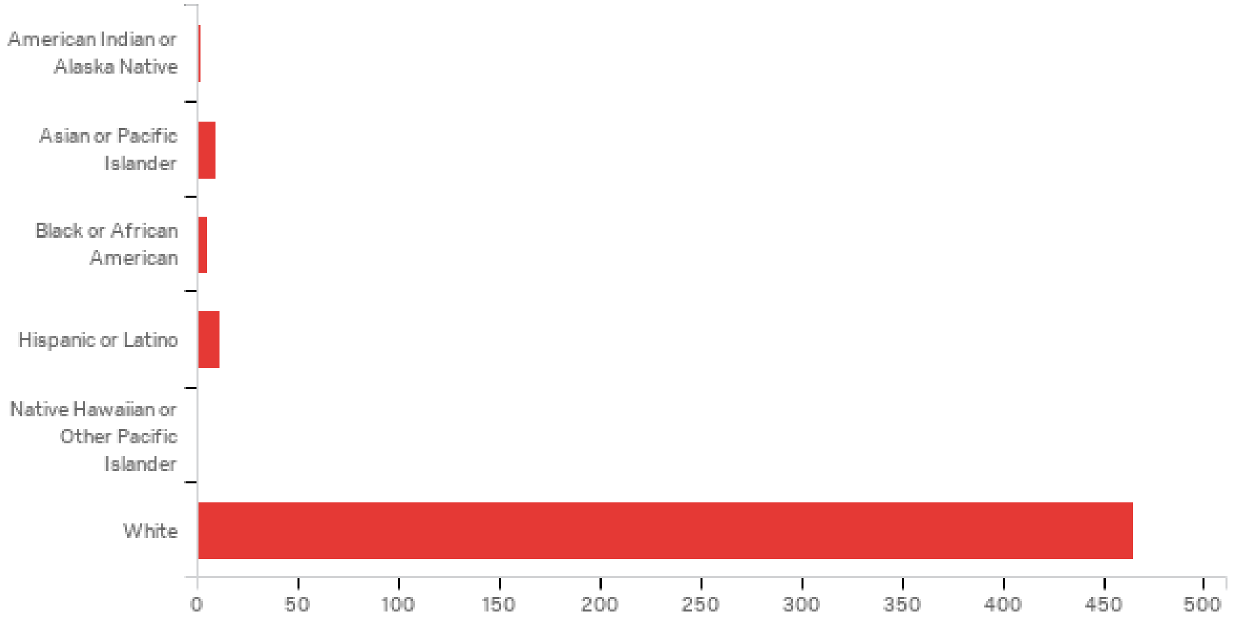

Answer	%	Count
American Indian or Alaska Native	0.41%	2
Asian or Pacific Islander	1.83%	9
Black or African American	1.02%	5
Hispanic or Latino	2.24%	11
Native Hawaiian or Other Pacific Islander	0.00%	0
White	94.51%	465
Total	100%	492

TypeofPractice

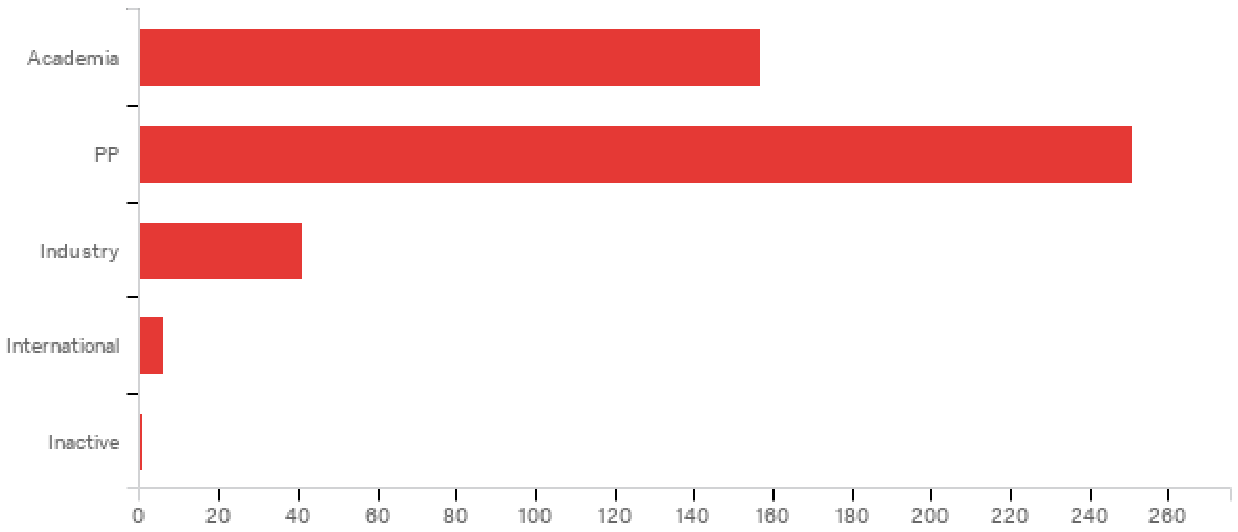

Answer	%	Count
Academia	34.43%	157
PP	55.04%	251
Industry	8.99%	41
International	1.32%	6
Inactive	0.22%	1
Total	100%	456
