# Supplementary material for: Perceptions and attitudes of Small Animal Internal Medicine specialists toward the publication requirement for board certification
Source: J Vet Intern Med. 2020 Feb 7;34(2):574–80. doi: 10.1111/jvim.15717 (PMC7096663; doi:10.1111/jvim.15717)
Supplement: Supplementary file 2 — Data S2 [file JVIM-34-574-s002.pdf]

Go to “Web of Science” through NCSU libraries.

https://clarivate.com/webofsciencegroup/solutions/web-of-science/

For individual publication citation(s):

Search: title

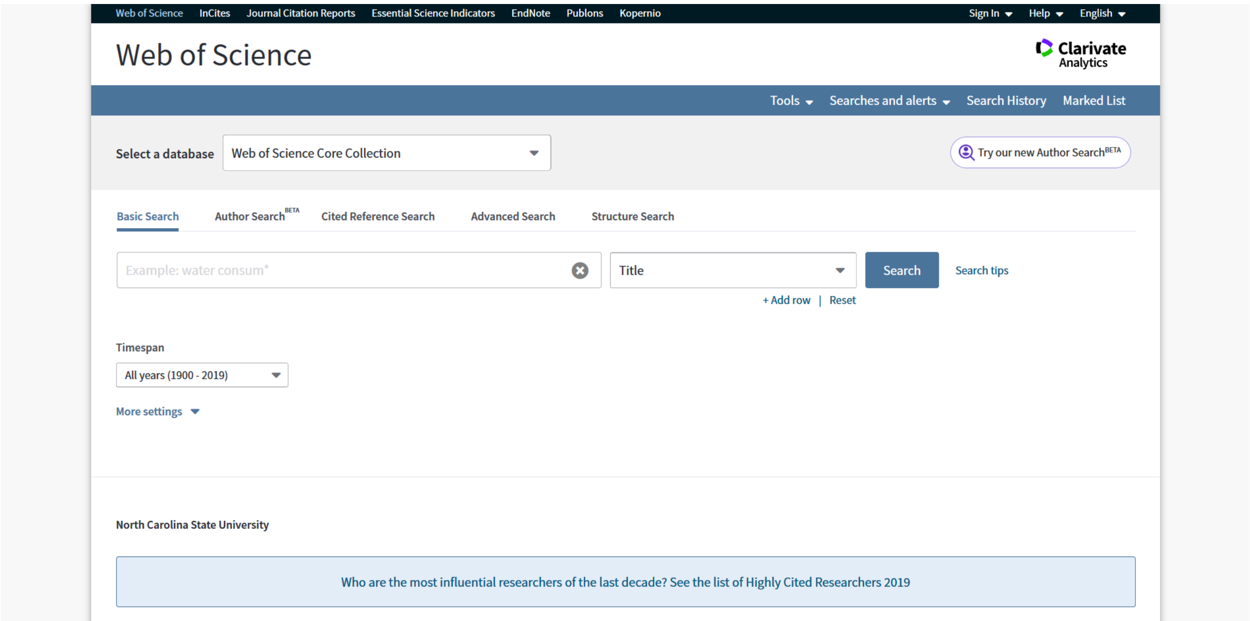

Record result

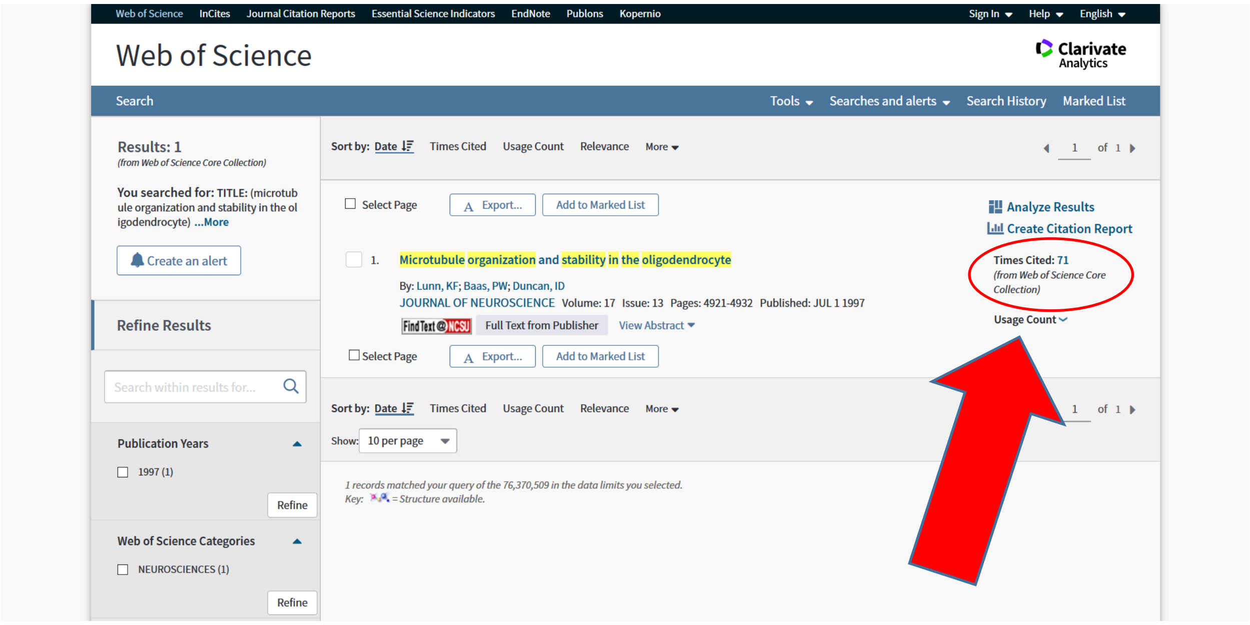

For journal search:

Step 1

Search: Publication Name, Limit timespan to the same year the article of interest was published

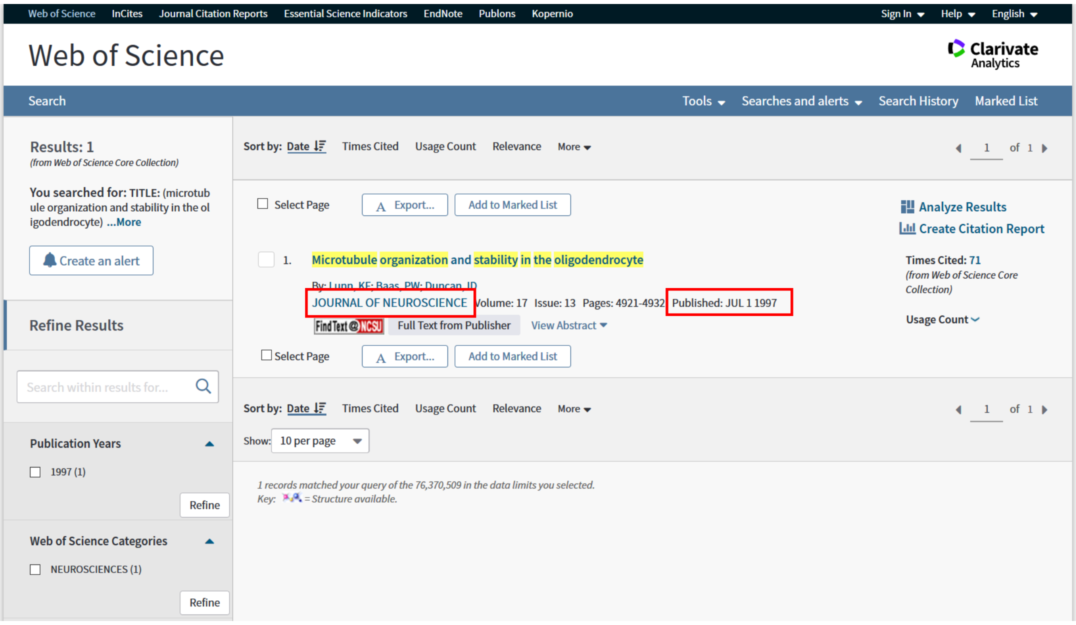

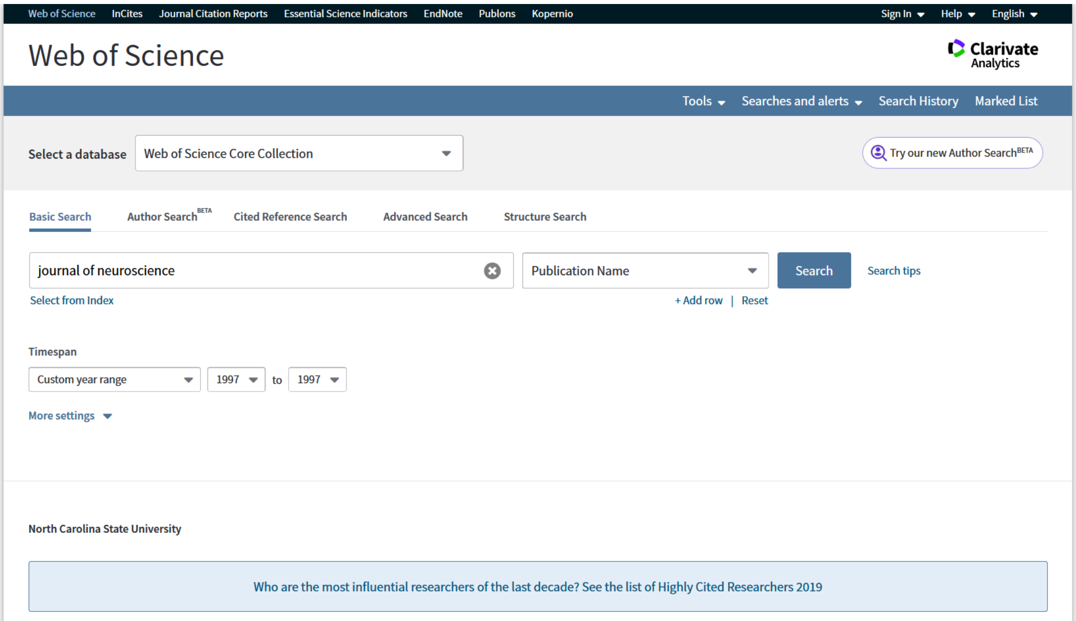

Step 2

Once you have the results, click on the “create citation report”

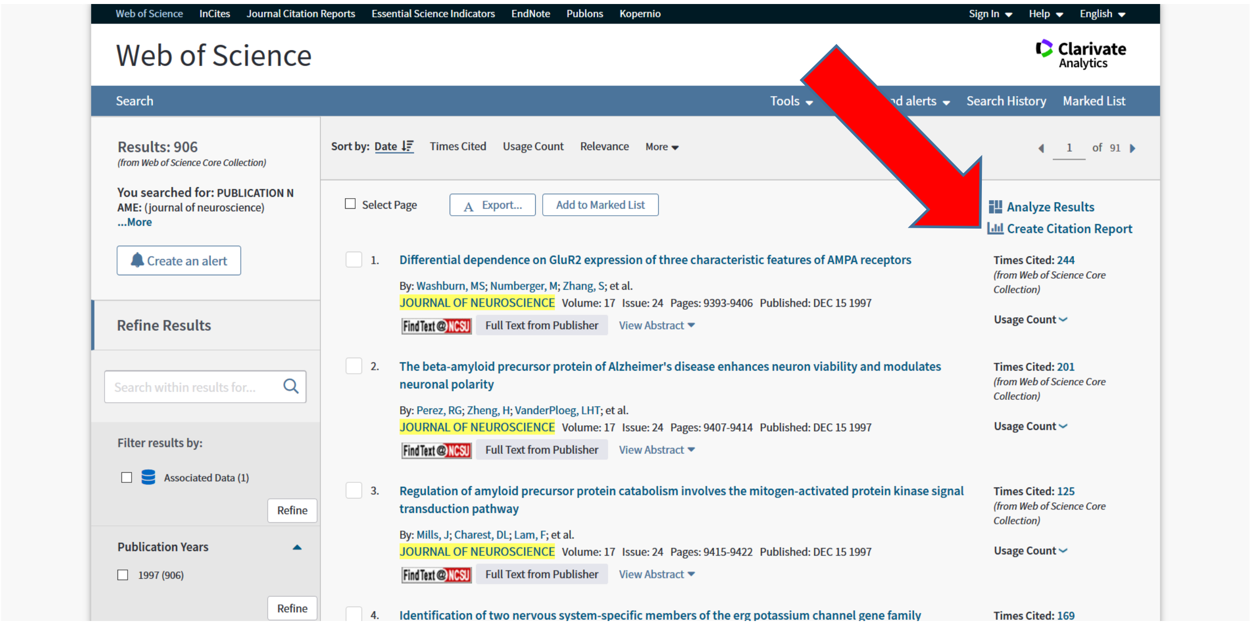

Step 3

Select save to excel file.

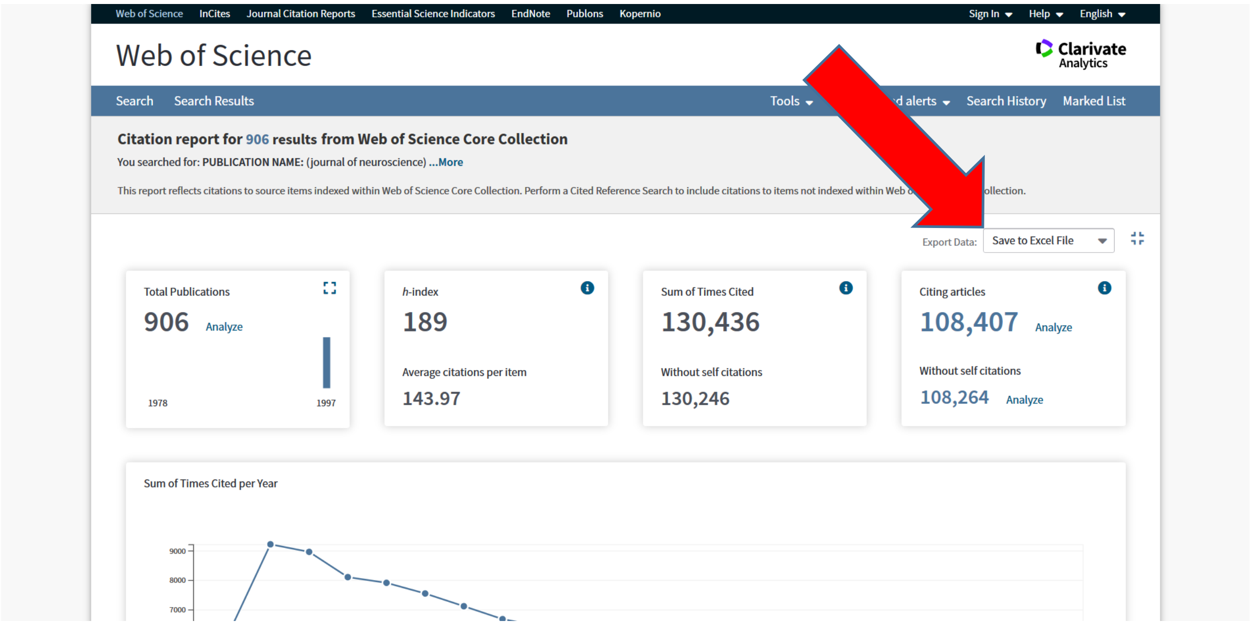

For journals with >500 publications: Save them 500 at a time, then combine results into one excel sheet.

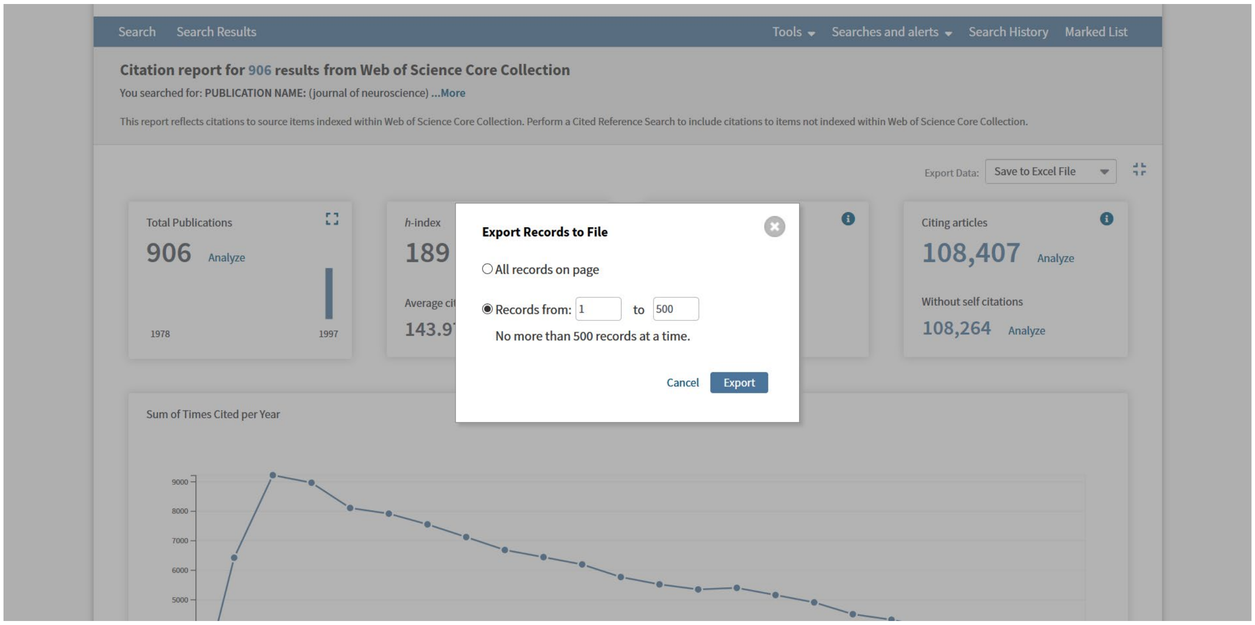

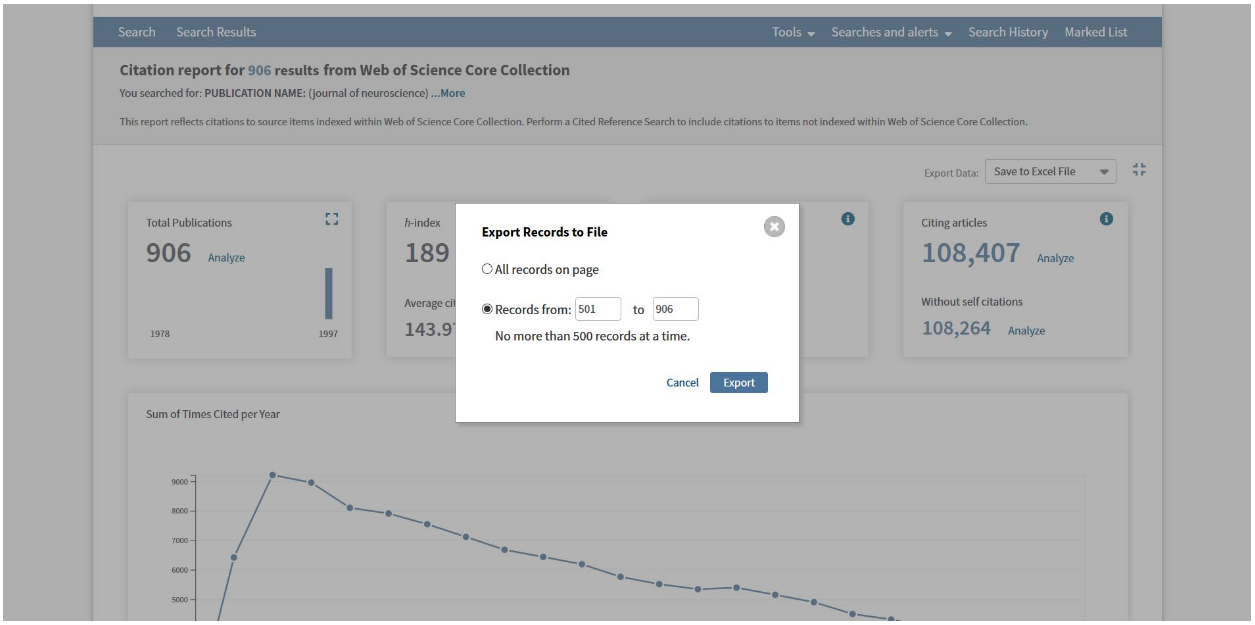

Step 4

Only Journals with electronic access to full articles through the NCSU library were included.

All publications in that journal from that year were manually reviewed and we:

1. Included publications explicitly stated in the GIG: Research articles, Case reports and Review Articles
2. Excluded all other publications: ECG of the month, What's your diagnosis, short/brief communications, Letters to the editor, Commentaries, etc.

Step 5

Determine the quartiles, limits and median for the included articles for each journal/year:

Use the following formula in excel to determine quarile data=CONCATENATE(QUARTILE(array,0),", ", QUARTILE(array,1),", ", QUARTILE(array,2),", ",QUARTILE(array,3),", ",QUARTILE(array,4))

array input = column with citation numbers

Results will be reported similar to this: 0, 6, 14, 26, 107

First integer = lower limit
Second integer = First quartile (25th percentile)
Third integer = median
Fourth integer = Third quartile (75th percentile)
Fifth integer = Upper limit

Step 6

Determining quartile for individual CredPubs:

IF number of citations for Credpub is < second integer THEN lower quartile

IF number of citations for Credpub is > fourth integer THEN upper quartile

IF number of citations for Credpub is ≥ second integer and ≤ fourth integer THEN interquartile range (middle 50%)
