# Supplementary material for: Perceptions and attitudes of Small Animal Internal Medicine specialists toward the publication requirement for board certification
Source: J Vet Intern Med. 2020 Feb 7;34(2):574–80. doi: 10.1111/jvim.15717 (PMC7096663; doi:10.1111/jvim.15717)
Supplement: Supplementary file 3 — Data S3 [file JVIM-34-574-s003.pdf]

Website SOP

Search for SAIM Diplomates using the "Find A Specialist" tool (please note that screen captures were taken 12/018/2019)

https://vetspecialists.com/

Select Veterinary Internal Medicine AND Small Animal Internal Medicine AND ALL

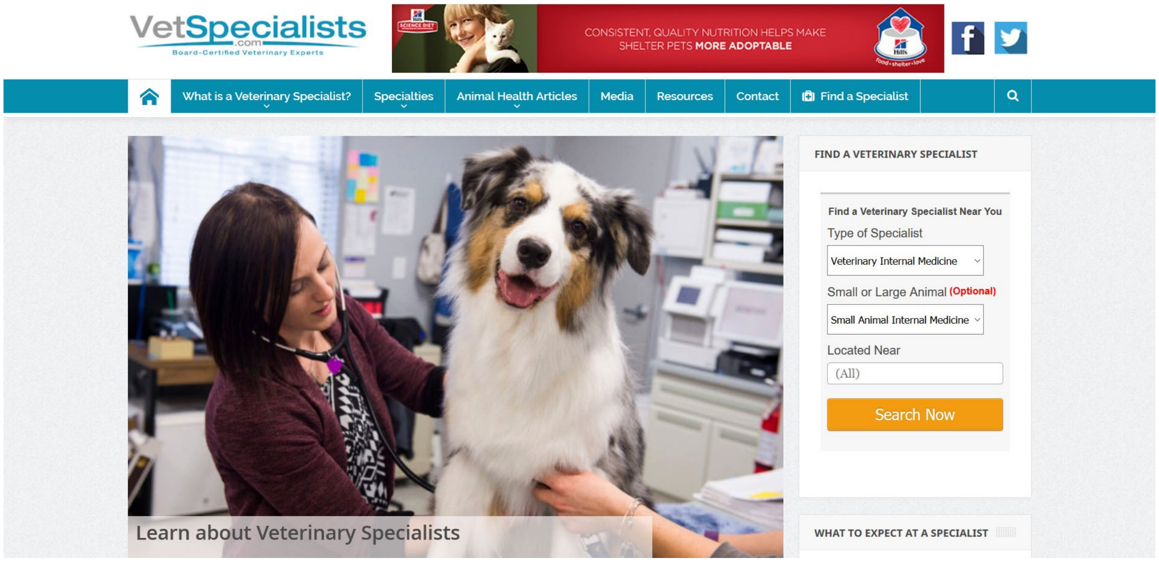

Click on "View Listing" for each individual

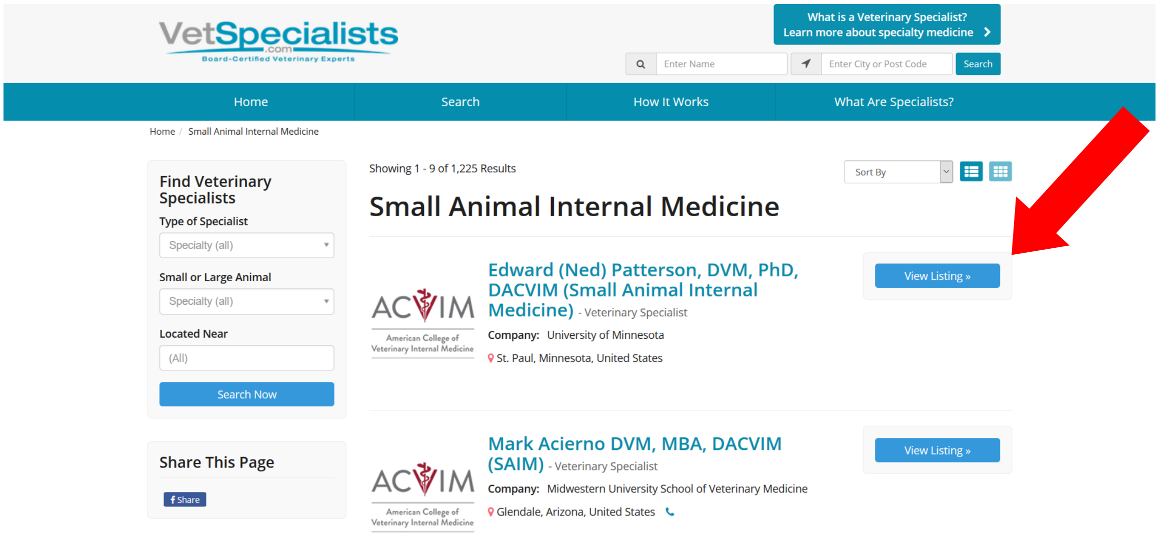

If the individual's company or website listing IS a university or company: Exclude

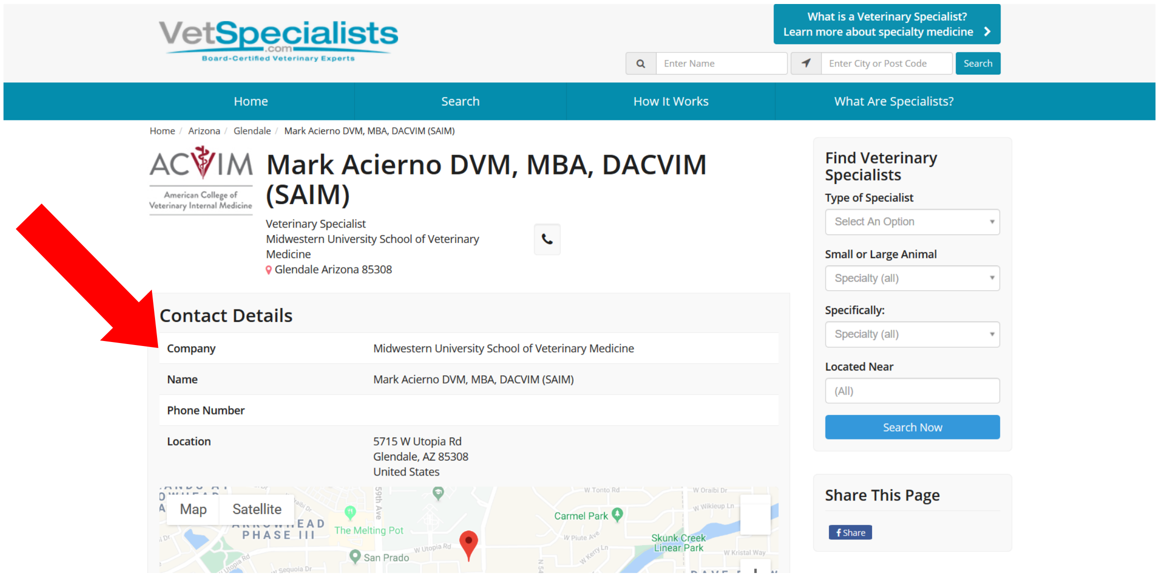

If the individual's company or website is NOT a university or company:

1. Visit website
2. Website is perused for whether or not they mention research or publication for their specialists
3. Whether or not and other SAIM Diplomates are employed at the same practice
4. Note sentiment as positive or negative if research or publication is mentioned

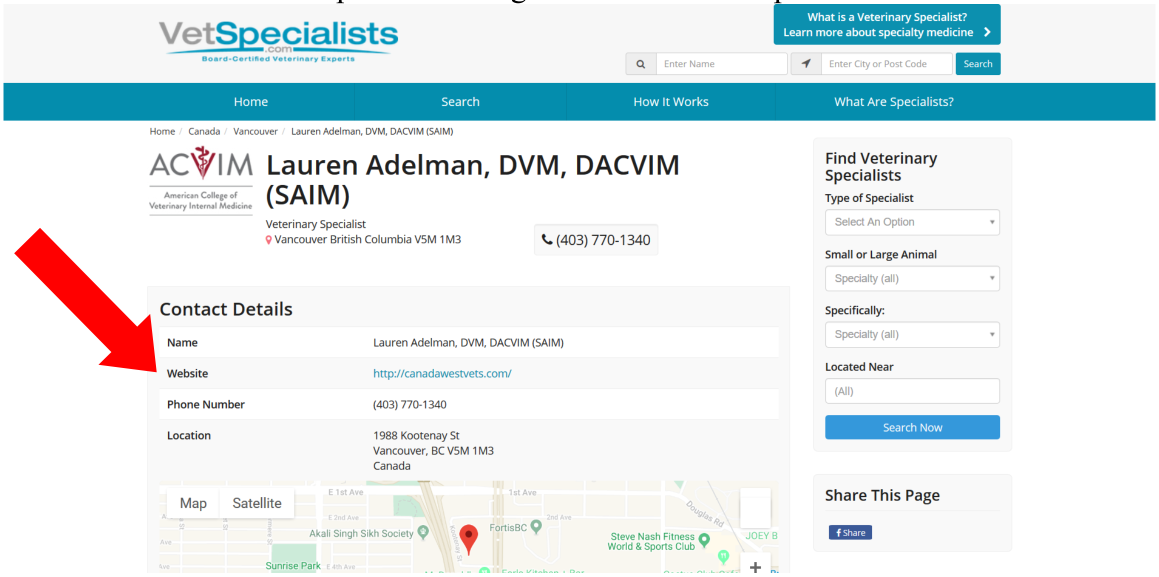

If an individual does not have a company or website listed:

1. Copy individuals name and paste into search engine (Google.com) including one or more additional terms (DVM, VMD, DACVIM, ACVIM, SAIM or Internal Medicine)
2. If no employer website is detected (page one of results): abandon search
3. If an employer website is detected
4. Website is perused for whether or not they mention research or publication for their specialists
5. Whether or not and other SAIM Diplomates are employed at the same practice
6. Note sentiment as positive or negative if research or publication is mentioned Not sentiment as positive or negative if mentioned
