# Supplementary material for: Perceptions and attitudes of Small Animal Internal Medicine specialists toward the publication requirement for board certification
Source: J Vet Intern Med. 2020 Feb 7;34(2):574–80. doi: 10.1111/jvim.15717 (PMC7096663; doi:10.1111/jvim.15717)
Supplement: Supplementary file 4 — Data S4 [file JVIM-34-574-s004.pdf]

Journal
Am J Physiol Heart Circ Physiol.
Am J Physiol.
Am J Vet Res
ANTIMICROBIAL AGENTS AND CHEMOTHERA
Aust Vet J.
Blood
can j vet res
Can Vet J
Clin Exp Allergy
Domest Anim Endocrinol.
international journal of applied research in vet med
International Journal Of Environmental Research And Public Health
Irish Vet J
J Am Vet Med Assoc.
J Com Path
J Feline Med Surg.
J Neuro Sci
J Small Anim Pract.
J Urol.
J Vet Cardiol.
J Vet Diagn Invest.
J Vet Intern Med.
J Vet Pharm and Ther
J Vet Surgery
JAAHA
Journal of Neuroscience
JVECC
Neuromuscul Disord.
NZ Vet J
Radiat Res.
Res vet sci
Teratology
TheVetJ
V clin north am small anim pract
Vector Borne Zoonotic Dis.
Vet Clin Path
Vet Comp Oncol.
Vet Immunol Immunopathol.
Vet Microbiol.
Vet Parasitol.
Vet Pathol.
Vet Radiol Ultrasound
Vet record
Veterinary Medicine
